# Regioselective Halogenation of BOPPY Fluorophores and Subsequent Diversification via Cross-Coupling and Aromatic Nucleophilic Substitution Strategies

**DOI:** 10.1021/acs.joc.5c03121

**Published:** 2026-03-10

**Authors:** Sebastian O. Oloo, Petia Bobadova-Parvanova, Alexis A. Lueders, Mina Kim, Frank R. Fronczek, Kevin M. Smith, Maria da Graça H. Vicente

**Affiliations:** 1 Department of Chemistry, 5779 Louisiana State University, Baton Rouge, Louisiana 70803, United States; 2 Department of Chemistry and Fermentation Sciences, 1801 Appalachian State University, Boone, North Carolina 28608, United States

## Abstract

The regioselective mono- and tribromination of a BOPPY dye followed by its reactivity under Pd-catalyzed cross-coupling and nucleophilic substitution reactions are reported. The brominated BOPPYs undergo Pd(0)-catalyzed cross-couplings with a variety of boronic acids and organotin reagents to give the corresponding products in good-to-excellent yields. Nucleophilic aromatic substitutions occur both on the mono- and tribromo-BOPPYs. The reactivity order of the latter is C3-Br > C1-Br > C2-Br, while in the cross-coupling reactions using Pd­(PPh_3_)_4_, it is C1-Br > C3-Br > C2-Br, likely due to steric interaction upon Pd­(PPh_3_)_2_ insertion into the C3-Br bond and the slightly longer and weaker C1-Br bond. The functionalized BOPPY derivatives showed pronounced bathochromic shifts in their absorption and emission bands compared with the starting compound, and fluorescence quantum yields depend on the nature and position of the substituent.

## Graphical abstract

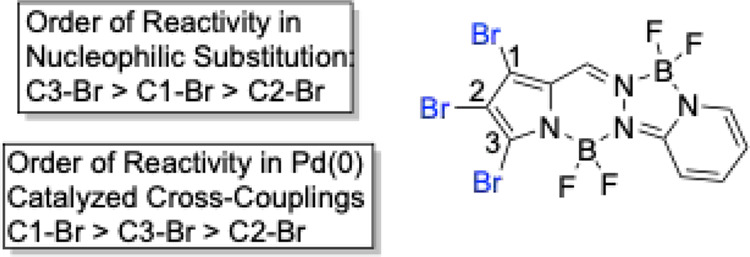

## 1 Introduction

Boron-dipyrromethene (BODIPY) dyes have emerged as a foundational platform in the development of materials for application in bioimaging, optoelectronics, sensing and phototherapy.^1–4^ Their high fluorescence quantum yields, tunable absorption/emission profiles and remarkable chemical and photostability make them a mainstay in organic dye chemistry. The core structure of BODIPY is highly amenable to chemical modification, allowing for systematic tuning of electronic and steric properties to match specific application requirements.^5,6^ Studies have shown that electronic manipulation at the α and β positions of the BODIPY core, whether through electrophilic substitution, transition metal-catalyzed cross-coupling reactions or nucleophilic aromatic substitution, can lead to significant changes in the spectral properties of the compounds.^6^

Recently, the scope of the BODIPY chemistry has been extended to include novel analogues, such as BOPHY, BOPYPY, BOAPY and BOPPY.^7–9^ These newer scaffolds introduce a hydrazine linkage within a dipyrrolic or heteroaromatic framework enabling stabilization by two BF_2_ units. The resulting rigidified π-backbones offer several advantages over BODIPY. They often display large Stokes shifts, in the range of 40–100 nm, which decrease reabsorption losses, exhibit high solution and solid-state fluorescence and high molar extinction coefficients, in addition to enhanced photostability due to the presence of the anchoring two BF_2_ units.^7^

Among the bisBF_2_ dyes, unsymmetric BOPPY analogues characterized by a fused pyridyl ring at the β-position, were first reported by Hao and Jiao et al. in 2018 ^10^ and have since been studied for various applications due to their synthetic versatility. Ono et al.^11^ developed flag-hinge BOPPY-dimer chromophores derived from diformyl-2,2′-bipyrrole, and observed strong circularly polarized luminescence with high asymmetry factors and fluorescence quantum yields. BOPPYs have also been used for labeling D_2_ and D_3_ dopamine receptor ligands, exhibiting low to excellent quantum yields.^12^ Despite these advancements, targeted modification of BOPPYs remains underexplored, particularly in the context of regioselective halogenation followed by orthogonal cross-coupling and nucleophilic substitution reactions.

Herein, we report a systematic approach to the synthesis of a series of BOPPY derivatives via regioselective halogenation reactions, followed by Pd-catalyzed cross-coupling reactions and nucleophilic aromatic substitutions. These transformations allowed us to generate a structurally diverse library of fluorophores. Furthermore, per-bromination of all the pyrrolic sites allowed the investigation of the regioselectivity of the cross-coupling and nucleophilic substitution reactions. The resulting functionalized BOPPYs display a wide range of photophysical properties, including extended absorption into the visible region, large Stokes shifts and variable fluorescence quantum yields (Φ_F_ up to 1). Notably, the introduction of aryl or heteroaryl substituents leads to enhanced π-conjugation and modulates the BOPPY’s excited-state behavior, while electron-deficient substituents induce fluorescence quenching.

## 2 Results and Discussion

### 2.1 Synthesis and Structure Characterization

The synthesis of the BOPPY chromophore was accomplished following a stepwise condensation strategy between pyrrole-2-carboxaldehyde and 2-hydrazinopyridine in the presence of a catalytic amount of *p*-toluenesulfonic acid (PTSA), as previously reported.^10^ The intermediate product readily underwent complexation with boron trifluoride diethyl etherate (BF_3_·OEt_2_) under basic conditions (DBU), affording the parent BOPPY **1** framework in 47% isolated yield (Scheme 1). The moderate yield reflects possible steric hindrance at the hydrazone linkage and competitive oligomerization pathways typical of pyrrolic condensations.

**Scheme 1:**
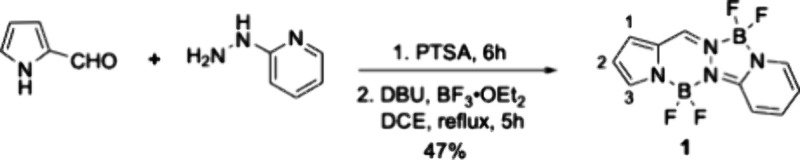
Synthesis of BOPPY **1** with Numbered Pyrrolic Positions

The regioselective halogenation at the pyrrolic sites was explored using copper­(II) halides in acetonitrile^13^ (Scheme 2). Treatment of the BOPPY **1** with an excess of CuCl_2_·2H_2_O (5 equiv) resulted in the isolation of BOPPY **1a** in 54% yield. The structure of BOPPY **1a** was easily confirmed by ^1^H NMR spectroscopy due to the disappearance of the α-pyrrolic proton at 7.82 ppm. Similarly, using an excess of CuBr_2_ (3.1 equiv), the monobrominated derivative **1b** was isolated as the major product in 58% yield. In both cases, the halogenation reaction proceeded with high regioselectivity at the α-pyrrolic position over the other potential sites on the BOPPY framework. Although the most electron-rich site at the BOPPY periphery is the 2-position, as indicated by the calculated molecular electrostatic potentials MESPs (see the Supporting Information, Figure S66) the halogenation using copper­(II) chloride or bromide occurred regioselectively at the α-pyrrolic position. This result indicates a reaction mechanism that involves the formation of a BOPPY cation radical by single-electron transfer, followed by nucleophilic addition of halide ion. Such α-pyrrolic position regioselectivity in the presence of CuCl_2_ has been previously observed in the case of 8­(*meso*)-aryl-BODIPYs, but not with CuBr_2_; in the latter case the 2-pyrrolic position was brominated instead, presumably due to the *in situ* formation of Br_2_ and subsequent electrophilic bromination.^13,14^ Indeed, the calculated MESPs for BOPPY **1** cation radical (see the Supporting Information, Figure S66) show that the α-pyrrolic carbon has the least negative MESP (−14.530 au vs −14.573 au for position 1 and −14.590 for position 2), therefore is the most reactive toward the halide anions, supporting a BOPPY cation radical mechanism hypothesis. Such CuCl_2_ or CuBr_2_ mediated halogenations are well-precedented as ONSH reactions, a class of transformations extensively developed for direct C-H functionalization of electron-deficient heteroaromatic systems.^15^

**Scheme 2:**
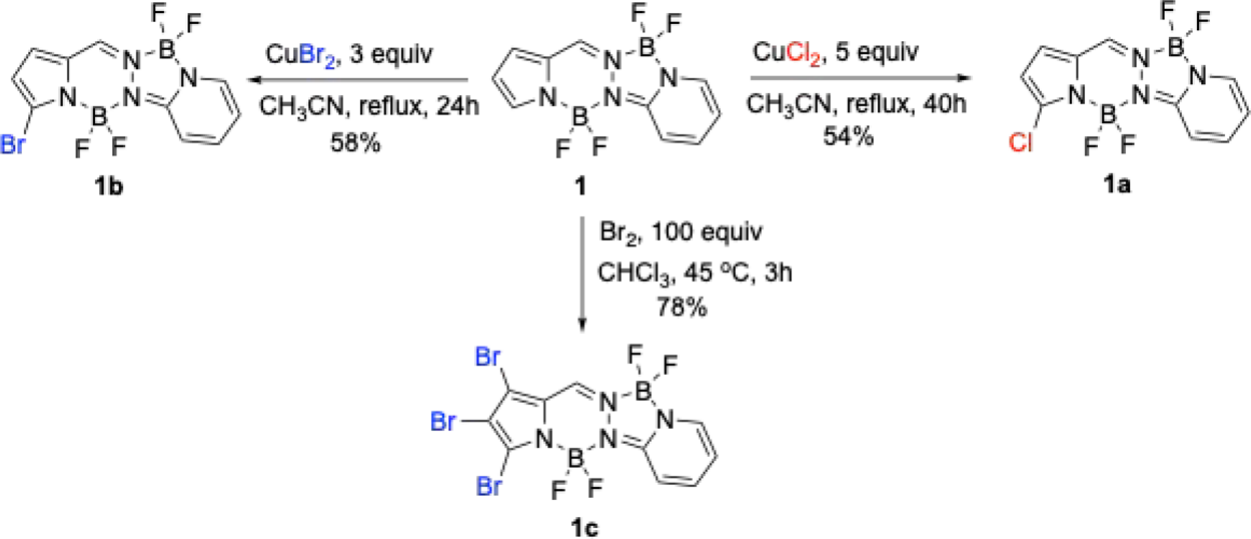
Regioselective Halogenations of BOPPY **1**

Using a large excess of CuBr_2_ and extending the reaction time led to the formation of trace amounts of the tribrominated product **1c**, along with other minor brominated derivatives. To obtain the tribrominated product **1c** in high yield, the more reactive brominating agent, liquid Br_2_ in chloroform,^16^ was used (Scheme 2). In the presence of a large excess of Br_2_ (100 equiv) BOPPY **1c** was the main product isolated in 78% yield. ^1^H NMR spectroscopy of **1c** revealed the disappearance of all pyrrolic protons formerly at 6.60, 7.09 and 7.82 ppm. Bromination of the pyridine ring was not observed under these conditions. The stepwise disappearance of the pyrrolic protons provides clear spectroscopic evidence for preferential α-functionalization, followed by modification at the less reactive β-sites.

The α-pyrrolic halogenation regioselectivity was further confirmed by X-ray crystallography. Crystals of **1a**, **1b** and **1c** were grown from slow diffusion of hexane into dichloromethane; their X-ray structures are shown in Figure 1 and are deposited as CCDC 2486948–2486950. In BOPPY **1a**, the 16-atom BOPPY core is fairly planar, with a mean deviation of 0.08 Å. The two boron atoms have the largest deviations, averaging 0.21 Å on the same side of the best plane. The C-Cl distance is 1.707(2) Å. The structure of BOPPY **1b** is very similar to that of **1a**, with mean core deviation 0.08 Å and the boron atoms averaging 0.22 Å out of plane on the same side. The C-Br distance is 1.862(5) Å. BOPPY **1c** is slightly less planar than **1a** and **1b**, with a slightly bowed BOPPY core having mean deviation 0.10 Å. As before, both boron atoms lie on the same side of this plane, with an average deviation of 0.23 Å. The C-Br distance at the 3-position is slightly shorter, 1.854(2) Å, relative to the C-Br distances at the β-positions 1 and 2, which are equal, 1.863(2) Å. DFT calculations of the optimized geometries of the halogenated BOPPYs confirm the near planarity of the BOPPY core observed by X-ray crystallography. Furthermore, in agreement with the experimental findings for the structure of **1c**, the 3C-Br bond is shorter than the C-Br bonds at positions 1 and 2, which are equal in length. Figure 2 compares the molecular electrostatic potential (MESP) maps for BOPPYs **1** and **1c**. The red regions indicate a more negative potential, the blue regions a more positive potential, and the green-yellow regions, an intermediate potential. The numerical values of MESPs at the boron nuclei are also given. As can be seen in Figure 2, for BOPPY **1** the two BF_2_ groups have significantly different electron density, with the boron atom closest to the pyrrole ring having the most negative potential. The same trend is observed in the case of BOPPY **1c** although the tribromo substitution results in less negative potentials for both the boron nuclei.

**Figure 1:**
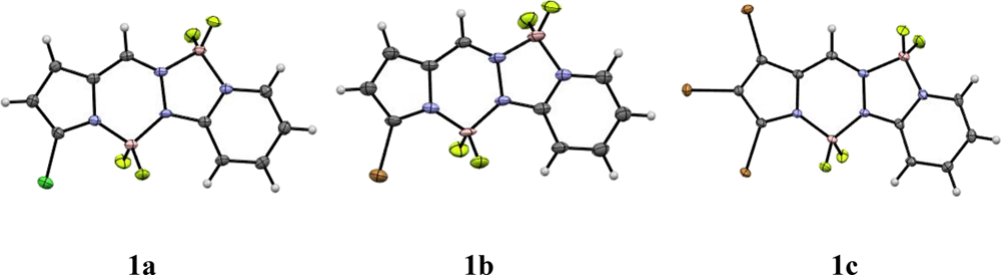
X-ray crystal structures of halogenated BOPPYs with anisotropic displacement parameters shown at the 50% probability level.

**Figure 2:**
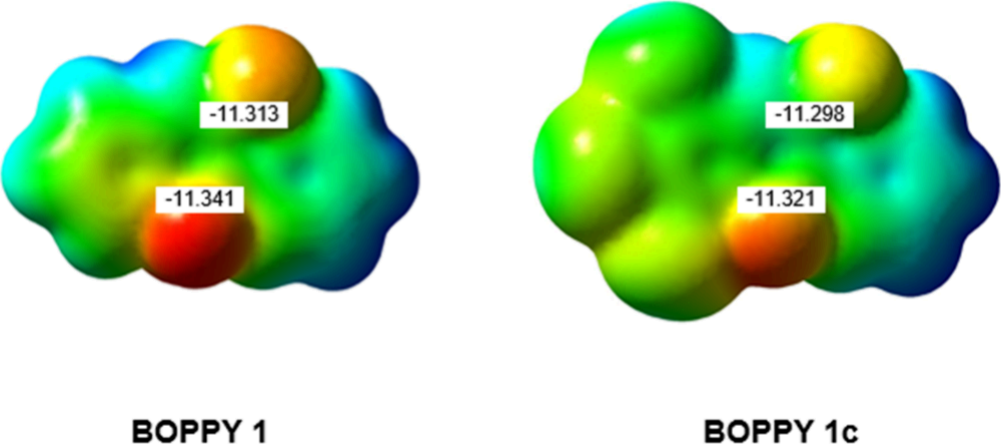
Molecular electrostatic potential (MESP) maps for BOPPYs **1** and **1c** and MESPs (in a.u.) at their B nuclei. Calculated at the MN15/6-311++G­(d,p) level in acetonitrile. The red regions indicate a more negative potential, while the blue regions indicate a more positive potential.

The mono- and trihalogenated BOPPYs **1a**, **1b** and **1c** are highly desirable synthetic precursors for the preparation of functionalized derivatives, for example via Suzuki and Stille type cross-coupling reactions and nucleophilic substitutions, as described below. Furthermore, the tribromo BOPPY **1c** allowed the investigation of the regioselectivity of these reactions.

### 2.2 Pd-Cross-Coupling Reactions

Halogenated BODIPY derivatives are widely recognized as versatile precursors for postsynthetic functionalization.^13,16–20^ In the present study, the brominated BOPPY derivatives proved to be excellent substrates for transition metal-catalyzed cross-couplings, thereby enabling further diversification of the chromophore framework. The Suzuki-Miyaura coupling was employed to introduce a variety of aryl substituents at the α-brominated position of BOPPY **1b** (Scheme 3). Using Pd­(PPh_3_)_4_ as the catalyst and Na_2_CO_3_ as the base, the α-bromo-BOPPYs underwent efficient C–C bond formation with diverse arylboronic acids. Initial trials without a phase-transfer catalyst gave low yields of the corresponding products. However, addition of tetrabutylammonium bromide (TBAB) markedly improved the reaction yields, underscoring its role in facilitating the transmetalation step. TBAB enhances the base solubility in toluene, stabilizes the palladium intermediates, and promotes transfer of the ionic species across phases, collectively boosting catalytic turnover and dramatically improving the overall yield of the reaction.^21,22^

**Scheme 3:**
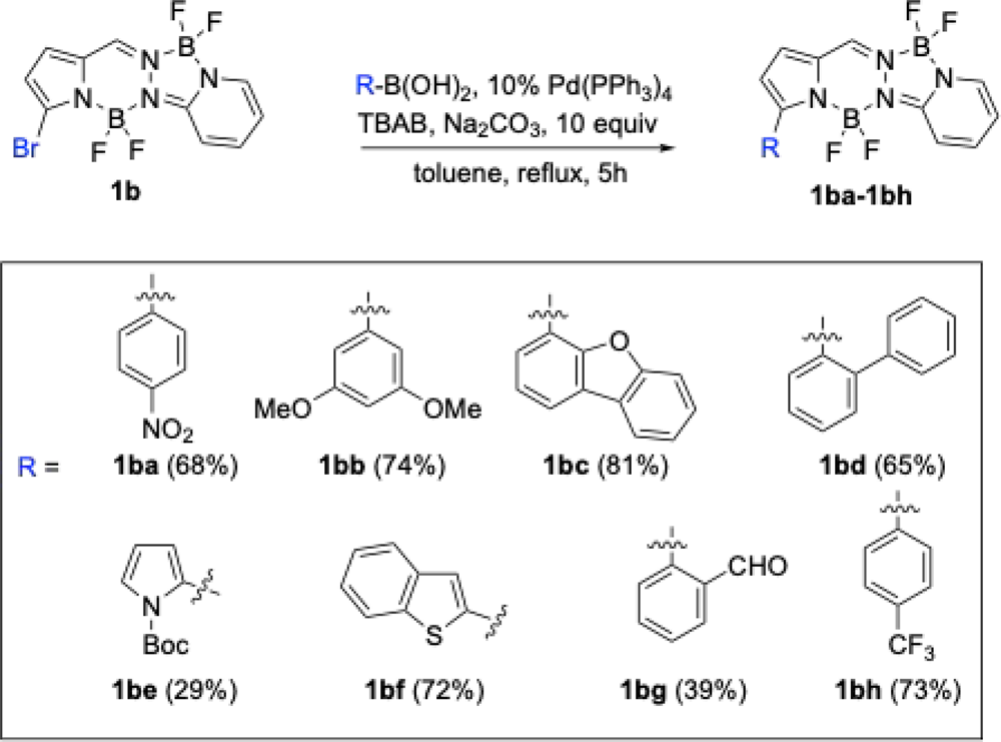
Pd-Catalyzed Suzuki Cross-Coupling Reactions on BOPPY **1b**

Electron-rich substrates, such as 3,5-dimethoxyphenylboronic acid, readily reacted with BOPPY **1b** at the α-bromo position to afford BOPPY **1bb** in 74% yield. Similar reactivity was observed using electron-deficient boronic acids, producing BOPPYs **1ba** (from 4-nitrophenylboronic acid) and **1bh** (from 4-trifluoromethylphenylboronic acid) in 68 and 73% yields respectively. Heteroaryl boronic acids were also employed and the corresponding functionalized BOPPYs were also obtained in good yields. Thus, dibenzo­[*b,d*]­furan-4-ylboronic acid gave BOPPY **1bc** in 81% yield, while benzo­[b]­thiophen-2-ylboronic acid afforded BOPPY **1bf** in 72% yield. The biaryl substrate [1,1′-biphenyl]-2-ylboronic acid afforded BOPPY **1bd** in slightly lower yield (65%), likely due to steric hindrance imposed by the *ortho*-boronic acid substituent in the transmetalation step of the catalytic cycle.

Substrates bearing sensitive functional groups proved less efficient under the optimized conditions described above. For example, the coupling of 1-(*tert*-butoxycarbonyl)-1*H*-pyrrol-2-yl)­boronic acid with **1b** gave BOPPY **1be** in only 29% yield, while 2-formylphenylboronic acid delivered BOPPY **1bg** in 39% yield. In both cases, several byproduct spots were observed on TLC, indicating various side reactions, likely arising from the instability of the functional groups under the reaction conditions. BOPPY **1be** was further treated with excess TFA in dichloromethane at room temperature, to remove the Boc protecting group. This deprotection was expected to produce the corresponding NH-free pyrrolic substituent, which could potentially show large bathochromic shifts in its absorption and emission profiles relative to the starting material, along with increased fluorescence, through enhanced rigidity via intramolecular hydrogen bonding interactions with the BF_2_ unit, as we have previously observed.^23,24^ Surprisingly, the only product isolated in the reaction was BOPPY **1be′**, in which the oxygen atom of the Boc group displaced one fluorine atom on the nearby boron center, before the decarboxylation step occurred (Scheme 4). This observed reactivity highlights the strong affinity of oxygen donors for the boron center, leading to substitution at the BF_2_ unit rather than Boc removal prior to the decarboxylation step. We have previously investigated boron functionalization of BODIPY compounds with amino acid derivatives under mild conditions, leading to the displacement of one of the boron fluorines by the carboxylate group, catalyzed by BCl_3_.^25,26^ In the case of BOPPY **1be** in the presence of TFA, cleavage of the Boc group presumably leads to release of isobutene and a free carboxylate group which readily attacks the nearby boron, substituting one of the fluorines before decarboxylation can occur at temperatures up to 55 °C.

**Scheme 4:**
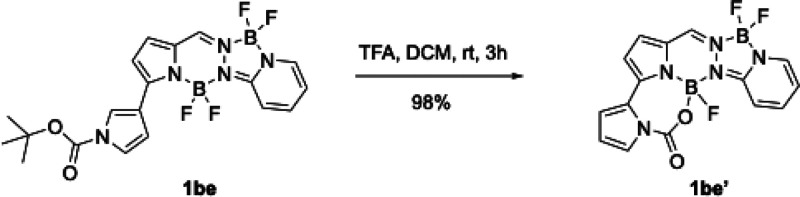
Boc-Deprotection of BOPPY **1be** to Give **1be’**

The Stille coupling of α-bromo-BOPPY **1b** in the presence of 2-(tributylstannyl)­thiophene under Pd­(PPh_3_)_4_ catalysis, produced BOPPY **1bi** in excellent (91%) yield (Scheme 5). The absence of base and the relatively milder conditions used in this coupling reaction led to low side-product formation and higher yield of the desired product. Interestingly, under similar conditions, tribromo-BOPPY **1c** regioselectively produced BOPPY **1ca** in excellent (96%) yield (Scheme 5). This observed regioselectivity might be due to the slightly longer, and therefore weaker, C1–Br bond compared to the C3-Br bond, as shown by both X-ray crystallography and DFT calculations. In addition, we believe that upon oxidative addition of Pd(0), the large triphenylphosphine groups cause steric hindrance with the nearby BF_2_ group, disfavoring oxidative addition at the C3-Br bond compared to the C1–Br bond. We have previous observed similar regioselectivity on a perhalogenated BODIPY under similar Stille reaction conditions.^19^ Furthermore, DFT calculations in toluene showed that BOPPY **1ca** is the most stable product among all possible regioisomers, by approximately 6 kcal/mol (5.8 kcal/mol relative to the 2-regioisomer and 6.5 kcal/mol relative to the 3-regioisomer).

**Scheme 5:**
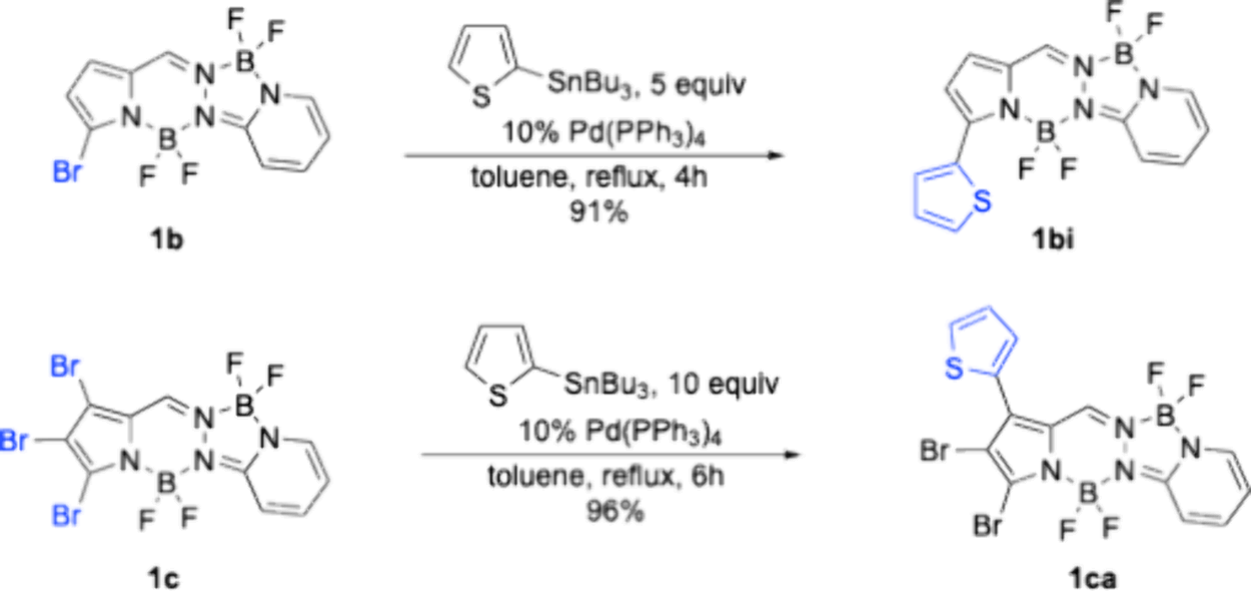
Pd-Catalyzed Stille Coupling Reactions of Brominated BOPPYs **1b** and **1c**

The structures of all the BOPPY coupling products were fully characterized by ^1^H, ^13^C and ^11^B NMR spectroscopy, high-resolution mass spectrometry (HRMS), and X-ray crystallography. The ^1^H and ^13^C NMR spectra confirmed the appearance of new aromatic or heteroaryl signals consistent with successful substitution, while the ^11^B NMR spectra clearly showed two distinct triplets at around 1 and 3 ppm, attributed to the boron on the six and five membered rings, respectively. HRMS data matched the calculated values within 3 ppm, further validating the molecular formulas. In addition, single crystals suitable for X-ray diffraction were obtained for representative derivatives, allowing unambiguous confirmation of their connectivity and substitution patterns. The crystals were grown from slow diffusion of hexane into dichloromethane and the X-ray structures obtained are shown in Figure 3. The nine structures **1ba** through **1ca** are deposited as CCDC 2487133–2487141. The BOPPY core of **1ba** is nearly planar, with mean deviation 0.03 Å and the boron atoms not deviating significantly from this plane. The phenyl plane of the nitrophenyl substituent makes a dihedral angle of 40.0° with the BOPPY plane. The BOPPY core in **1bb** is also fairly planar, with mean deviation of 0.04 Å, and the phenyl ring makes a dihedral angle of 53.5° with it. The structure of BOPPY **1bc** has four independent molecules with similar shapes. The average deviation from the BOPPY plane is 0.07 Å (mean of 4), with the boron atoms out of plane to the same side. The BOPPY/dibenzofuran dihedral angle (mean of 4) is 55.6°. BOPPY **1bd** has two independent molecules. Its core has a slightly bowed shape, with mean deviation 0.07 Å. The dihedral angle between the core and the attached phenyl ring is 64.7° (average of 2) and the dihedral angle between the two biphenyl rings 51.0° (average of 2). BOPPY **1be’** has two independent molecules. The coordination of O rather than F to boron does not affect the planarity of the BOPPY core, as the mean deviation from planarity is 0.03 Å. The pyrrole carboxylate plane forms a dihedral angle of 25.0° (average of 2) with the core. Compound **1be’** is an example of a chiral fluorophore with an asymmetric boron atom. It has two mirror-image molecules in the asymmetric unit, and therefore is a kryptoracemate,^27^ crystallizing in a Sohncke space group, having no symmetry elements which change the hand. Such compounds are relatively rare, occurring for approximately 1% of racemates. BOPPY **1bf** also has two independent molecules, and the BOPPY core is slightly less planar, with mean deviation of 0.06 Å. The benzothiophene plane makes a dihedral angle of 36.4° (average of 2) with the core. The structure of BOPPY **1bh** has three independent molecules. The BOPPY core is slightly bowed, with a mean deviation of 0.09 Å, and the mean core/phenyl dihedral angle is 42.1°. The BOPPY core of **1bi** is distorted from planarity similar to the tribromo compound, with a mean deviation of 0.09 Å and both boron atoms out of plane on the same side. The core/thiophene dihedral angle is 24.1°. BOPPY **1ca** has two independent molecules, and the core has a slightly bowed shape with mean deviation of 0.08 Å. The core plane makes a dihedral angle of 41.1° with the thiophene plane. The C-Br distances are equal, with a mean value of 1.862 Å.

**Figure 3:**
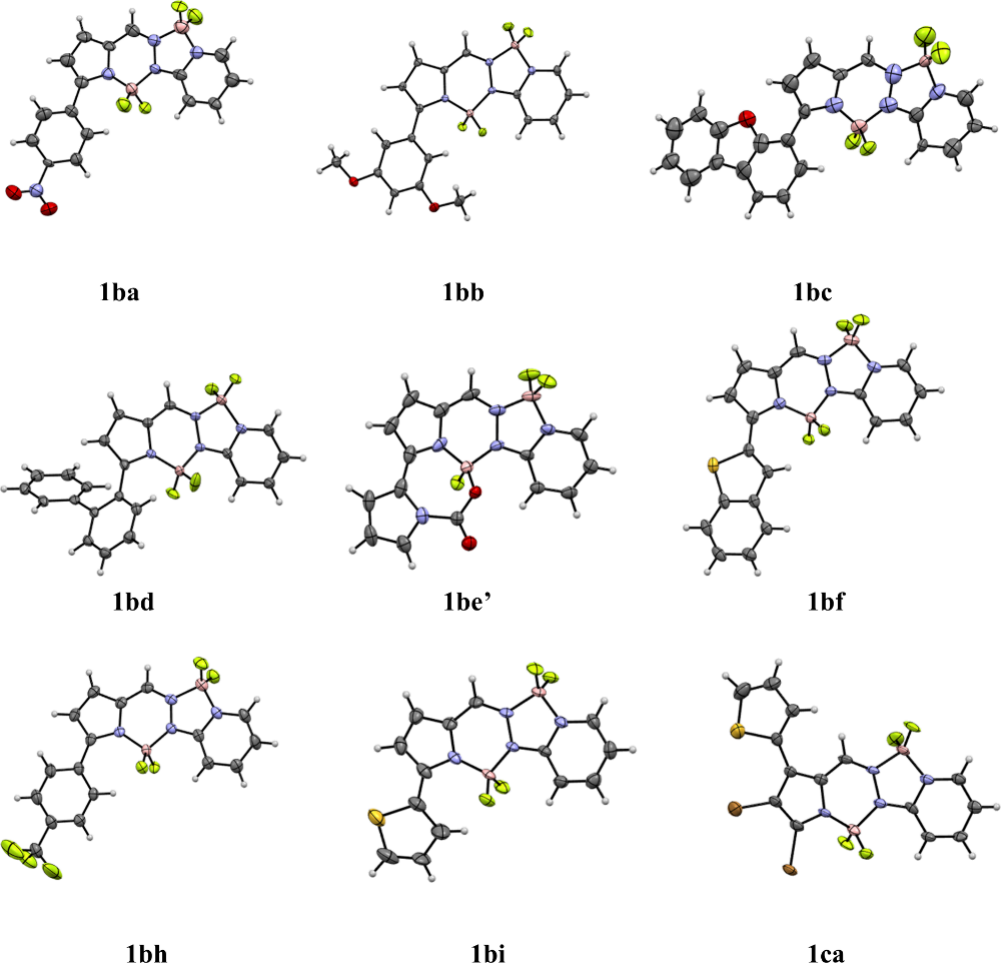
X-ray crystal structures of BOPPY derivatives obtained from cross-coupling coupling reactions, with anisotropic displacement parameters shown at the 50% probability level.

### 2.3 Nucleophilic Aromatic Substitution

To further diversify the BOPPY scaffold, nucleophilic aromatic substitution (S_N_Ar) reactions were explored using the highly reactive 4-methoxythiophenol as the nucleophile.^24^ The reaction of monobromo-BOPPY **1b** under reflux conditions in chloroform or toluene gave the desired product BOPPY 1bj in low yield (<20%), along with recovered starting material. On the other hand, refluxing in *o*-xylene for 24 h afforded BOPPY **1bj** in 38% yield with only traces of starting material recovered (Scheme 6). The modest yield of the reaction is attributed to side reactions and decomposition under the prolonged high-temperature conditions.

**Scheme 6:**
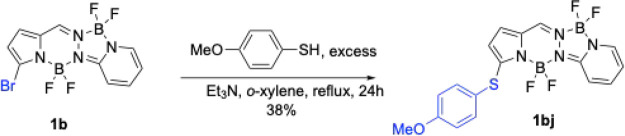
Nucleophilic Aromatic Substitution Reaction of BOPPY **1b**

The more electron-deficient tribromo-BOPPY **1c** showed higher reactivity compared with **1b** under milder conditions (Scheme 7). In the presence of an excess of 4-methoxythiophenol, substitution occurred first at the most reactive C3 followed by the C1 position. While at room temperature no reaction occurred, heating to 60 °C in chloroform led to sequential and regioselective substitution. Careful monitoring by TLC revealed the initial formation of the monosubstituted BOPPY **1cb**, followed by the more polar disubstituted BOPPY **1cc**. This regioselectivity is consistent with the calculated least negative MESP at C3 position, followed by the C1 site (see the Supporting Information, Figure S67). The data suggests that while both the C1 and C3 positions are electronically activated toward nucleophilic substitution, their comparable reactivity under strong nucleophilic conditions promotes the formation of both the mono- and disubstituted derivatives **1cb** and **1cc**.

**Scheme 7:**
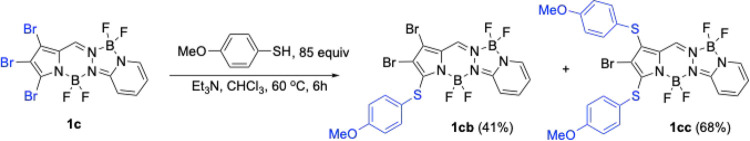
Nucleophilic Aromatic Substitution Reactions of BOPPY **1c**

Interestingly, while the monosubstitution of BOPPY **1c** under nucleophilic reaction conditions occurred preferentially at the α-pyrrolic position, the Stille coupling of **1c** led to regioselective substitution at the C1 position (see above).

The substitution products were fully characterized by ^1^H, ^13^C and ^11^B NMR spectroscopy, HRMS, and by single-crystal X-ray diffraction. The X-ray analysis provided unambiguous confirmation of their structures and substitution sites. The crystals were grown from slow diffusion of hexane into dichloromethane and the X-ray structures obtained are shown in Figure 4. The three structures are deposited as CCDC 2487291–2487293. The BOPPY core of **1bj** is slightly bowed, with a mean deviation of 0.11 Å and both boron atoms on the same side of the plane. The phenyl group of the substituent is nearly orthogonal to the core plane, with a dihedral angle of 86.8°, and the N-C-S-C torsion angle is 179.70(17)°. The core of **1cb** is fairly planar, with a mean deviation of 0.04 Å. The phenyl plane of the thioaryl substituent makes a dihedral angle of 75.9° with it, and the N-C-S-C torsion angle is −108.8(4)°. The C-Br distances are equal, with a mean value of 1.868 Å. The BOPPY core of **1cc** has a mean deviation of 0.07 Å, with the boron atoms showing the largest deviations, average 0.22 Å on the same side of the plane. The thioaryl substituent at the 3 position is nearly orthogonal to the core, with dihedral angle of 89.0° and the C-C-S-C torsion angle is −113.0°. The thioaryl substituent at the 1 position is also nearly orthogonal to the core, with dihedral angle 88.6° and N-C-S-C torsion angle 123.9°. The C-Br distance is 1.863 Å.

**Figure 4:**
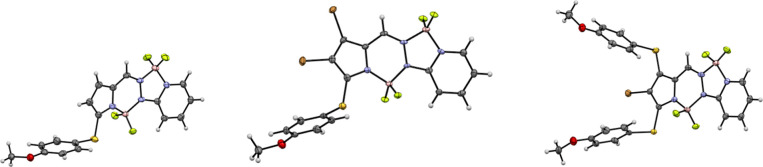
X-ray crystal structures of BOPPY derivatives obtained from S_N_Ar reactions, with anisotropic displacement parameters shown at the 50% probability level.

### 2.4 Photophysical Properties

The spectroscopic properties of the BOPPY derivatives were investigated in toluene, dichloromethane and acetonitrile; the results are summarized in Table 1, Figure 5, and in the Supporting Information, Table S1 and Figures S1–S12. In addition, we performed computational modeling of the ground and first excited states of the synthesized compounds using TD-DFT/MN15/6-311++G­(d,p) calculations. The results from these studies are summarized in Table S2 of the Supporting Information. The frontier molecular orbitals for BOPPYs **1ba**, **1bb**, **1bc** and **1bh** are given in Figure 6, and those for **1c**, **1ca**, **1cb** and **1cc** in Figure 7. The frontier molecular orbitals for all other synthesized compounds are given in Figure S68 of the Supporting Information.

**Figure 5:**
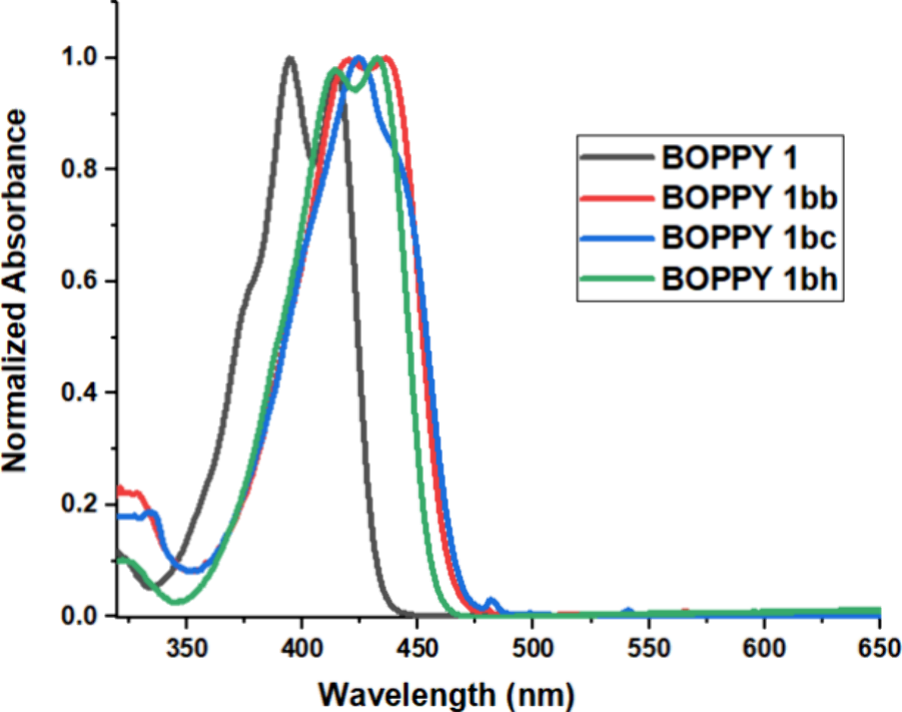
Normalized absorption spectra for BOPPYs **1**, **1bb**, **1bc** and **1bh** at room temperature in dichloromethane.

**Figure 6:**
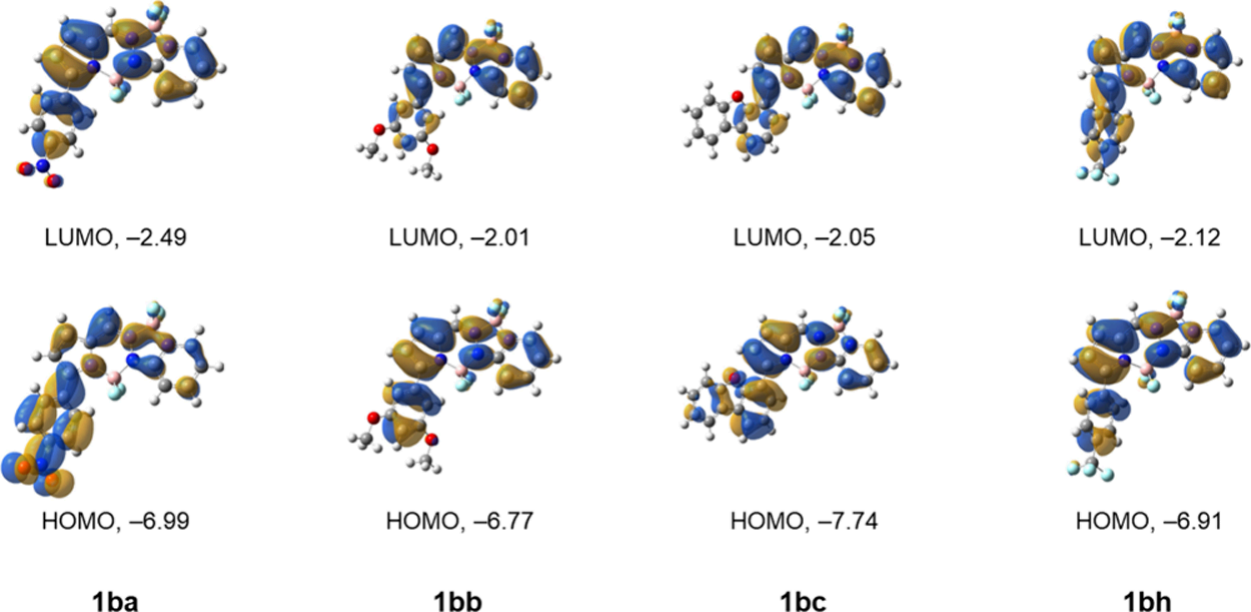
Frontier MO diagram for BOPPYs **1ba**, **1bb**, **1bc** and **1bh**. Energies in eV. Calculated at the MN15/6-311++G­(d,p) level in dichloromethane. The frontier MOs of all compounds in the series can be found in the Supporting Information, Figure S68.

**Figure 7:**
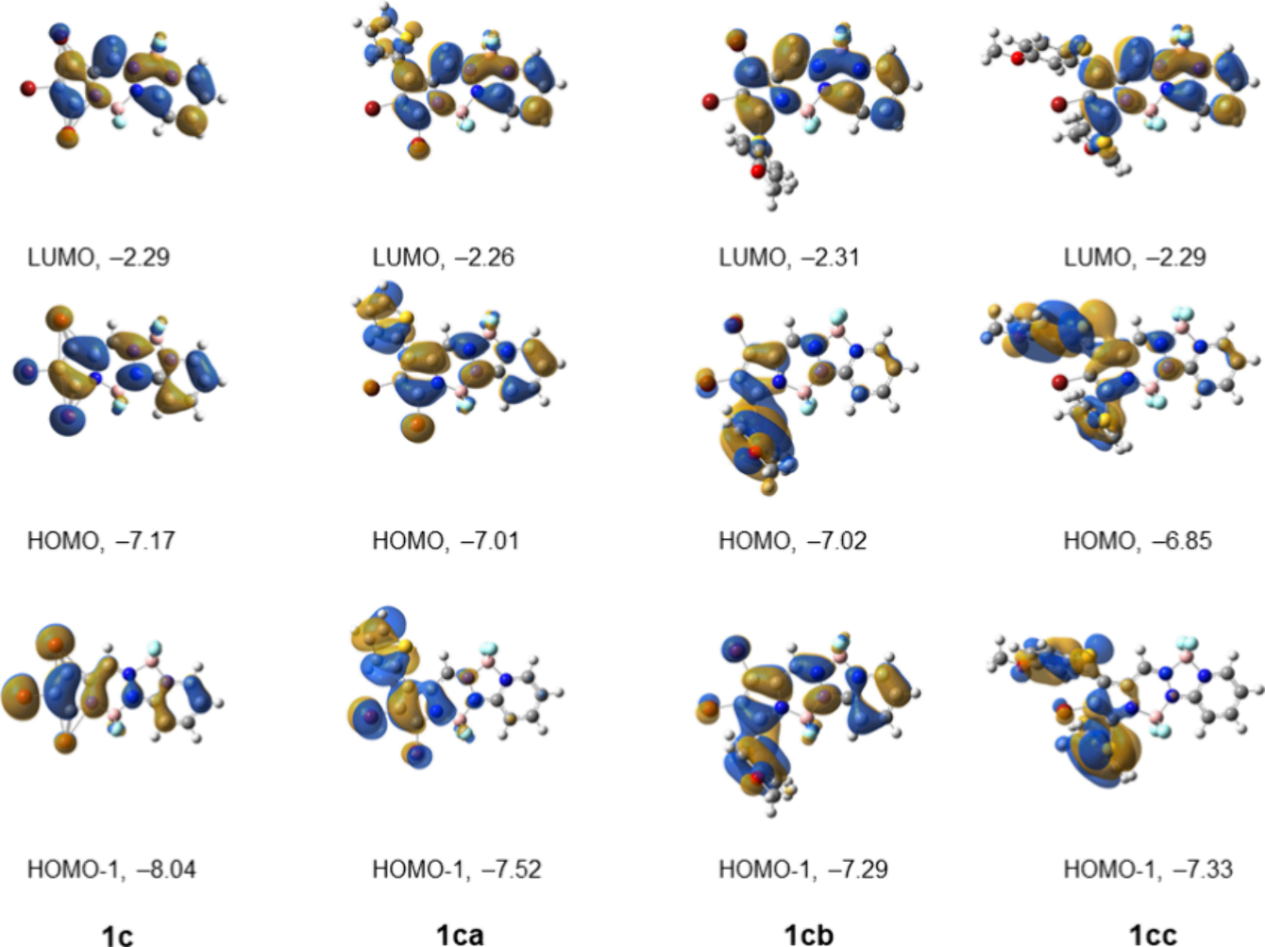
Frontier MO diagram for BOPPY **1c**, **1ca**, **1cb**, **1cc**. Energies in eV. Calculated at the TD-DFT MN15/6-311++G­(d,p) level in dichloromethane. The frontier MOs of all compounds in the series can be found in the Supporting Information, Figure S68.

**Table 1:** Photophysical Properties of BOPPYs in Dichloromethane at Room Temperature

	**λ**_**abs**_^**max**^ **/nm** **(log ε**_**max**_ **)**	λ em (nm)	**Stokes** **Shift/cm** ^**–1**^	**Φ**_**F**_ ^a^
**1**	396(4.52), 416(4.51)	433, 457	2400	0.79^b^
**1a**	400(4.43), 421(4.46)	437, 459	900	0.72
**1b**	402(4.36), 422(4.39)	438, 463	900	0.74
**1c**	406(4.58), 428(4.66)	442, 469	700	0.57
**1ba**	428(4.43), 447(4.36)	480b	2500	0.40
**1bb**	422(4.55), 439(4.55)	466, 492	1300	0.95
**1bc**	425(4.57), 441(4.49)	469, 499	2200	0.98
**1bd**	414(4.47), 430(4.49)	466	1800	0.85
**1be’**	448(4.40), 467(4.48)	497	1300	0.90
**1bf**	440(4.33), 464(4.35)	488, 515s	1100	1.00
**1bg**	405(4.21), 419(4.24)	457	2000	0.76
**1bh**	414(4.53), 433(4.54)	457, 483s	1200	0.89
**1bi**	403(4.19), 423(4.27)	438, 466	700	0.82
**1bj**	429(4.58), 451(4.54)	440, 470	500	0.21
**1ca**	420(4.41), 436(4.48)	477	2000	0.09
**1cb**	414s, 434(4.45)	-	-	0.00
**1cc**	417s, 438(4.35)	-	-	0.00

a Fluorescence quantum yields (Φf) determined using BOPPY **1** in dichloromethane (Φ = 0.79) as standard.

b Reported fluorescence quantum yield in dichloromethane is Φf = 0.79.

As expected, all compounds exhibit dual absorption bands in the 396–467 nm range in dichloromethane, at 393–454 nm in acetonitrile, and at 399–474 nm in toluene, corresponding to the π-π* transitions of the conjugated chromophore, as confirmed by the TD-DFT calculations. The dual absorption bands are likely due to vibronic transitions as previously identified^10,24^ and associated with the significant change in the N-N bond upon excitation, as discussed below.

Emission maxima were typically observed between 433 and 515 nm, with Stokes shifts ranging from 500 to 2700 cm^–1^. The introduction of one halogen atom (Cl, Br) at the α-pyrrolic position shows small bathochromic shifts in the absorption and emission wavelengths (4–6 nm), consistent with their similar calculated band gaps (Supporting Information, Table S2 and Figure S68). The tribromo-BOPPY **1c** showed more pronounced bathochromic shifts, along with a decrease in Stokes shift and decreased fluorescence, due to the heavy atom effect of the three bromines. Analysis of the geometry changes for all substituted BOPPYs upon excitation reveals that the structure of the BOPPY core remains relatively planar. In agreement with our previous findings for BOPYPY^24^ and previous studies of BOPPY,^10^ the major change is in the N-N bond, which is systematically 0.03–0.05 Å shorter in the excited states (Supporting Information, Table S2). This is consistent with the more N-N antibonding character of the HOMO compared to the LUMO (Figure 6).

The introduction of aryl substituents to BOPPYs **1b** and **1c** produced bathochromic shifts in the range 7–52 nm. The largest shifts were observed for BOPPY **1be’** and the lowest for **1bf**. This is consistent with the calculated smaller HOMO–LUMO gaps for all substituted BOPPYs compared with **1** (Supporting Information, Table S2 and Figure S68). Electron-donating aryl groups, such as in **1bb** and **1bc**, slightly lower LUMO (less than 0.1 eV) and significantly destabilize the HOMO (∼0.7 eV), thus resulting in smaller HOMO–LUMO gaps. Analysis of the MOs for **1bb** and **1bc** (Figure 6) demonstrates that the electron density in the HOMO and LUMO remains localized on the BOPPY core, which is also consistent with the observed very high fluorescent quantum yields for these molecules (Φ_f_ = 0.95 and 0.98, respectively). On the other hand, the electron-withdrawing character of substituents in BOPPYs **1ba** and **1bh** can be clearly seen in the delocalization of the MOs for these compounds (Figure 6). In the case of **1ba**, the effect is mainly on the LUMO, where a significant part of the electron density is localized on the nitrophenyl substituent. Therefore, charge-transfer will be observed upon excitation, which is consistent with the observed significantly reduced fluorescence quantum yield (Φ_f_ = 0.40). For **1bh**, significant delocalization to the electron-withdrawing trifluoromethylphenyl substituent is also observed; however, in this case it is for both HOMO and LUMO. As a result, the fluorescence quantum yield remains high (Φ_f_ = 0.89). The highest fluorescence quantum yield observed (Φ_f_ = 1.0) was for BOPPY **1bf** bearing a benzo­[b]­thienyl group directly attached to the α-pyrrolic position. On the other hand, BOPPY **1bi** showed slightly lower fluorescence (Φ_f_ = 0.82) compared with **1bf** likely due to the greater rotational freedom of the smaller thienyl group, leading to increased energy loss due to nonradiative decay to the ground state.^17^

The S_N_Ar products bearing a sulfur atom directly attached to the BOPPY chromophore, as in BOPPYs **1bj**, **1cb and 1cc**, showed significantly decreased fluorescence quantum yields. For BOPPY **1bj** (Φ_f_ = 0.21), this is likely due to the greater vibrational and rotational freedom of the 4-methoxythiophenyl group, resulting in higher probability for nonradiative energy loss. This effect is also true for **1cb** and **1cc**; however, in these cases, there are additional electronic density reasons for the observed fluorescence quenching. The MO diagrams for BOPPYs **1c**, **1ca**, **1cb**, and **1cc** are given in Figure 7. For all these compounds the dominant transition is HOMO → LUMO. For **1cb**, there is a significant contribution from the transition HOMO–1 → LUMO, because of the closer proximity in energy of HOMO–1 to HOMO, compared with the rest of the molecules. For all the HOMO and HOMO-1 orbitals of the molecules in Figure 7, there is electron density delocalized over the substituents. In the case of the **1ca** HOMO, the electron density delocalizes onto the thiophene group; however a significant amount still remains on the BOPPY core, resulting in a small, although not zero, quantum yield (Φ_f_ = 0.09). In **1cb** and **1cc** HOMO, there is even less electron density on the BOPPY core. Thus, the HOMO → LUMO transition is quenched due to charge transfer. Moreover, the performed TD-DFT calculations show that the second and third excited states for **1ca**, **1cb** and **1cc** are closer in energy to S_1_ compared to the other members of the series. S_2_ is 0.6 eV above S_1_ for **1ca**, 0.3 eV for **1cb**, and just 0.2 eV for **1cc**. For BOPPY **1cc**, S_3_ is just 0.2 eV above S_2_. These small energy differences suggest increased probabilities for internal conversion. Therefore, we hypothesize that the observed fluorescence quenching in BOPPYs **1ca**, **1cb**, and **1cc** is due to a combination of internal conversion and charge-transfer. In addition, the heavy atom effect also plays a role in the observed fluorescence quenching due to enhancement of intersystem crossing from the excited singlet to the triplet state.

The solvent effects observed were consistent with the polarity trend of the solvents used (Supporting Information, Table S1). In general, the fluorescent quantum yield decreased as the polarity of the solvent increased, which is consistent with previous observations of BODIPY dyes^28,29^ and it can be due to multiple factors, including external conversion, stabilization of charge transfer states, extended conjugation, solute–solvent hydrogen bonding, and specific solvation. The majority of synthesized BOPPYs also demonstrated small blue shifts in polar solvents. However, **1bi**, **1cb** and **1cc** demonstrated red shifts. This points toward a complex nature and the role of multiple factors in the solvatochromic effect in the studied BOPPYs.

## 3 Conclusions

We report the synthesis of a new series of functionalized BOPPYs derived from halogenation of the pyrrolic positions, followed by Pd(0)-mediated cross-coupling Suzuki and Stille reactions, and from nucleophilic substitutions using 4-methoxythiophenol. The nucleophilic substitution of tribrominated BOPPY **1c** occurred preferentially at C3 followed by C1, while the cross-coupling reactions occurred first at C1 followed by C3, due to a combination of electronic and steric factors. The structures of the functionalized BOPPYs were characterized by NMR spectroscopy, HRMS and X-ray crystallography. In the X-ray structures of 15 functionalized BOPPYs presented herein (24 counting Z’>1 structures), the BOPPY cores are nearly planar, and the observed slight deviations from planarity tend to be bowed with the two boron atoms out of plane on the same side and B-N-N-B torsion angle magnitudes in the range 155.9–179.9°. The planes of substituents form dihedral angles that range from small (24.1°) to nearly orthogonal (89.0°).

The introduction of various groups on halogenated BOPPYs via Pd(0)-catalyzed Suzuki and Stille cross-coupling reactions, and nucleophilic substitutions, produced functionalized BOPPYs featuring bathochromic shifts in both the absorption and emission wavelengths, of up to 52 nm, and Stokes shifts in the 500 to 2500 cm^–1^ range. Electron-donating aryl substituents typically slightly lower the LUMO and significantly destabilize the HOMO, resulting in smaller HOMO–LUMO gaps and bathochromic shifts. The electron-withdrawing substituents lower both the HOMO and LUMO but the effect on the LUMO is predominant, again resulting in smaller HOMO–LUMO gaps and bathochromic shifts. The fluorescence quantum yields of the functionalized BOPPYs varied from 0 to 1; BOPPY **1bf** bearing a benzo­[b]­thienyl group attached to the α-pyrrolic position showed the highest fluorescence quantum yield, while **1cb** and **1cc** were nonemissive, due to a combination of charge transfer and internal conversion nonradiative processes. Our findings suggest that multiple substitutions at the BOPPY periphery might disrupt conjugation, increase vibrational and rotational freedom, and create multiple excited states that are very close in energy, causing fluorescence quenching.

## 4 Experimental Section

### 4.1 General

All reagents and solvents were obtained from commercial vendors and used without further purification unless specifically stated. Reactions were carried out in oven-dried glassware and monitored using plastic-backed thin-layer chromatography (TLC) plates. Visualization of TLC plates was performed under UV light at 254/365 nm. Unless otherwise noted, equivalents are reported relative to the limiting BOPPY precursor (1.00 equiv) and reaction concentrations are reported as molarity with respect to the limiting precursor. Compound purification was achieved either via silica gel column chromatography (60 Å, 40–63 μm) or using silica-backed preparative TLC plates, both from Sorbtech.

NMR spectra were recorded on a Bruker spectrometer operating at the following frequencies: 400 MHz for ^1^H, 126 MHz for ^13^C, and 128 MHz for ^11^B. Chemical shifts (δ) are reported in parts per million (ppm) relative to standard references: CDCl_3_ (7.26 ppm for ^1^H, 77.0 ppm for ^13^C), acetone-d_6_ (2.05 ppm for ^1^H) and BF_3_·OEt_2_ in CDCl_3_ (0.00 ppm for ^11^B). Coupling constants (J) are given in hertz (Hz) and signal multiplicities are designated as follows: s (singlet), d (doublet), t (triplet), q (quartet), dd (doublet of doublets), td (triplet of doublets), and m (multiplet). High-resolution mass spectrometry (HRMS) data were collected at the LSU Mass Spectrometry Facility (MSF) using an the Synapt XS ESI-Q-IM-TOF instrument supported by NIH (Grant 1S10OD030429–01A1). The spectroscopic data obtained for BOPPY **1** agrees with that previously reported.^10^

### 4.2 Synthesis and Characterization

#### 4.2.1 Regioselective Chlorination of BOPPY **1**

A mixture of BOPPY **1** (18 mg, 0.064 mmol, 1.00 equiv) and CuCl_2_·2H_2_O (56 mg, 0.330 mmol, 5.00 equiv) was dissolved in anhydrous CH_3_CN (15 mL) to give a reaction concentration of 0.0043 M with respect to BOPPY 1. The reaction mixture was heated at reflux in an oil bath with stirring for 40 h. After cooling to room temperature, the mixture was diluted with water (30 mL) and extracted with CH_2_Cl_2_ (3 × 30 mL). The combined organic extracts were dried over anhydrous Na_2_SO_4_, filtered and concentrated under reduced pressure. The crude residue was purified by silica gel column chromatography (CH_2_Cl_2_/hexane, 1:1 v/v) to afford BOPPY **1a** (11 mg, 54%) as a yellow solid. Mp 265–267 °C. ^1^H NMR (400 MHz, CDCl_3_) δ 8.00 – 7.90 (m, 2H), 7.71 (s, 1H), 7.59 (d, *J* = 8.9 Hz, 1H), 7.08 – 6.98 (m, 2H), 6.47 (d, *J* = 4.0 Hz, 1H). ^13^C­{1H} NMR (126 MHz, CDCl_3_) δ 152.5, 144.1, 136.5, 133.1, 131.5, 123.6, 115.7, 115.6, 111.8. ^11^B NMR (128 MHz, CDCl_3_) δ 2.97 (t, *J* = 24.7 Hz), 0.82 (t, *J* = 28.8 Hz). HRMS (ESI-TOF) *m*/*z* [(M+H)-BF_2_]^+^ calcd. for C_10_H_9_BClF_2_N_4_, 269.0577, found 269.0578.

#### 4.2.2 Regioselective Bromination of BOPPY **1**

A mixture of BOPPY 1 (31 mg, 0.110 mmol, 1.00 equiv) and CuBr_2_ (76 mg, 0.341 mmol, 3.00 equiv) was dissolved in anhydrous CH_3_CN (15 mL) to give a reaction concentration of 0.0073 M with respect to BOPPY **1**. The reaction mixture was heated at reflux in an oil bath with stirring for 24 h. After cooling to room temperature, the mixture was diluted with water (30 mL), followed by extraction with CH_2_Cl_2_ (3 × 30 mL). The combined organic extracts were dried over anhydrous Na_2_SO_4_, filtered and concentrated under reduced pressure. The crude residue was purified by silica gel column chromatography (acetone/hexanes, 1:3 v/v) to afford BOPPY **1b** (23 mg, 58%) as a dark solid. Mp 232–235 °C. ^1^H NMR (400 MHz, d_6_-acetone) δ 8.38 (s, 1H), 8.34 – 8.24 (m, 2H), 7.57 (d, *J* = 8.9 Hz, 1H), 7.32 (t, *J* = 6.6 Hz, 1H), 7.27 (d, *J* = 4.0 Hz, 1H), 6.66 (d, *J* = 4.0 Hz, 1H). ^13^C­{1H} NMR (126 MHz, d_6_-acetone) δ 153.0, 146.3, 138.1, 133.2, 126. 7, 125.0, 119.8, 118.0, 117.5, 111.8. ^11^B NMR (128 MHz, CDCl_3_) δ 2.98 (t, *J* = 24.7 Hz), 0.89 (t, *J* = 28.6 Hz). HRMS (ESI-TOF) *m*/*z* [(M-F]^+^ calcd. for C_10_H_7_B_2_BrF_3_N_4_, 340.9992, found 341.0001.

#### 4.2.3 BOPPY **1c**

To a stirring solution of BOPPY **1** (41 mg, 0.145 mmol, 1.00 equiv) in CHCl_3_ (20 mL) at 45 °C, bromine liquid (0.745 mL, 14.6 mmol, 100 equiv) was added dropwise, providing a reaction concentration of 0.0073 M with respect to BOPPY **1**. The reaction mixture was maintained at 45 °C in an oil bath for 3 h, cooled to room temperature and washed with saturated Na_2_S_2_O_3_ solution (3 × 20 mL) to quench excess bromine. The aqueous layers were extracted with CH_2_Cl_2_ (3 × 30 mL) and the combined organic extracts dried over anhydrous Na_2_SO_4_. Removal of the solvent under reduced pressure afforded a crude product, which was purified by silica gel column chromatography (acetone/hexanes, 1:3 v/v) to yield BOPPY **1c** (59 mg, 78%) as a dark solid. Mp 213–215 °C. ^1^H NMR (400 MHz, CDCl_3_) δ 8.04 – 7.97 (m, 2H), 7.78 (s, 1H), 7.61 (d, *J* = 8.9 Hz, 1H), 7.10 (d, *J* = 6.5 Hz, 1H). ^11^B NMR (128 MHz, CDCl_3_) δ 3.05 (t, *J* = 24.8 Hz), 0.50 (t, *J* = 28.2 Hz). HRMS (ESI-TOF) *m*/*z* [(M-F]^+^ calcd. for C_10_H_5_B_2_Br_3_F_3_N_4_, 496.8203, found 496.8210.

#### 4.2.4 General Procedure for Suzuki Cross-Coupling Reactions

To a dry 25 mL round-bottomed flask were added BOPPY (1.00 equiv), tetrabutylammonium bromide (1.00 equiv), the appropriate arylboronic acid (10.0 equiv) and Pd­(PPh_3_)_4_ (10 mol %). The flask was immediately evacuated and refilled with N_2_ three times. Anhydrous toluene (10 mL) and aqueous Na_2_CO_3_ (1.0 M, 10.0 equiv) were then added providing a reaction mixture with the concentration ∼0.005–0.006 M. The resulting mixture was heated at reflux in an oil bath and stirred under N_2_ for 5 h with reaction progress monitored by TLC until complete consumption of the starting material was observed. Upon completion, the mixture was cooled to room temperature, poured into water (30 mL) and extracted with CH_2_Cl_2_ (3 × 30 mL). The combined organic extracts were washed sequentially with brine and water, then dried over anhydrous Na_2_SO_4_. The solvents were removed under reduced pressure and the crude residue was purified by silica gel column chromatography using CH_2_Cl_2_/hexanes (1:2) or EtOAc/hexanes (1:4) as eluents to afford the reported product.

#### 4.2.5 BOPPY **1ba**

This compound was prepared from BOPPY **1b** (20.0 mg, 0.055 mmol) and 4-nitrophenylboronic acid (92.6 mg, 0.554 mmol), yielding the product **1ba** (15.1 mg, 68%) as a yellow solid. Mp 262–264 °C. ^1^H NMR (400 MHz, CDCl_3_) δ 8.31 (d, *J* = 8.9 Hz, 2H), 8.04 (d, *J* = 8.9 Hz, 2H), 7.98 (d, *J* = 6.2 Hz, 1H), 7.93 (ddd, *J* = 8.8, 7.1, 1.6 Hz, 1H), 7.87 (s, 1H), 7.56 (d, *J* = 9.0 Hz, 1H), 7.18 (d, *J* = 4.1 Hz, 1H), 7.03 (ddd, *J* = 7.1, 6.0, 1.0 Hz, 1H), 6.80 (d, *J* = 4.1 Hz, 1H). ^13^C {1H} NMR (126 MHz, CDCl_3_) δ 151.8, 148.2, 145.5, 144.2, 139.1, 136.7, 132.5, 129.7, 126.5, 124.0, 123.7, 118.1, 115.8, 111.8. ^11^B NMR (128 MHz, CDCl_3_) δ 3.02 (t, *J* = 24.8 Hz), 1.41 (t, *J* = 30.9 Hz). HRMS (ESI-TOF) *m*/*z* [(M-F]^+^ calcd. for C_16_H_11_B_2_F_3_N_5_O_2_, 384.1051, found 384.1064.

#### 4.2.6 BOPPY **1bb**

This compound was prepared from BOPPY **1b** (18.2 mg, 0.050 mmol) and 3,5-dimethoxyphenylboronic acid (91.8 mg, 0.504 mmol), yielding the product **1bb** (15.6 mg, 74%) as a yellow solid. Mp 231–233 °C. ^1^H NMR (400 MHz, CDCl_3_) δ 7.93 (d, *J* = 6.3 Hz, 1H), 7.86 (ddd, *J* = 8.8, 7.0, 1.5 Hz, 1H), 7.79 (s, 1H), 7.56 (d, *J* = 9.1 Hz, 1H), 7.14 (d, *J* = 4.1 Hz, 1H), 7.04 (d, *J* = 2.3 Hz, 2H), 6.95 (ddd, *J* = 7.1, 6.2, 0.9 Hz, 1H), 6.71 (d, *J* = 4.1 Hz, 1H), 6.54 (t, *J* = 2.3 Hz, 1H), 3.86 (s, 6H). ^13^C­{1H} NMR (126 MHz, CDCl_3_) δ 160.6, 152.4, 149.5, 143.6, 136.4, 134.5, 132.3, 125.9, 124.4, 117.6, 115.1, 111.8, 107.1, 101.6, 55.6. ^11^B NMR (128 MHz, CDCl_3_) δ 2.99 (t, *J* = 25.1 Hz), 1.42 (t, *J* = 30.6 Hz). HRMS (ESI-TOF) *m*/*z* [(M-F]^+^ calcd. for C_18_H_16_B_2_F_3_N_4_O_2_, 399.1411, found 399.1420.

#### 4.2.7 BOPPY **1bc**

This compound was prepared from BOPPY **1b** (20.6 mg, 0.057 mmol) and dibenzo­[*b,d*]­furan-4-ylboronic acid (121.1 mg, 0.571 mmol), yielding the product **1bc** (20.7 mg, 81%) as a yellow solid. Mp 196–198 °C. ^1^H NMR (400 MHz, CDCl_3_) δ 8.19 (d, *J* = 7.7 Hz, 1H), 8.00 (ddd, *J* = 9.4, 7.7, 1.3 Hz, 2H), 7.93 (d, *J* = 6.1 Hz, 1H), 7.86 (s, 1H), 7.82 (ddd, *J* = 8.8, 7.1, 1.6 Hz, 1H), 7.60 – 7.44 (m, 4H), 7.36 (td, *J* = 7.5, 1.0 Hz, 1H), 7.28 (d, *J* = 4.1 Hz, 1H), 7.21 (d, *J* = 4.1 Hz, 1H), 6.93 (t, *J* = 6.6 Hz, 1H). ^13^C­{1H} NMR (126 MHz, CDCl_3_) δ 156.3, 154.0, 152.4, 143.6, 143.2, 136.4, 132.3, 128.1, 127.4, 125.8, 124.7, 124.3, 124.1, 123.1, 122.8, 121.4, 120.8, 119.7, 117.6, 115.2, 112.0, 111.8. ^11^B NMR (128 MHz, CDCl_3_) δ 3.02 (t, *J* = 25.3 Hz), 1.50 (t, *J* = 30.6 Hz). HRMS (ESI-TOF) *m*/*z* [(M-F]^+^ calcd. for C_22_H_14_B_2_F_3_N_4_O, 429.1306, found 429.1320.

#### 4.2.8 BOPPY **1bd**

This compound was prepared from BOPPY **1b** (20.4 mg, 0.057 mmol) and [1,1′-biphenyl]-2-ylboronic acid (112 mg, 0.566 mmol), yielding the product **1bd** (16.0 mg, 65%) as a yellow solid. Mp 199–201 °C. ^1^H NMR (400 MHz, d_6_-acetone) δ 8.33 (s, 1H), 8.28 (d, *J* = 6.2 Hz, 1H), 8.18 (ddd, *J* = 8.8, 7.1, 1.5 Hz, 1H), 7.78 (d, *J* = 7.7 Hz, 1H), 7.56 – 7.44 (m, 4H), 7.29 – 7.20 (m, 6H), 7.05 (d, *J* = 4.0 Hz, 1H), 5.90 (d, *J* = 4.0 Hz, 1H). ^13^C­{1H} NMR (126 MHz, CDCl_3_) δ 152.3, 149.5, 143.5, 142.2, 141.5, 136.4, 132.0, 131.6, 130.9, 130.0, 129.3, 128.1, 127.9, 126.9, 124.5, 123.6, 119.7, 115.0, 111.6. ^11^B NMR (128 MHz, CDCl_3_) δ 3.01 (t, *J* = 24.8 Hz), 1.42 (t, *J* = 30.5 Hz). HRMS (ESI-TOF) *m*/*z* [(M-F]^+^ calcd. for C_22_H_16_B_2_F_3_N_4_, 415.1513, found 415.1519.

#### 4.2.9 BOPPY **1be**

This compound was prepared from BOPPY **1b** (16.8 mg, 0.047 mmol) and 1-(*tert*-butoxycarbonyl)-1*H*-pyrrol-2-yl)­boronic acid (98.3 mg, 0.466 mmol), yielding the product **1be** (6.0 mg, 29%) as a yellow solid. Mp 187–189 °C ^1^H NMR (400 MHz, CDCl_3_) δ 7.92 (d, *J* = 6.2 Hz, 1H), 7.82 (ddd, *J* = 8.8, 7.1, 1.5 Hz, 1H), 7.78 (s, 1H), 7.49 (d, *J* = 8.7 Hz, 1H), 7.46 (dd, *J* = 3.3, 1.8 Hz, 1H), 7.10 (d, *J* = 4.1 Hz, 1H), 6.94 (ddd, *J* = 7.2, 6.1, 1.0 Hz, 1H), 6.56 (d, *J* = 4.0 Hz, 1H), 6.52 (dd, *J* = 3.4, 1.8 Hz, 1H), 6.30 (t, *J* = 3.3 Hz, 1H), 1.33 (s, 9H). ^13^C­{1H} NMR (126 MHz, CDCl_3_) δ 152.4, 149.0, 143.6, 141.1, 136.3, 132.2, 124.7, 124.2, 123.4, 123.1, 119.1, 117.2, 115.1, 111.8, 110.8, 83.6, 27.7. ^11^B NMR (128 MHz, CDCl_3_) δ 2.98 (t, *J* = 25.0 Hz), 1.11 (t, *J* = 29.7 Hz). HRMS (ESI-TOF) *m*/*z* [(M+Na]^+^ calcd. for C_19_H_19_B_2_F_4_N_5_NaO_2_, 470.1559, found 470.1570.

#### 4.2.10 BOPPY **1bf**

This compound was prepared from BOPPY 1b (20.8 mg, 0.057 mmol) and benzo­[b]­thiophen-2-ylboronic acid (103 mg, 0.577 mmol), yielding the product **1bf** (17.3 mg, 72%) as a yellow solid. Mp 201–203 °C. ^1^H NMR (400 MHz, CDCl_3_) δ 8.15 (s, 1H), 7.97 (d, *J* = 6.2 Hz, 1H), 7.93 (ddd, *J* = 8.8, 7.1, 1.5 Hz, 1H), 7.90 – 7.86 (m, 1H), 7.84 – 7.81 (m, 1H), 7.80 (s, 1H), 7.67 (d, *J* = 8.9 Hz, 1H), 7.38 – 7.34 (m, 2H), 7.14 (d, *J* = 4.2 Hz, 1H), 7.01 (ddd, *J* = 7.1, 6.2, 1.0 Hz, 1H), 6.93 (d, *J* = 4.2 Hz, 1H). ^13^C­{1H} NMR (126 MHz, CDCl_3_) δ 143.8, 141.9, 140.9, 140.1, 136.5, 133.7, 131.5, 126.4, 125.3, 125.0, 124.8, 124.8, 124.3, 122.0, 118.5, 115.4, 112.0. ^11^B NMR (128 MHz, CDCl_3_) 3.04 (t, *J* = 24.7 Hz), 1.43 (t, *J* = 30.8 Hz). HRMS (ESI-TOF) *m*/*z* [(M+H]^+^ calcd. for C_18_H_13_B_2_F_4_N_4_S, 415.0983, found 415.0991.

#### 4.2.11 BOPPY **1bg**

This compound was prepared from BOPPY **1b** (16.7 mg, 0.046 mmol) and 2-formylphenylboronic acid (69.0 mg, 0.463 mmol), yielding the product **1bg** (7 mg, 39%) as a yellow solid. Mp 195–197 °C. ^1^H NMR (400 MHz, CDCl_3_) δ 9.85 (s, 1H), 8.07 (dt, *J* = 7.7, 1.1 Hz, 1H), 7.95 (d, *J* = 6.3 Hz, 1H), 7.88 (s, 1H), 7.83 (ddd, *J* = 8.9, 7.1, 1.5 Hz, 1H), 7.71 – 7.67 (m, 2H), 7.63 – 7.58 (m, 1H), 7.42 (d, *J* = 9.1 Hz, 1H), 7.19 (d, *J* = 4.0 Hz, 1H), 6.98 (td, *J* = 6.7, 6.1, 1.0 Hz, 1H), 6.59 (d, *J* = 3.9 Hz, 1H). ^13^C­{1H} NMR (126 MHz, CDCl_3_) δ 191.4, 152.1, 143.9, 136.5, 136.3, 135.3, 133.3, 132.7, 131.3, 129.6, 127.4, 125.5, 123.1, 119.7, 115.5, 111.8. ^11^B NMR (128 MHz, CDCl_3_) δ 2.99 (t, *J* = 25.0 Hz), 1.21 (t, *J* = 30.3 Hz). HRMS (ESI-TOF) *m*/*z* [(M-F]^+^ calcd. for C_17_H_12_B_2_F_3_N_4_O, 367.1149, found 367.1152.

#### 4.2.12 BOPPY **1bh**

This compound was prepared from BOPPY **1b** (21.2 mg, 0.059 mmol) and 4-trifluoromethylphenylboronic acid (112 mg, 0.588 mmol), yielding the product **1bh** (18.4 mg, 73%) as a yellow solid. Mp 293–295 °C. ^1^H NMR (400 MHz, CDCl_3_) δ 8.01 – 7.94 (m, 3H), 7.89 (ddd, *J* = 8.9, 7.1, 1.6 Hz, 1H), 7.84 (s, 1H), 7.72 (d, *J* = 8.2 Hz, 2H), 7.56 (d, *J* = 9.0 Hz, 1H), 7.17 (d, *J* = 4.0 Hz, 1H), 7.03 – 6.96 (m, 1H), 6.74 (d, *J* = 4.1 Hz, 1H). ^13^C­{1H} NMR (126 MHz, CDCl_3_) δ 152.3, 147.3, 144.0, 136.6, 136.3, 132.5, 129.2, 128.8, 126.3, 125.4, 124.2, 123.2, 117.8, 115.5, 111.8. ^11^B NMR (128 MHz, CDCl_3_) δ 3.01 (t, *J* = 24.7 Hz), 1.42 (t, *J* = 30.3 Hz). HRMS (ESI-TOF) *m*/*z* [(M-F]^+^ calcd. for C_17_H_11_B_2_F_6_N_4_, 407.1074, found 407.1084.

#### 4.2.13 BOPPY **1be’**

To a stirred solution of BOPPY **1be** (11.1 mg, 0.024 mmol) in CH_2_Cl_2_ (5 mL) was added trifluoroacetic acid (0.3 mL) dropwise. The reaction mixture was stirred at room temperature for 3 h, during which complete consumption of the starting material was confirmed by TLC. The reaction mixture was quenched with saturated NaHCO_3_ solution (10 mL) and extracted with CH_2_Cl_2_ (3 × 20 mL). The combined organic extracts were washed sequentially with brine (20 mL) and water (20 mL) dried over anhydrous Na_2_SO_4_ and concentrated under reduced pressure. Purification by silica gel column chromatography (CH_2_Cl_2_/hexane, 3:1 v/v) afforded BOPPY **1be’** (9 mg, 98%) as a yellow solid. Mp 216–218 °C. ^1^H NMR (400 MHz, CDCl_3_) δ 8.05 – 7.98 (m, 2H), 7.83 – 7.76 (m, 3H), 7.14 (d, *J* = 4.2 Hz, 1H), 7.08 (t, *J* = 6.7 Hz, 1H), 6.87 – 6.81 (m, 2H), 6.38 (t, *J* = 3.4 Hz, 1H). ^13^C­{1H} NMR (126 MHz, CDCl_3_) δ 207.1, 151.7, 148.6, 144.3, 138.0, 136.6, 131.2, 127.7, 124.7, 124.0, 122.9, 116.9, 116.1, 115.1, 112.1. ^11^B NMR (128 MHz, CDCl_3_) δ 2.96 (t, *J* = 24.7 Hz), 0.63 (d, *J* = 44.7 Hz). HRMS (ESI-TOF) *m*/*z* [(M-F]^+^ calcd. for C_15_H_11_B_2_F_3_N_5_O_2_, 372.1051, found 372.1054.

#### 4.2.14 General Procedure for Stille Cross-Coupling Reactions

To a dry 25 mL round-bottomed flask were added BOPPY (1.00 equiv), organotin reagent (5–10 equiv) and Pd­(PPh_3_)_4_ (10 mol %). The flask was evacuated and refilled with N_2_ three times. Anhydrous toluene (10 mL) was added providing a reaction concentration of **∼**0.003–0.004 M. The reaction mixture was heated at reflux in an oil bath and stirred under N_2_ for 4–6 h with reaction progress monitored by TLC until complete consumption of the starting material. The reaction mixture was cooled to room temperature, poured into water (30 mL) and extracted with CH_2_Cl_2_ (3 × 30 mL). The combined organic extracts were washed sequentially with brine and water, dried over anhydrous Na_2_SO_4_ and concentrated under reduced pressure. The crude residue was purified by silica gel column chromatography (CH_2_Cl_2_/hexanes, 1:2 v/v) to afford the desired coupled product.

#### 4.2.15 BOPPY **1bi**

This compound was prepared from BOPPY **1b** (15.4 mg, 0.043 mmol) and 2-(tributylstannyl)­thiophene (65 μL, 0.208 mmol), yielding the product **1bi** (14.2 mg, 91%) as a yellow solid. Mp 214–216 °C. ^1^H NMR (400 MHz, CDCl_3_) δ 7.98 – 7.84 (m, 3H), 7.75 (s, 1H), 7.61 (d, *J* = 8.9 Hz, 1H), 7.40 (d, *J* = 5.0 Hz, 1H), 7.16 (dd, *J* = 5.1, 3.7 Hz, 1H), 7.10 (d, *J* = 4.3 Hz, 1H), 6.96 (t, *J* = 6.5 Hz, 1H), 6.83 (d, *J* = 4.2 Hz, 1H). ^13^C­{1H} NMR (126 MHz, CDCl_3_) δ 152.2, 143.6, 142.3, 136.4, 134.2, 131.4, 128.5, 127.1, 125.8, 124.5, 121.4, 117.6, 115.2, 111.8. ^11^B NMR (128 MHz, CDCl_3_) δ 2.98 (t, *J* = 24.8 Hz), 1.34 (t, *J* = 30.9 Hz). HRMS (ESI-TOF) *m*/*z* [(M-F]^+^ calcd. for C_14_H_10_B_2_F_3_N_4_S, 345.0764, found 345.0769.

#### 4.2.16 BOPPY **1ca**

This compound was prepared from BOPPY **1c** (15.1 mg, 0.028 mmol) and 2-(tributylstannyl)­thiophene (92 μL, 0.291 mmol), yielding the product **1ca** (14.6 mg, 96%) as a yellow solid. Mp 238–240 °C. ^1^H NMR (400 MHz, CDCl_3_) δ 8.02 – 7.96 (m, 2H), 7.87 (s, 1H), 7.61 (d, *J* = 9.1 Hz, 1H), 7.54 (dd, *J* = 5.1, 1.2 Hz, 1H), 7.31 (dd, *J* = 3.6, 1.2 Hz, 1H), 7.22 (dd, *J* = 5.1, 3.6 Hz, 1H), 7.07 (t, *J* = 6.2 Hz, 1H). ^13^C­{1H} NMR (126 MHz, CDCl_3_) δ 152.1, 144.5, 136.7, 131.0, 130.4, 130.2, 129.2, 128.4, 128.2, 123.2, 121.0, 116.2, 111.8, 107.8. ^11^B NMR (128 MHz, CDCl_3_) δ 3.03 (t, *J* = 25.0 Hz), 0.65 (t, *J* = 28.5 Hz). HRMS (ESI-TOF) *m*/*z* [(M-F]^+^ calcd. for C_14_H_8_B_2_Br_2_F_3_N_4_S, 500.8975, found 500.8992.

#### 4.2.17 General Procedure for Nucleophilic Substitution Reactions

To a dry 25 mL round-bottomed flask charged with the corresponding BOPPY precursor (1.00 equiv) were added 4-methoxythiophenol (∼85–100 equiv) and trimethylamine (1.2 equiv) in 10 mL of an appropriate solvent (o-xylene or CHCl_3_) providing a reaction concentration of ∼0.004–0.005 M. The reaction mixture was heated in an oil bath at elevated temperature (60 °C to reflux) and stirred for 6–24 h, with the reaction progress monitored by TLC. After completion, the reaction mixture was cooled to room temperature, diluted with water (10 mL) and extracted with CH_2_Cl_2_ (3 × 30 mL). The combined organic layers were washed sequentially with brine and water, dried over anhydrous Na_2_SO_4_, and concentrated under reduced pressure. The crude product was purified by silica gel column chromatography using acetone/hexanes or CH_2_Cl_2_/hexanes mixtures as the eluent to afford the corresponding thiophenol-substituted BOPPY derivative.

#### 4.2.18 BOPPY **1bj**

This compound was prepared from BOPPY **1b** (14.6 mg, 0.040 mmol) and 4-methoxythiophenol (0.50 mL, 4.00 mmol) under reflux in o-xylene for 24 h to afford BOPPY **1bj** (6.5 mg, 38%) as a yellow solid. Mp 217–219 °C. ^1^H NMR (400 MHz, CDCl_3_) δ 7.95 – 7.85 (m, 2H), 7.64 (s, 1H), 7.61 – 7.54 (m, 3H), 6.98 – 6.90 (m, 4H), 5.99 (dd, *J* = 4.1, 1.5 Hz, 1H), 3.84 (d, *J* = 1.5 Hz, 3H). ^13^C­{1H} NMR (126 MHz, CDCl_3_) δ 160.83, 147.4, 143.5, 136.5, 133.2, 130.5, 125.6, 124.1, 121.5, 117.0, 115.3, 115.0, 114.7, 111.7, 55.6. ^11^B NMR (128 MHz, CDCl_3_) δ 2.97 (t, *J* = 25.1 Hz), 1.04 (t, *J* = 30.6 Hz). HRMS (ESI-TOF) *m*/*z* [(M+H]^+^ calcd. for C_17_H_15_B_2_F_4_N_4_OS, 421.1089, found 421.1098.

#### 4.2.19 BOPPY **1cb**

This compound was prepared from BOPPY **1c** (24 mg, 0.046 mmol) and 4-methoxythiophenol (0.48 mL, 3.93 mmol) heated to 60 °C in CHCl_3_ for 6 h to afford BOPPY **1cb** (11 mg, 41%) as a yellow solid. Mp 219–221 °C. ^1^H NMR (400 MHz, CDCl_3_) δ 8.04 – 7.96 (m, 2H), 7.82 (s, 1H), 7.65 (d, *J* = 9.0 Hz, 1H), 7.48 (d, *J* = 8.9 Hz, 2H), 7.09 (td, *J* = 6.5, 6.1, 1.0 Hz, 1H), 6.82 (d, *J* = 8.8 Hz, 2H), 3.77 (s, 3H). ^13^C­{1H} NMR (126 MHz, CDCl_3_) δ 159.8, 152.3, 144.7, 137.6, 136.8, 133.8, 131.7, 129.9, 124.3, 123.7, 116.3, 114.8, 113.2, 112.0, 55.5. ^11^B NMR (128 MHz, CDCl_3_) δ 3.05 (t, *J* = 24.8 Hz), 0.86 (t, *J* = 28.7 Hz). HRMS (ESI-TOF) *m*/*z* [(M-F]^+^ calcd. for C_17_H_12_B_2_Br_2_F_3_N_4_OS, 556.9237, found 556.9248.

#### 4.2.20 BOPPY **1cc**

This compound was prepared from BOPPY **1c** (24 mg, 0.046 mmol) and 4-methoxythiophenol (0.48 mL, 3.93 mmol) heated to 60 °C in CHCl_3_ for 6 h to afford BOPPY **1cc** (20 mg, 68%) as a yellow solid. Mp 216–218 °C. ^1^H NMR (400 MHz, CDCl_3_) δ 8.03 – 7.94 (m, 3H), 7.66 (d, *J* = 9.0 Hz, 1H), 7.46 (d, *J* = 8.9 Hz, 2H), 7.29 – 7.21 (m, 2H), 7.07 (t, 1H), 6.86 – 6.78 (m, 4H), 3.81 – 3.75 (m, 6H). ^13^C­{1H} NMR (126 MHz, CDCl_3_) δ 159.6, 159.4, 152.3, 144.6, 137.4, 136.8, 133.3, 131.7, 130.0, 127.7, 127.6, 125.1, 124.2, 119.5, 116.3, 115.2, 114.8, 112.0, 55.5, 55.4. ^11^B NMR (128 MHz, CDCl_3_) δ 2.92 (d, *J* = 25.5 Hz), 0.93 (t, *J* = 28.7 Hz). HRMS (ESI-TOF) *m*/*z* [(M-F]^+^ calcd. for C_24_H_19_B_2_BrF_3_N_4_O_2_S_2_, 617.0271, found 617.0293.

### 4.3 X-ray Crystallographic Analyses

Single crystals suitable for X-ray diffraction were obtained by slow diffusion of solutions of the corresponding compounds in CH_2_Cl_2_/hexanes (1:1) at room temperature over several days. Crystal structures of the 15 compounds were determined at low temperature using data collected on a Bruker D8 Venture DUO diffractometer with a Photon III detector and CuKα radiation (AgKα for **1ca**). There were two independent molecules for **1bd**, **1be’**, **1bf**, and **1ca**, three for **1bh** and four for **1bc**. Crystals of **1ba**, **1bc**, **1bd**, and **1be’** were twins. Disordered solvent contribution was removed for **1bc** and **1ca** using the SQUEEZE procedure. **1bh** had a disordered CF_3_ group and **1ca** had a disordered DCM solvent molecule. Small amounts of a cocrystallized impurity were present, typically at the 2-position for **1b**, **1bd**, **1be’**, **1bh**, and **1bj** and at the 3-position for **1bi**. Most H atoms were visible in difference maps but were placed in idealized positions for refinement using SHELXL.

### 4.4 Spectroscopic Analysis

UV–vis absorption and emission spectra were collected at room temperature, on a Perkin-Elmer spectrophotometer and a Perkin-Elmer LS55 spectrophotometer, respectively. Dilute solutions (ca. 10^–6^ M) from spectrophotometric grade solvents in quartz cuvettes (1 cm path length) were used to minimize reabsorption effects. The relative fluorescence quantum yields (Φ_F_) were calculated using BOPPY **1** (Φ_F_ = 0.79 in DCM and 0.87 in toluene) as reference using the following equation:^30^ Φ_X_ = Φ_ST_ × Grad_X_/Grad_ST_ × (η_X_/η_ST_),^2^ where the Φ_X_ and Φ_ST_ are the quantum yields of the sample and standard, Grad_X_ and Grad_ST_ are the gradients from the plot of integrated fluorescence intensity vs absorbance, and η represents the refractive index of the solvent.

### 4.5 Theoretical Calculations

All ground and excited states were studied at the MN15/6-311++G­(d,p) levels of theory,^31^ taking into account the solvent effects using the Polarized Continuum Model (PCM). The MN15 functional was recommended in a recent benchmark study of the photophysical properties of difluoroborane and hydroxyphenyllimidazol dyes^32^ and a recent study from our group^33^ that demonstrated that MN15 predicts the tendencies of the bathochromic and hypsochromic shifts for a series of BODIPYs better than 3 other functionals. The absorption and emission data were calculated using TD-DFT. The first ten singlet excitations were considered, and the lowest-energy excited singlet state was optimized to calculate the properties reported in this study. The SI contains a list with the Z-matrices, the number of imaginary frequencies, and computed total energies of optimized structures, along with any absolute energy values used to calculate results discussed herein. All calculations were performed using the Gaussian 16 program package.^34^

## Supplementary Material



## Data Availability

The data underlying this study are available in the published article and its Supporting Information.

## Notes

NMR and HRMS data for all BOPPYs, absorption and emission spectra, X-ray data (CIF and summary tables of crystal parameters), and theoretical calculations data (PDF)

## Abbreviations

PTSA: *p*-toluenesulfonic acid
DBU: 1,8-diazabicyclo­[5.4.0]­undec-7-ene
TD-DFT: time-dependent density-functional theory
NMR: nuclear magnetic resonance
TLC: thin-layer chromatography
HRMS: high-resolution mass spectrometry
MESPs: molecular electrostatic potentials
PCM: polarized continuum model
HOMO: highest occupied molecular orbital
LUMO: lowest unoccupied molecular orbital
