# Supplementary material for: Regioselective Halogenation of BOPPY Fluorophores and Subsequent Diversification via Cross-Coupling and Aromatic Nucleophilic Substitution Strategies
Source: J Org Chem. 2026 Mar 10;91(11):4021–32. doi: 10.1021/acs.joc.5c03121 (PMC13010255; doi:10.1021/acs.joc.5c03121)
Supplement: Supplementary file 1 [file jo5c03121_si_001.pdf]

Supporting information

Regioselective Halogenation of BOPPY Fluorophores and Subsequent Diversification via Cross-Coupling and Aromatic Nucleophilic Substitution Strategies

Sebastian O. Oloo,[1] Petia Bobadova-Parvanova,[2] Alexis A. Lueders[2], Mina Kim[2], Frank R. Fronczek,[1] Kevin M. Smith,[1] and Maria da Graça H. Vicente[1,*]

Table of Contents

1. Tables S3

2. Photophysical data S6

3. 1D NMR SPECTRA S12

4. Computational studies data S47

   Cartesian Coordinates for BOPPY compounds S50

5. Crystallographic Data and Structure Parameters S83

1. Tables

Table S 1. Photophysical properties of BOPPYs in dichloromethane, toluene and acetonitrile at room temperature

	λ_{abs}^{max}/nm (log ε_{max})	λ_{em}/nm	Stokes Shift/cm^{-1}	Φ_F [a]	λ_{abs}^{max}/nm (log ε_{max})	λ_{em}/nm	Stokes Shift/cm^{-1}	Φ_F [c]	λ_{abs}^{max}/nm (log ε_{max})	λ_{em}/nm	Stokes Shift/cm^{-1}	Φ_F [c]
	DICHLOROMETHANE				TOLUENE				ACETONITRILE			
**1**	396(4.52) 416(4.51)	433, 457	2400	0.79[b]	399(4.54) 419(4.54)	436, 459	2100	0.87[d]				
**1a**	400(4.43) 421(4.46)	437, 459	900	0.72	403(4.45) 425(4.49)	438,464	700	0.87	393(4.64) 412(4.64)	435, 456	1300	0.28
**1b**	402(4.36) 422(4.39)	438, 463	900	0.74	404(4.45) 425(4.49)	440,464	700	0.89	394(4.49) 413(4.50)	437, 456	1300	0.32
**1c**	406(4.58) 428(4.66)	442, 469	700	0.57	411(4.46) 434(4.56)	447,474	800	0.77	400(4.59) 419(4.63)	439, 461	1100	0.13
**1ba**	428(4.43) 447(4.36)	480b	2500	0.40	426(4.39) 449s(4.29)	473,505	2300	0.78	421(4.58)	460b	2000	0.01
**1bb**	422(4.55) 439(4.55)	466, 492	1300	0.95	423(4.54) 443(4.55)	467,496	1200	0.90	414(4.67) 427(4.67)	464, 490s	1800	0.66
**1bc**	425(4.57) 441(4.49)	469, 499	2200	0.98	427(4.56) 446(4.52)	472, 497	2200	0.94	413(4.60)	462	2600	0.57
**1bd**	414(4.47) 430(4.49)	466	1800	0.85	418(4.51) 435(4.52)	466,493s	1500	0.80	409(493) 422(4.95)	465	2200	0.53
**1be'**	448(4.40) 467(4.48)	497	1300	0.90	447(4.44) 474(4.54)	496,526s	900	1.00	433(4.48) 454(4.48)	492	1700	0.17
**1bf**	440(4.33) 464(4.35)	488,515s	1100	1.00	443(4.44) 470(4.48)	486,518	700	1.00	431(4.52) 452(4.52)	482, 513s	1400	0.95
**1bg**	405(4.21) 419(4.24)	457	2000	0.76	407(4.45) 427(4.50)	456,484s	1500	0.72	399(4.50) 415(4.52)	452	2000	0.35
**1bh**	414(4.53) 433(4.54)	457,483s	1200	0.89	417(4.56) 437(4.58)	459,481	1100	0.78	412(4.64) 425(4.64)	455,478s	1600	0.48
**1bi**	403(4.19) 423(4.27)	438, 466	700	0.82	405(4.25) 427(4.33)	440, 470	700	0.79	429(4.69) 447(4.67)	480, 515s	2500	0.37

**1bj**	429(4.58)<br>451(4.54)	440, 470	500	0.21	430(4.49)<br>455(4.45)	440, 475	500	0.52	421(4.69)<br>437(4.67)	437, 474	2700	0.01
**1ca**	420(4.41)<br>436(4.48)	477	2000	0.09	424(4.41)<br>443(4.48)	480	1700	0.11	412(4.58)<br>427(4.62)	477b	2500	0.04
**1cb**	414s<br>434(4.45)	-	-	0.00	423s<br>440(4.46)	-	-	0.00	425(4.58)	-	-	0.00
**1cc**	417s,<br>438(4.35)	-	-	0.00	428s,<br>445(4.40)	-	-	0.00	429(4.54)	-	-	0.00

[a]Fluorescence quantum yields (Φ_F) determined using BOPPY 1 in DCM (Φ_F = 0.79) as standard. [b]Reported fluorescence quantum yield in DCM is Φ_F = 0.79.
[c]Fluorescence quantum yields (Φ_F) determined using BOPPY 1 in Toluene (Φ_F = 0.87) as standard. [d]Reported fluorescence quantum yield in toluene is Φ_F = 0.87.

Table S 2. TD-DFT MN15/6-311++G(d,p) calculated spectroscopic, electronic, and structural properties of the ground and excited states of the series of BOPPYs studied. All parameters are calculated in DCM

Compound	λ_{abs} (nm)	Oscillator Strength	λ_{em} (nm)	Stokes shift (nm)	HOMO (eV)	LUMO (eV)	E_g (eV)	$E(S_2)$-$E(S_1)$ (eV)	$R_{NN}(S_1)$-$R_{NN}(S_0)$ (Å)
**1**	361	0.75	424	63	-7.05	-1.98	5.07	0.92	-0.046
**1a**	366	0.78	428	62	-7.04	-2.04	5.00	0.95	-0.043
**1b**	369	0.79	431	62	-7.02	-2.05	4.97	0.96	-0.042
**1c**	393	0.86	442	49	-7.17	-2.29	4.89	0.90	-0.034
**1ba**	403	0.93	417	14	-6.99	-2.49	4.49	0.59	-0.035
**1bb**	386	0.91	401	15	-6.77	-2.01	4.77	0.57	-0.041
**1bc**	393	0.99	407	14	-6.74	-2.05	4.68	0.93	-0.038
**1bd**	381	0.86	393	12	-6.81	-1.97	4.83	0.89	-0.038
**1be′**	386	0.70	398	12	-6.81	-2.05	4.76	0.82	-0.034
**1bf**	406	1.05	421	15	-6.62	-2.11	4.51	0.82	-0.029
**1bg**	370	0.79	382	12	-7.04	-2.05	4.99	0.23	-0.038
**1bh**	386	0.91	400	14	-6.91	-2.12	4.79	0.98	-0.040
**1bi**	408	0.93	486	78	-6.58	-2.07	4.51	0.99	-0.034
**1bj**	391	0.99	458	67	-6.60	-1.92	4.68	0.95	-0.040
**1ca**	391	0.85	491	100	-7.01	-2.26	4.75	0.56	-0.026
**1cb**	384	0.73	–	–	-7.02	-2.31	4.71	0.31	–
**1cc**	400	0.66	–	–	-6.85	-2.29	4.57	0.24	–

* S_1 for **1cb** and **1cc** did not converge

2. Photophysical data

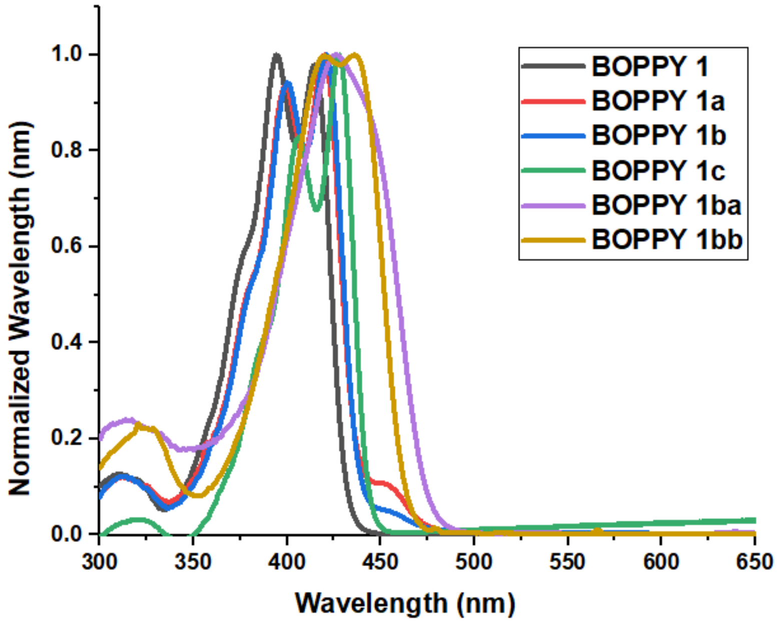

Figure S 1. Absorbance spectra for BOPPYs 1, 1a,1b,1c,1ba and 1bb in dichloromethane

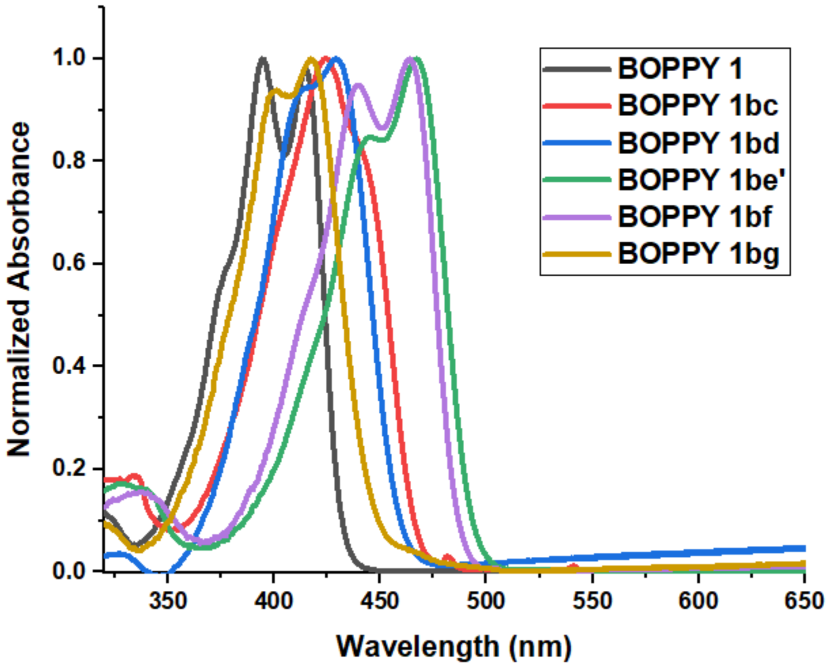

Figure S 2. Absorbance spectra for BOPPYs 1, 1bc,1bd,1be’,1bf and 1bg in dichloromethane

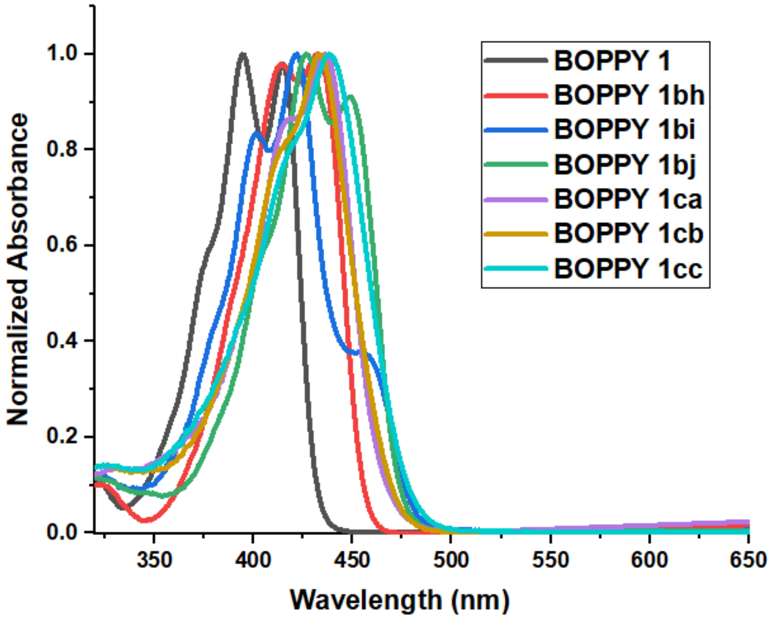

Figure S 3. Absorbance spectra for BOPPYs 1, 1bh, 1bi, 1bj, 1ca, 1cb and 1cc in dichloromethane

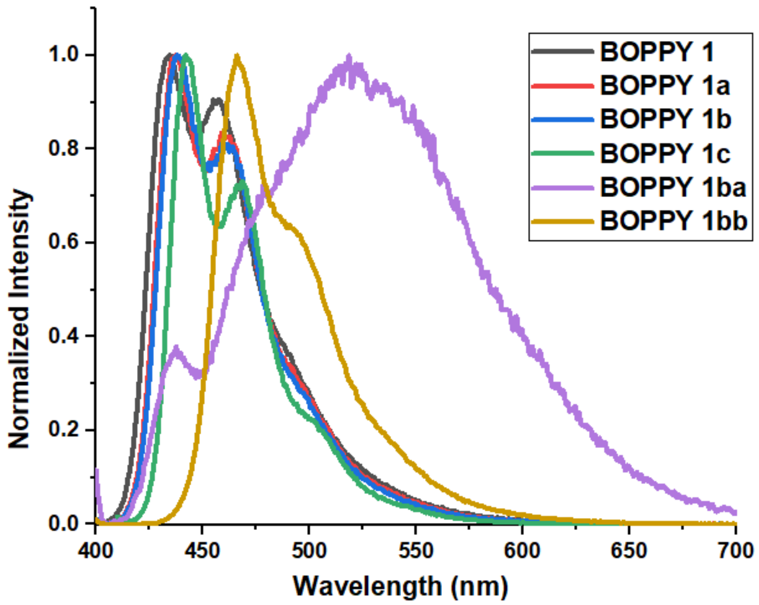

Figure S 4. Emission spectra for BOPPYs 1, 1a,1b,1c,1ba and 1bb in dichloromethane

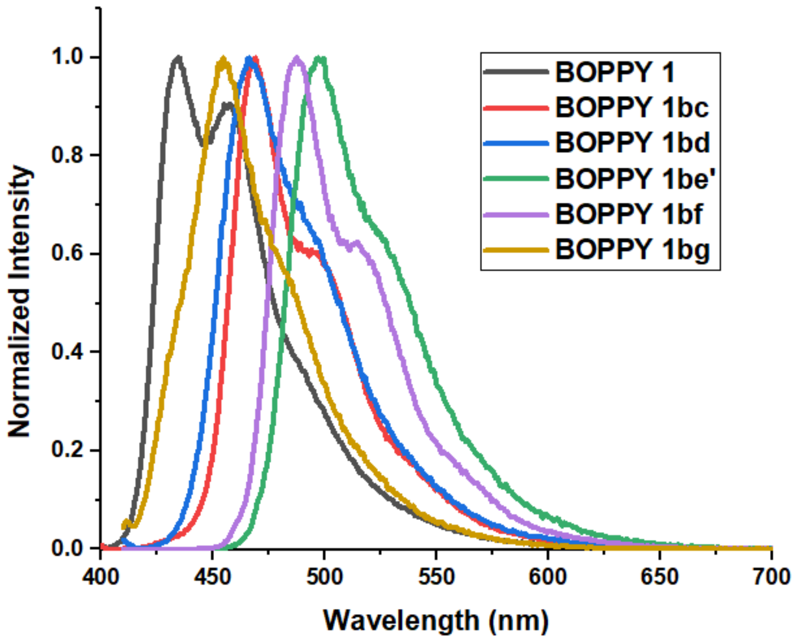

Figure S 5. Emission spectra for BOPPYs 1, 1bc,1bd,1be',1bf and 1bg in dichloromethane

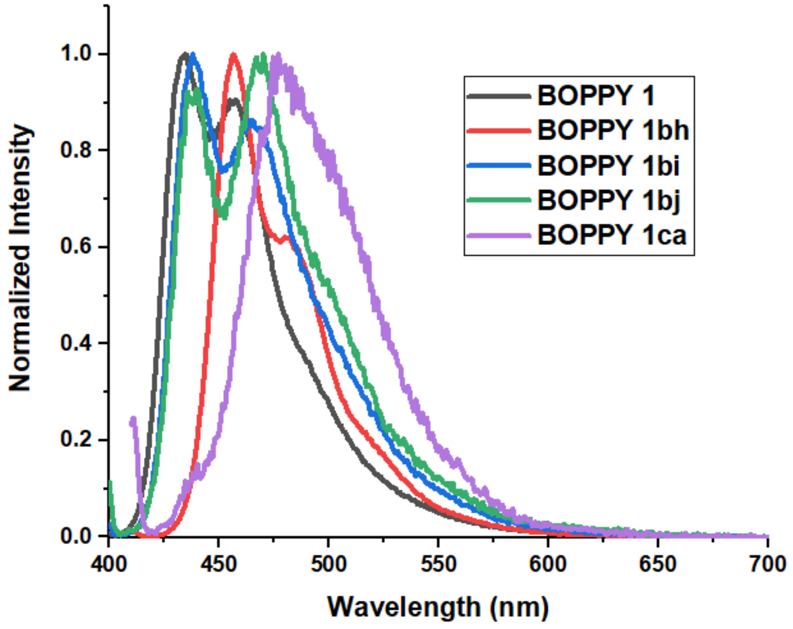

Figure S 6. Emission spectra for BOPPYs 1, 1bh,1bi,1bj and 1ca in dichloromethane

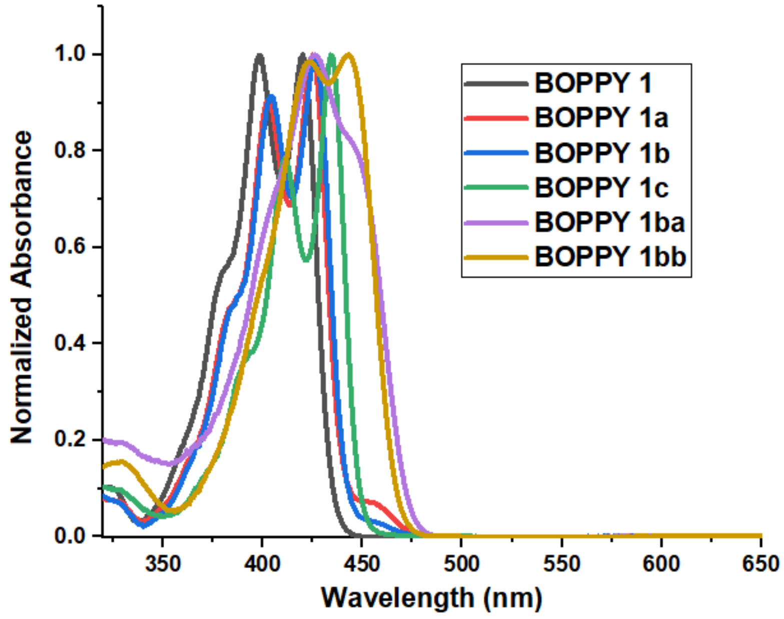

Figure S 7. Absorbance spectra for BOPPYs 1, 1a,1b,1c,1ba and 1bb in Toluene

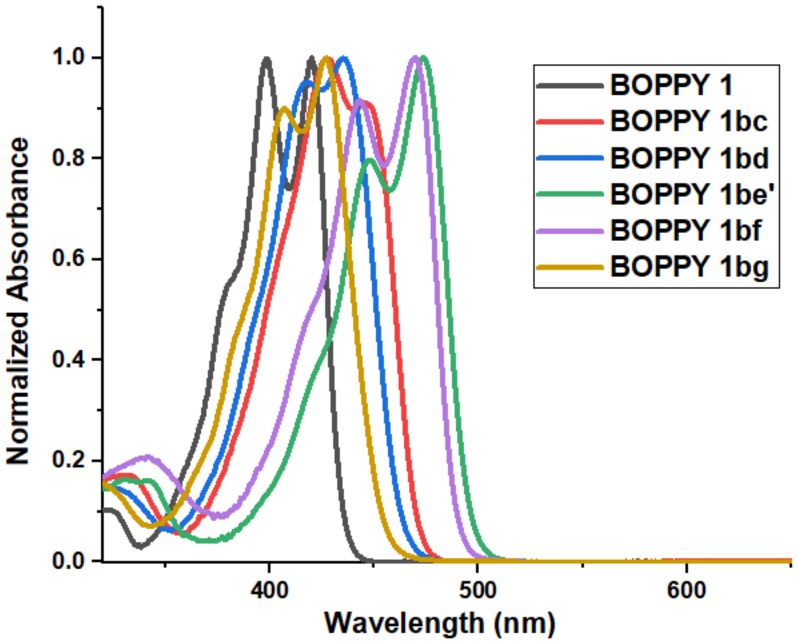

Figure S 8. Absorbance spectra for BOPPYs 1, 1bc,1bd,1be',1bf and 1bg in Toluene

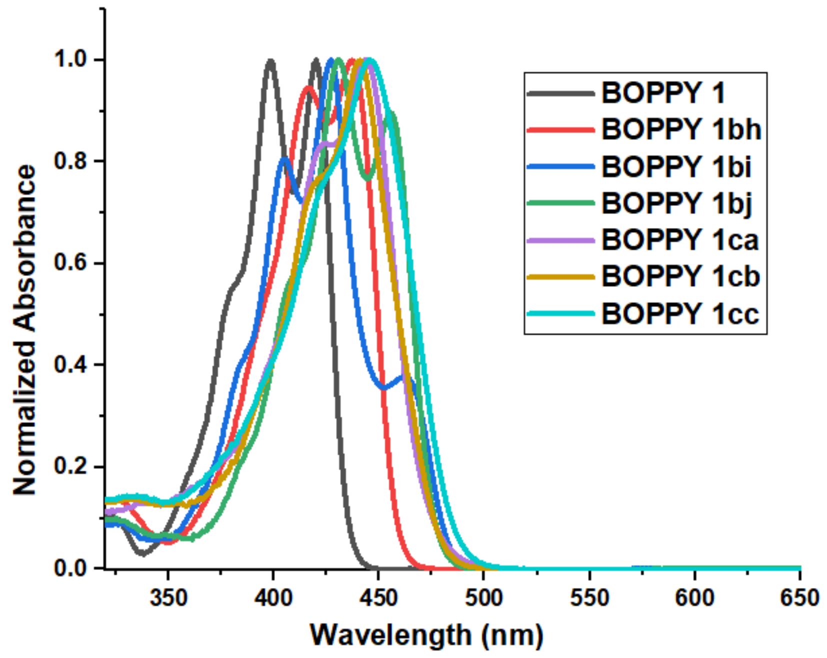

Figure S 9. Absorbance spectra for BOPPYs 1, 1bh,1bi,1bj,1ca,1cb and 1cc in Toluene

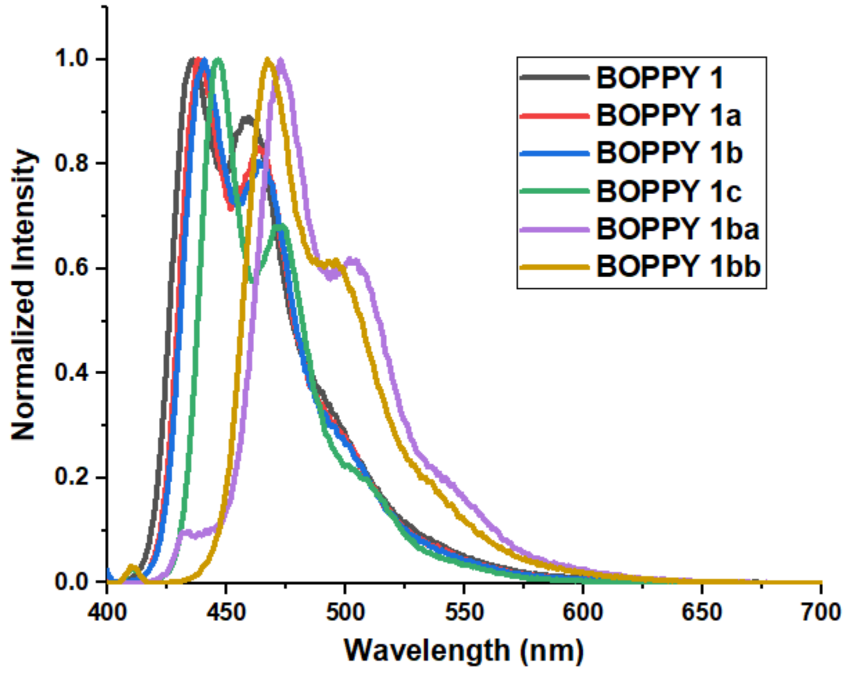

Figure S 10. Emission spectra for BOPPYs 1, 1a,1b,1c,1ba and 1bb in Toluene

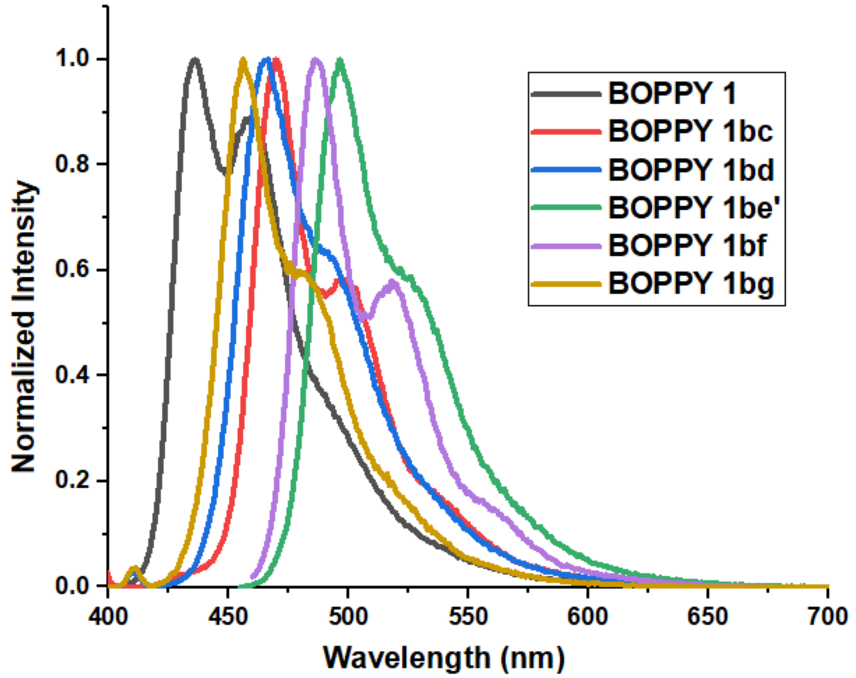

Figure S 11. Emission spectra for BOPPYs 1, 1bc,1bd,1be',1bf and 1bg in Toluene

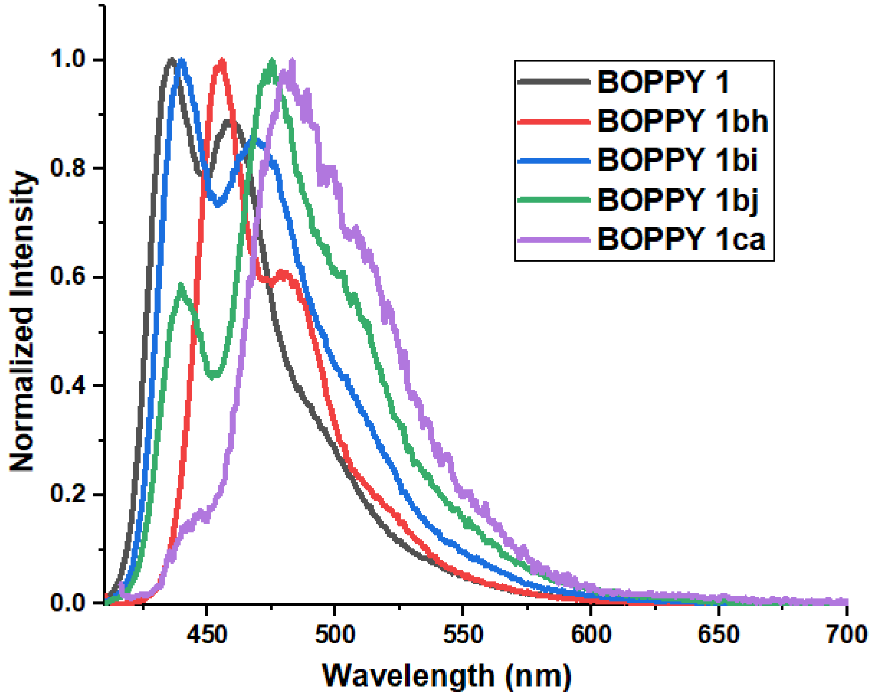

Figure S 12. Emission spectra for BOPPYs 1, 1bh,1bi,1bj and 1ca in Toluene

3. 1D NMR SPECTRA

BOPPY 1

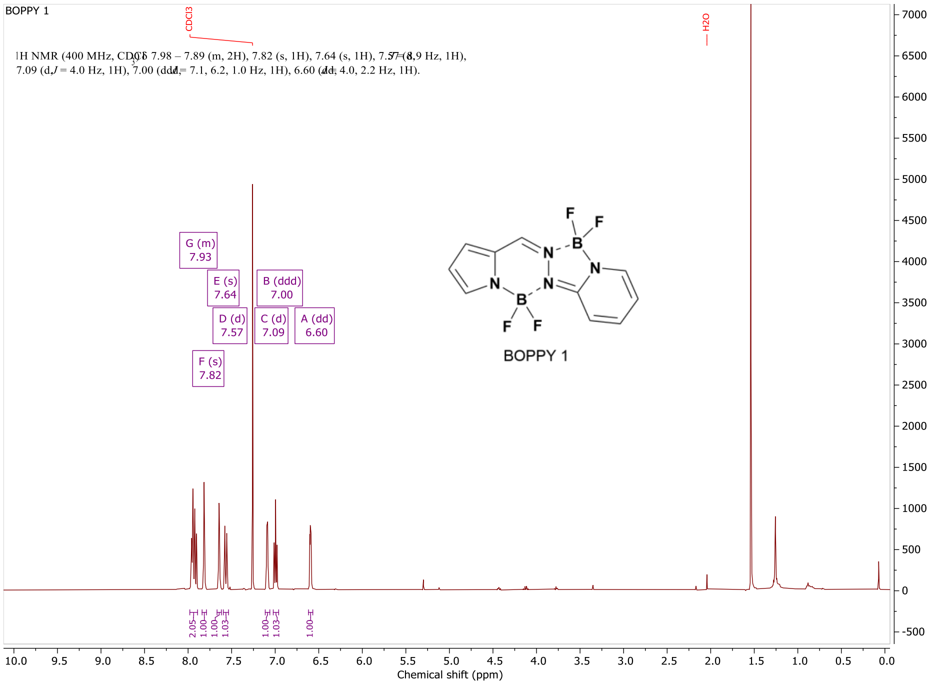

Figure S 13. ^{1}H NMR (400 MHz, CDCl$_3$) Spectrum

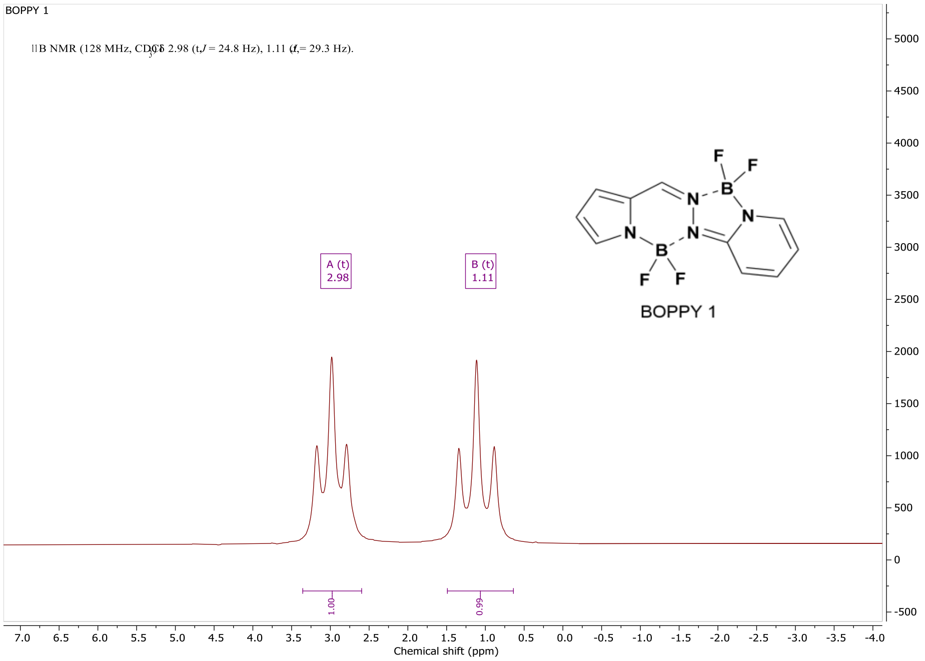

Figure S 14. ^{11}B NMR (128 MHz, CDCl$_3$) Spectrum

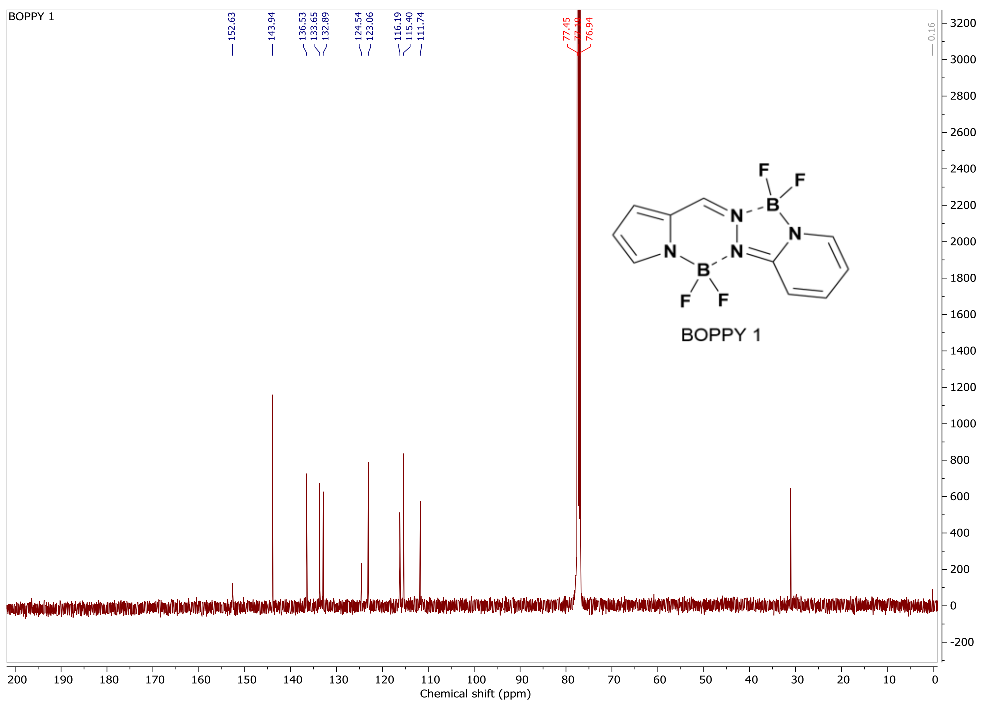

Figure S 15. $^{13}C\{1H\}$ NMR (126 MHz, $CDCl_3$) Spectrum

BOPPY 1a

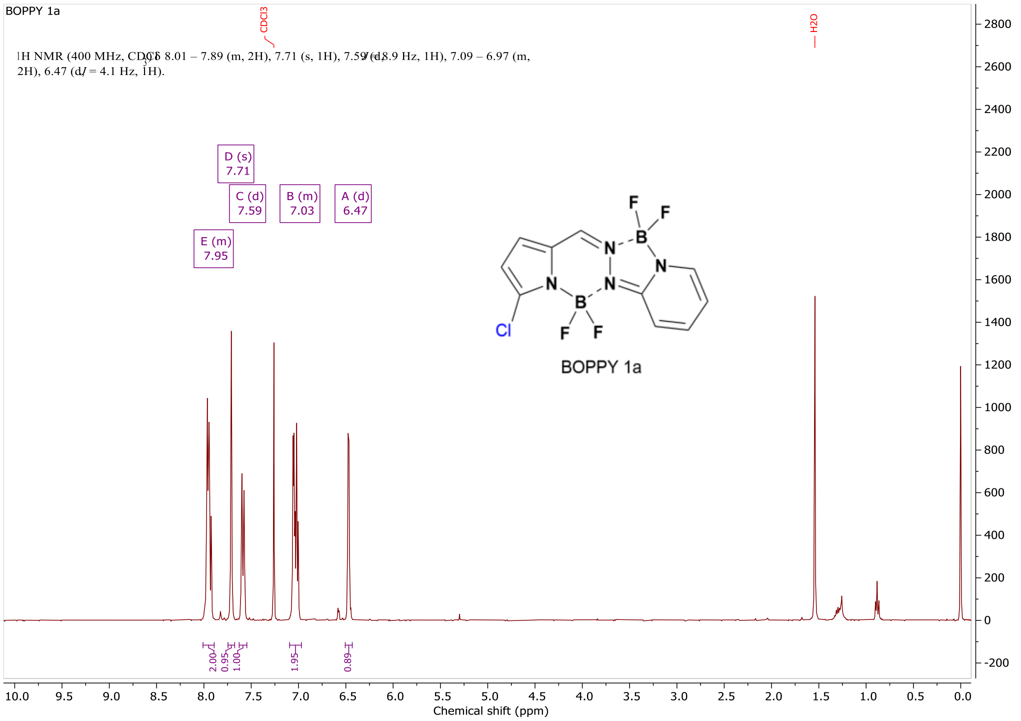

Figure S 16. ^{1}H NMR (400 MHz, $CDCl_3$) Spectrum

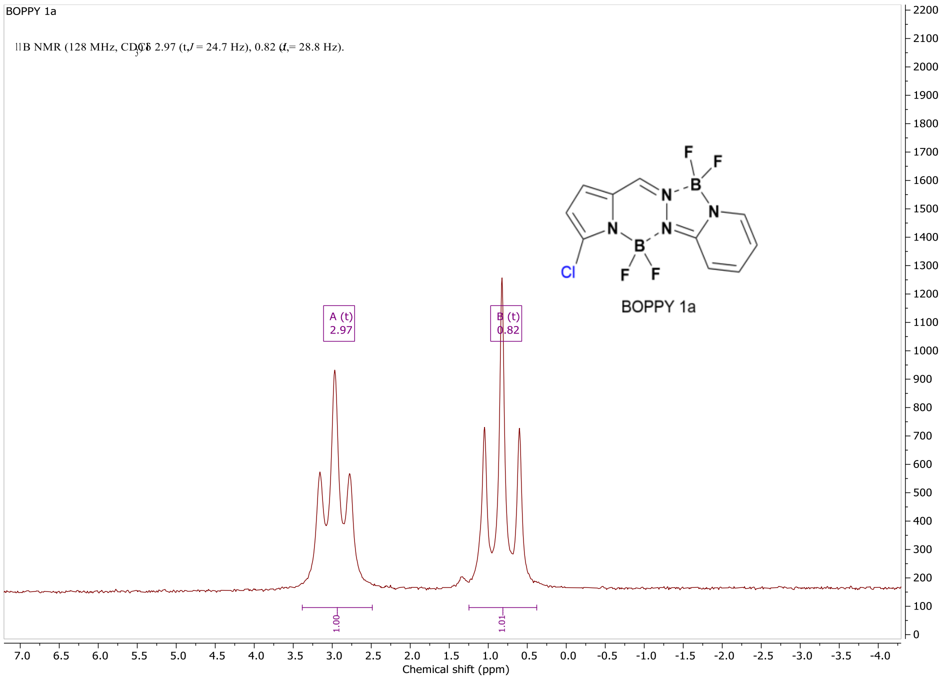

Figure S 17. ^{11}B NMR (128 MHz, $CDCl_3$) Spectrum

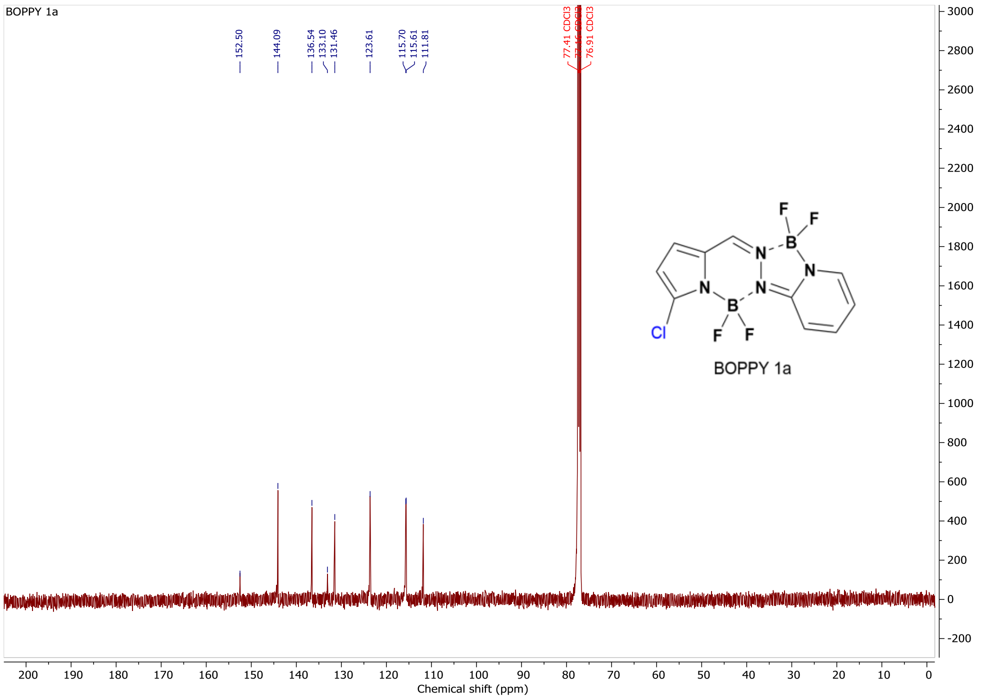

Figure S 18. $^{13}C\{1H\}$ **NMR (126 MHz, $CDCl_3$) Spectrum**

BOPPY 1b

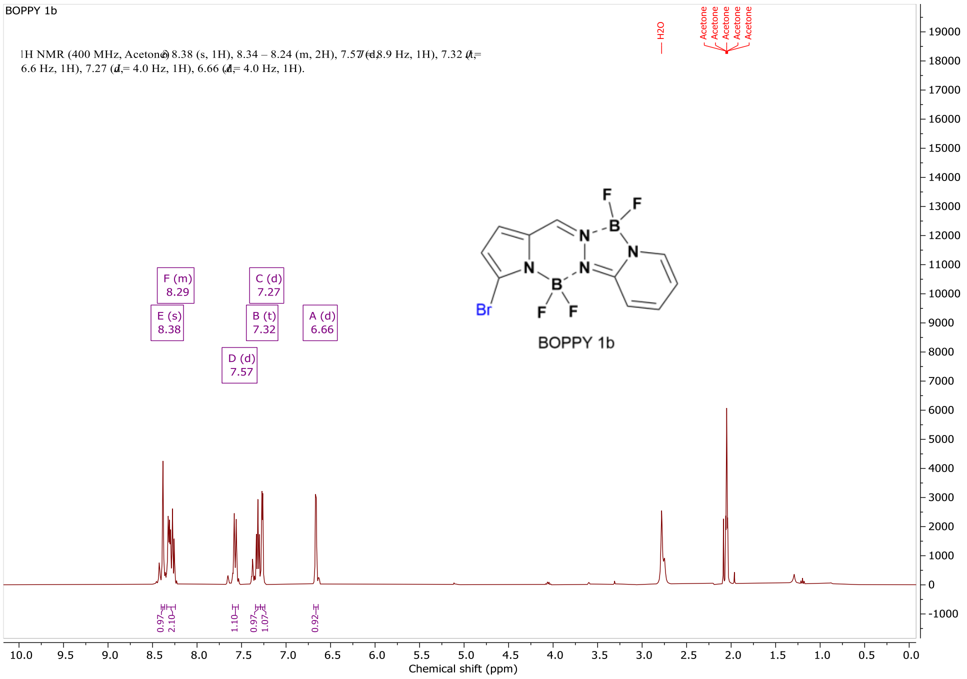

Figure S 19. ^{1}**H NMR (400 MHz, Acetone) Spectrum**

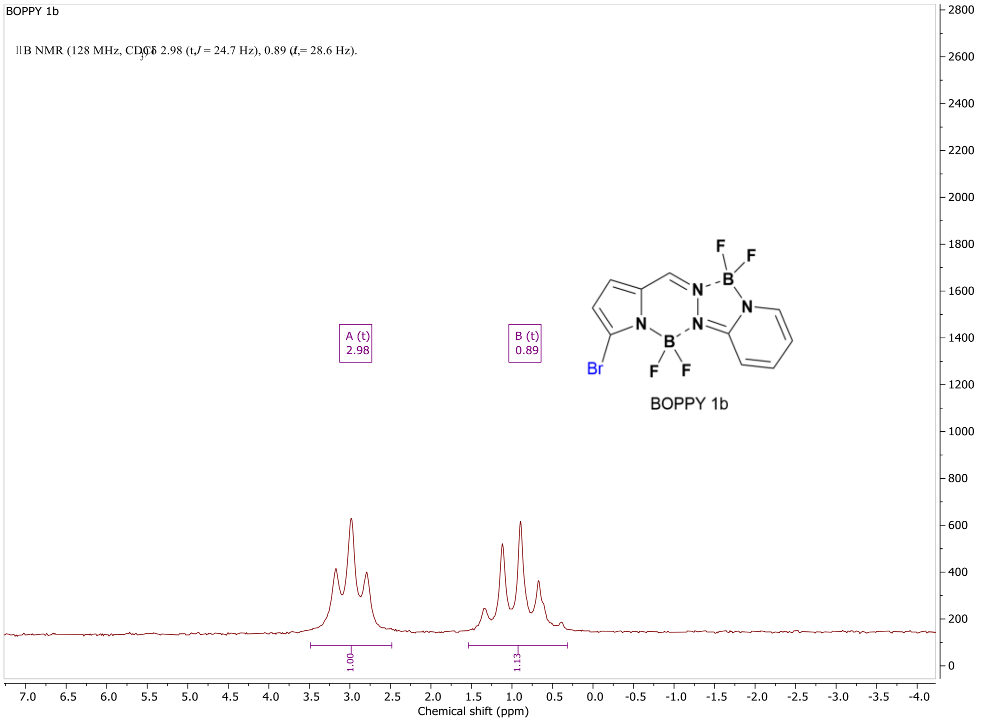

Figure S 20. ^{11}**B NMR (128 MHz,** $CDCl_3$**) Spectrum**

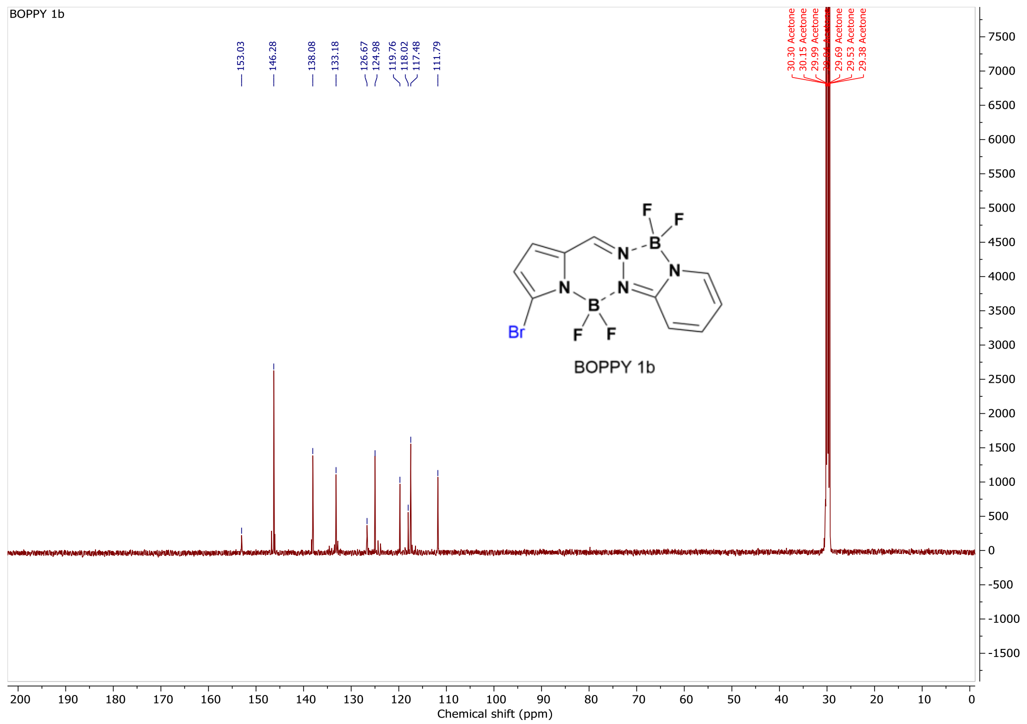

Figure S 21. $^{13}C\{1H\}$ **NMR (126 MHz, Acetone) Spectrum**

BOPPY 1c

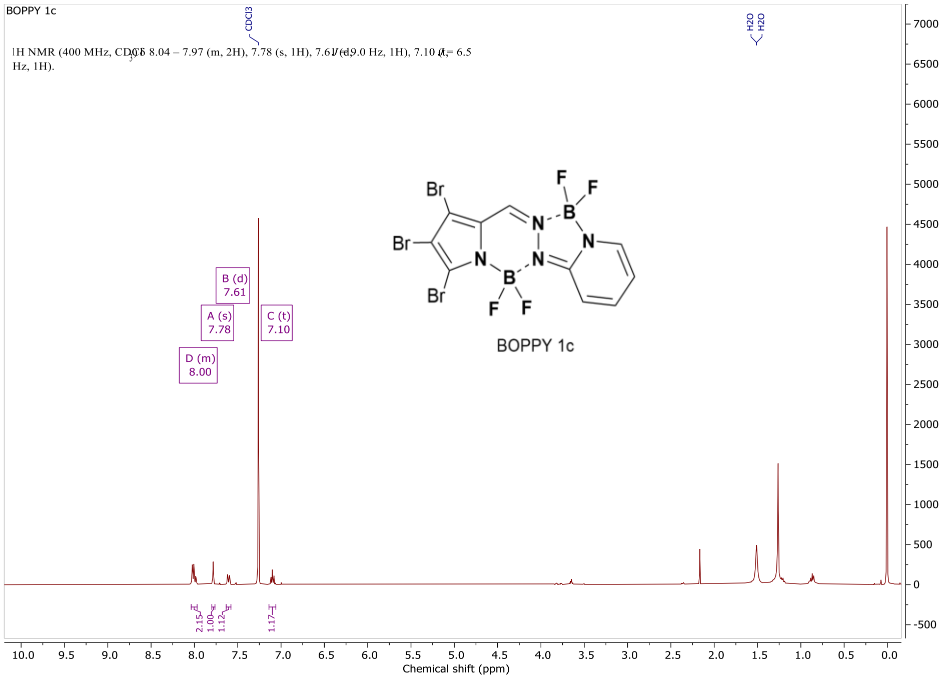

Figure S 22. ^{1}H NMR (400 MHz, CDCl$_3$) Spectrum

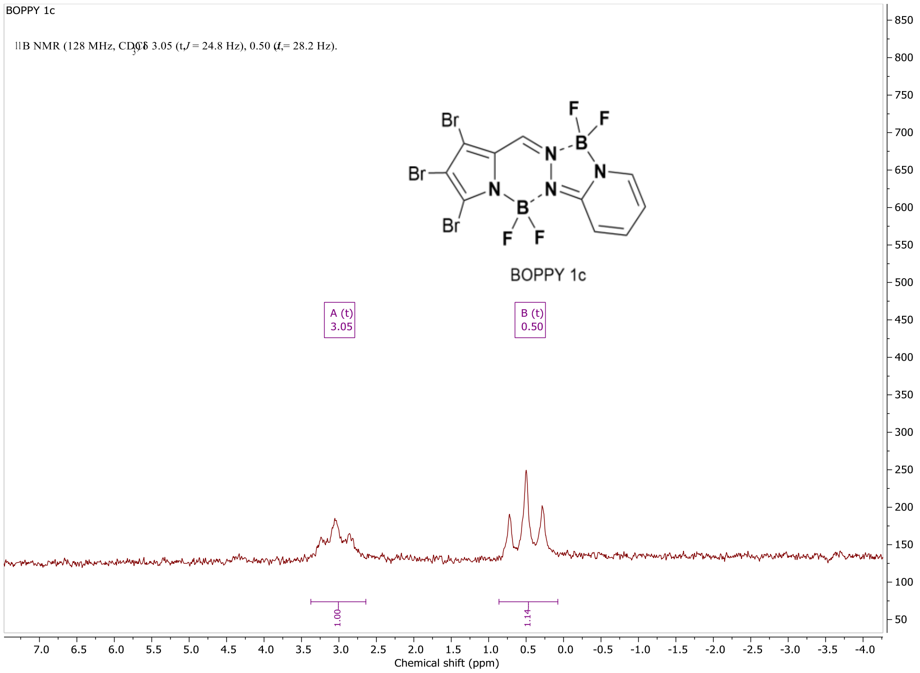

Figure S 23. ^{11}B NMR (128 MHz, CDCl$_3$*)* Spectrum

BOPPY 1ba

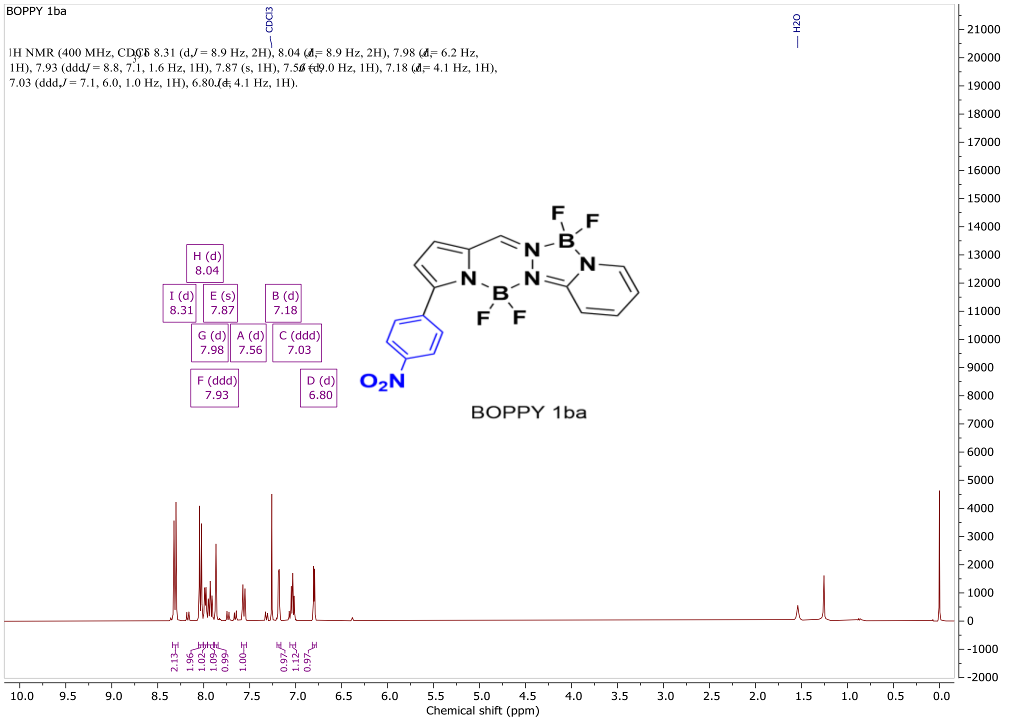

Figure S 24. **^{1}H NMR (400 MHz, $CDCl_3$) Spectrum**

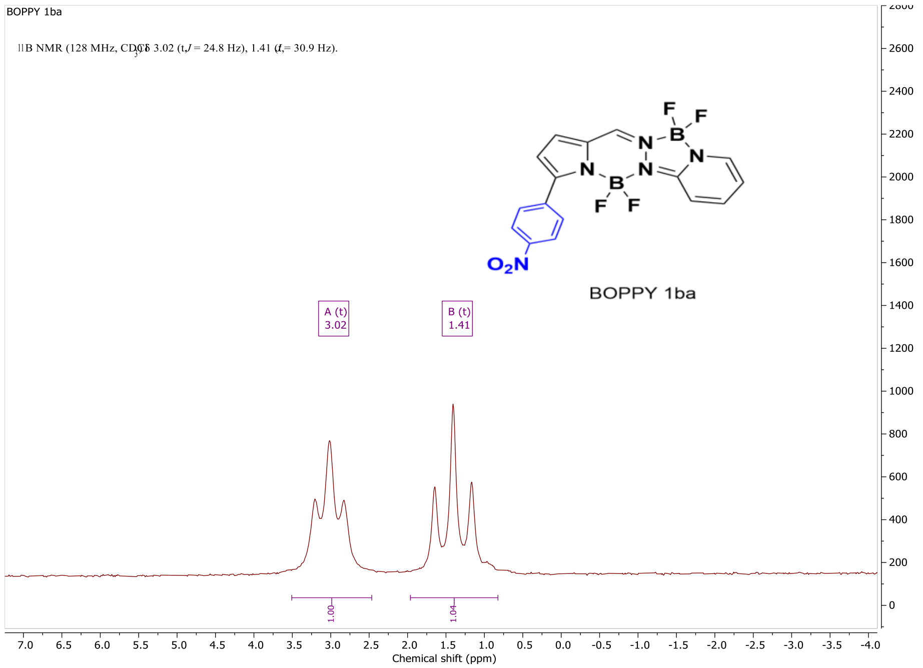

Figure S 25. ^{11}B NMR (128 MHz, $CDCl_3$) Spectrum

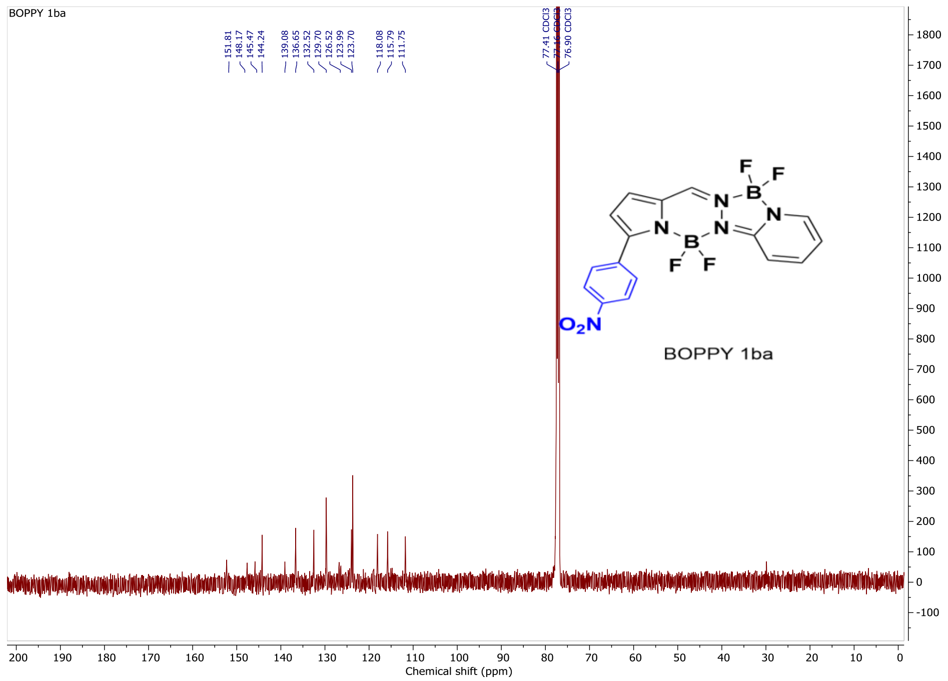

Figure S 26. $^{13}C\{1H\}$ NMR (126 MHz, $CDCl_3$) Spectrum

BOPPY 1bb

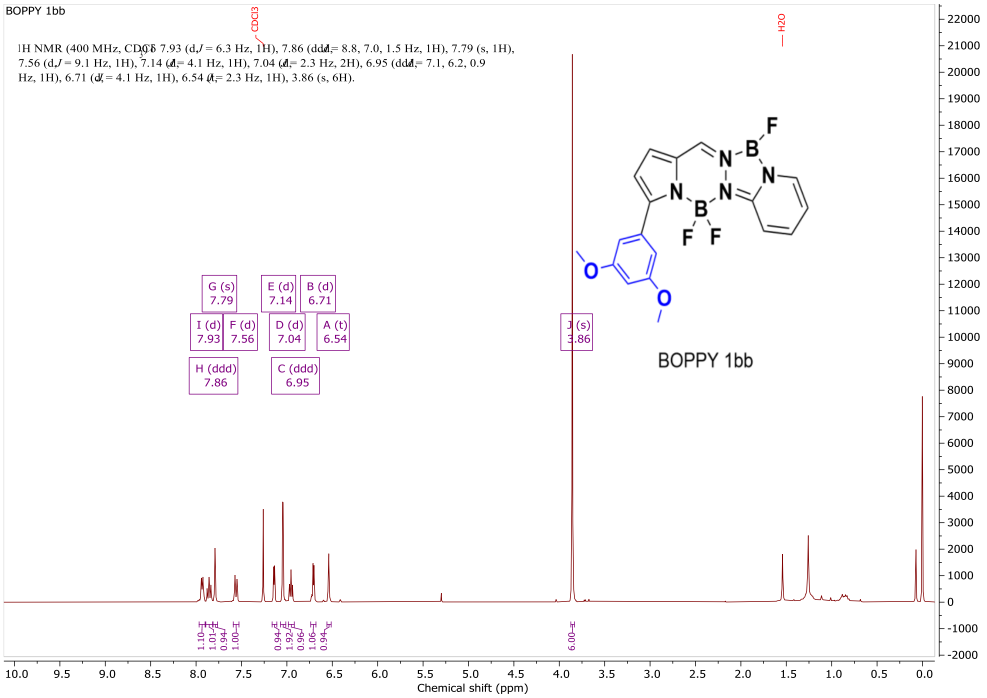

Figure S 27. ^{1}**H NMR (400 MHz, CDCl$_3$) Spectrum**

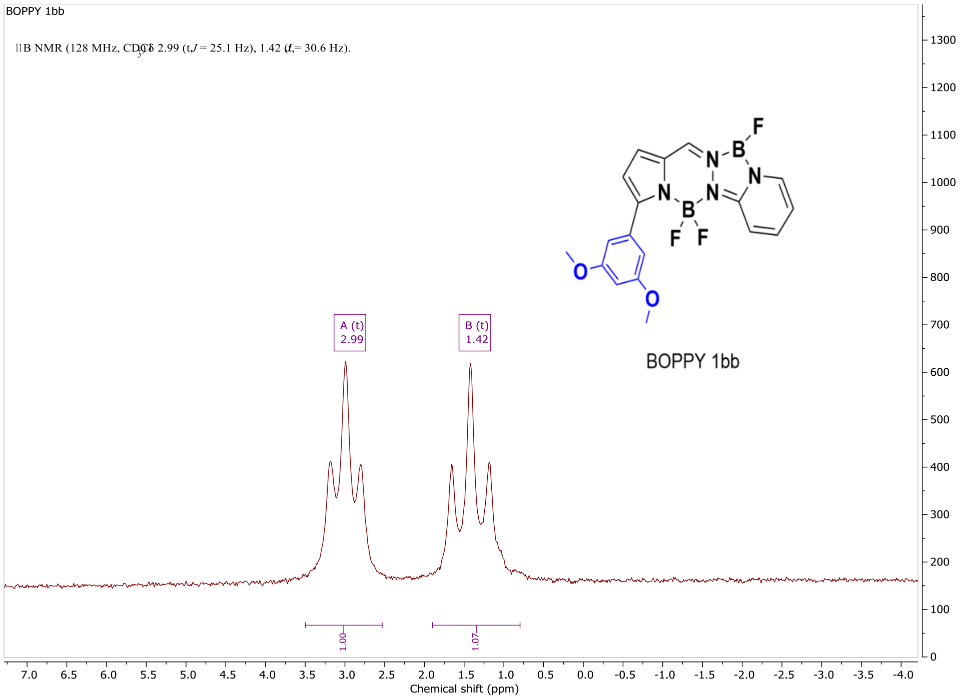

Figure S 28. ^{11}**B NMR (128 MHz, CDCl$_3$) Spectrum**

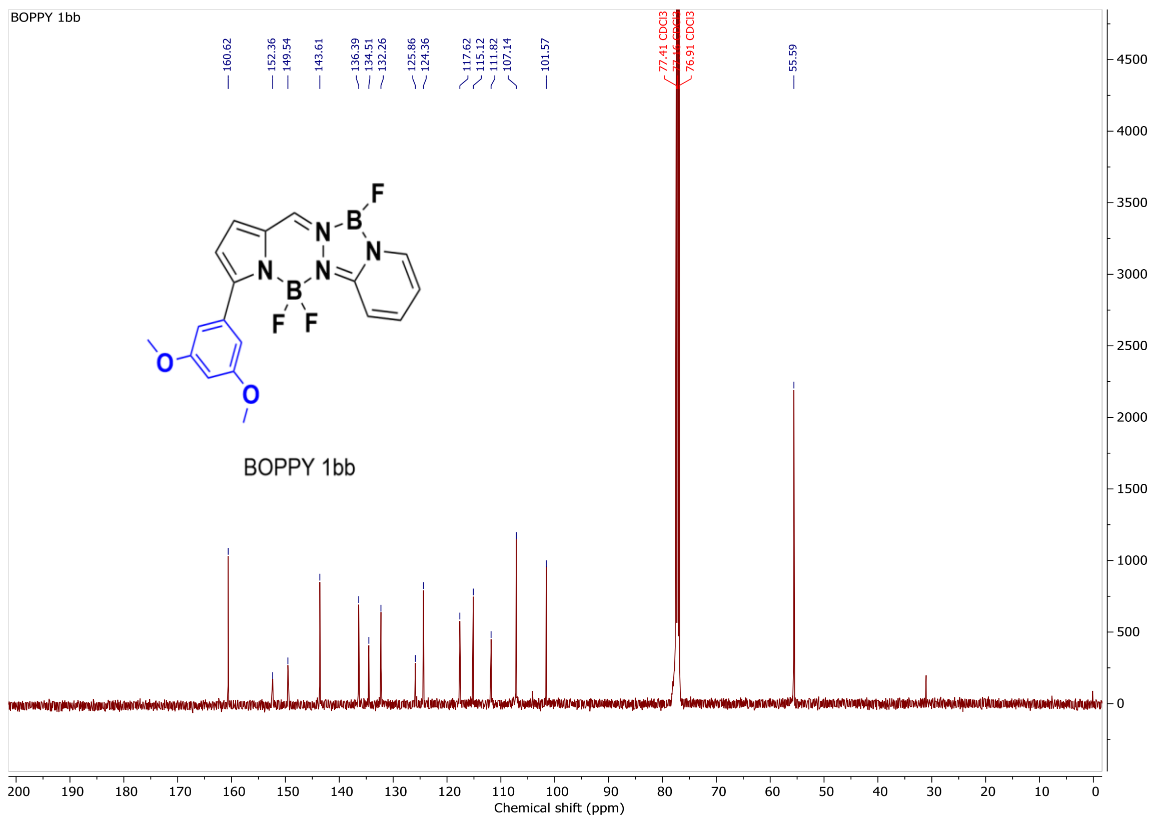

Figure S 29. $^{13}C\{1H\}$ **NMR (126 MHz,** $CDCl_3$**) Spectrum**

BOPPY 1bc

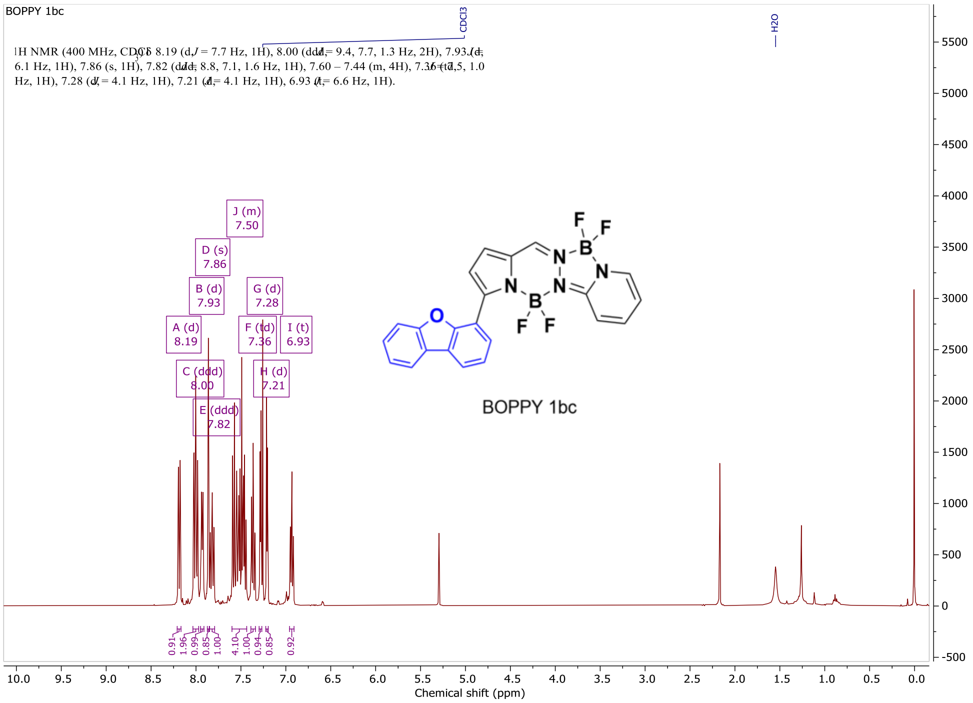

Figure S 30. ^{1}H NMR (400 MHz, $CDCl_3$) Spectrum

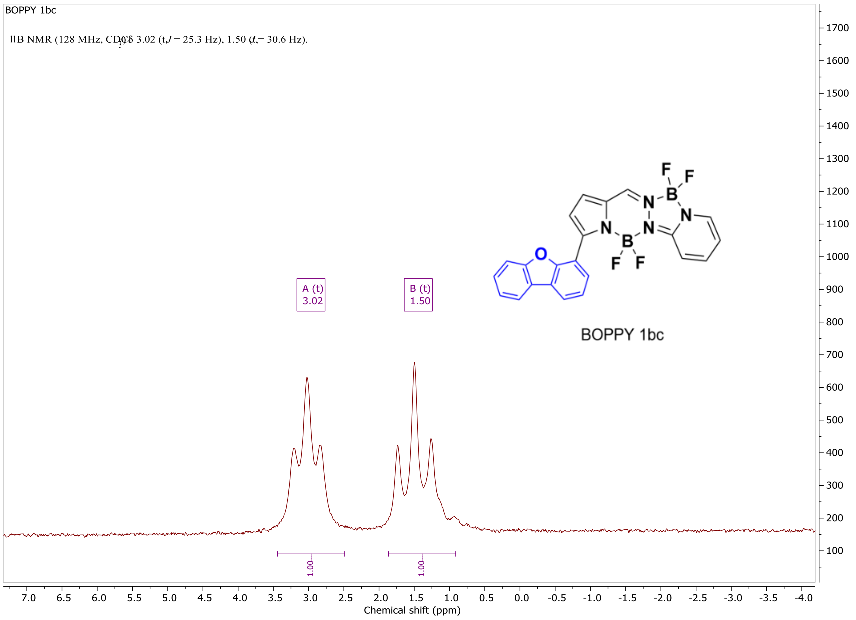

Figure S 31. ^{11}B NMR (128 MHz, $CDCl_3$) Spectrum

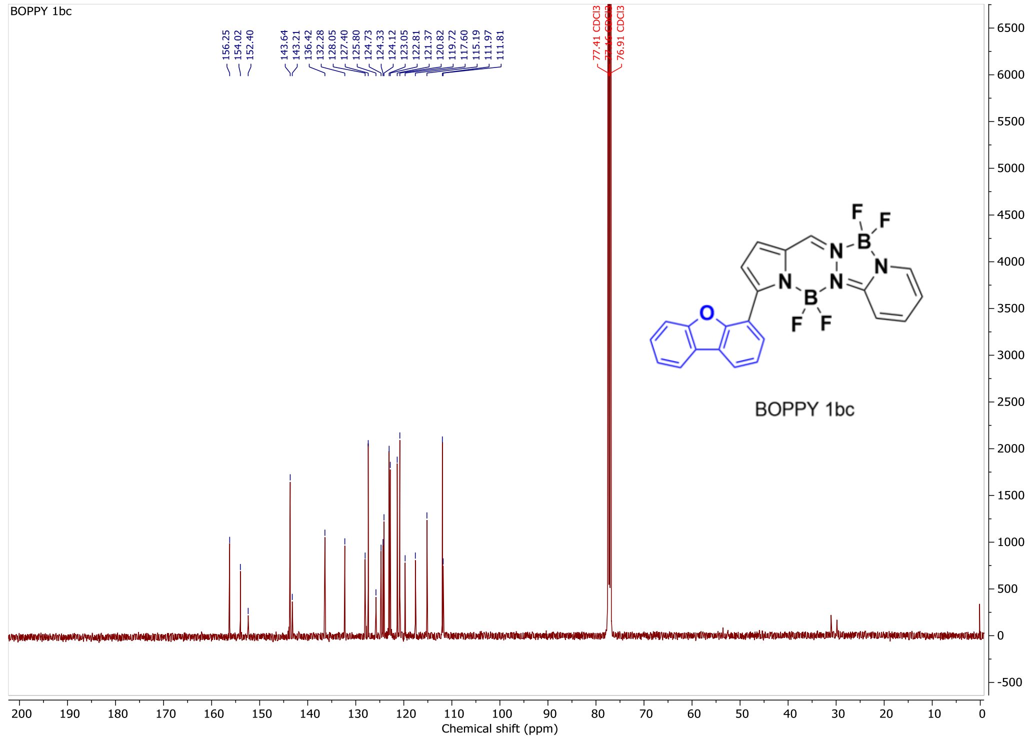

Figure S 32. $^{13}C\{^1H\}$ **NMR (126 MHz, $CDCl_3$) Spectrum**

BOPPY 1bd

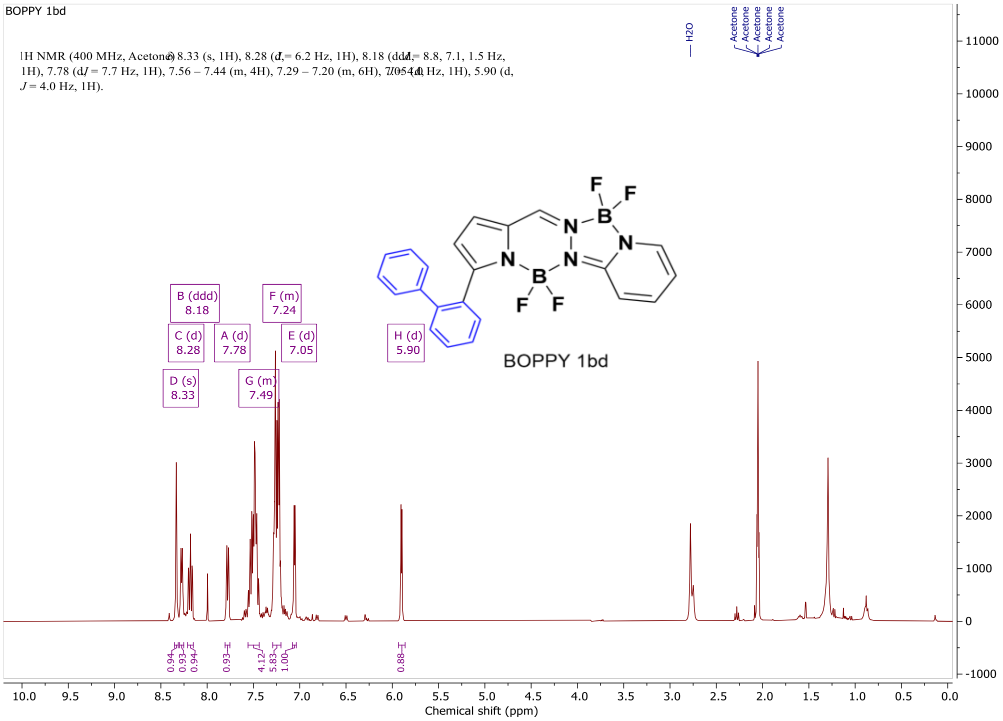

Figure S 33. ^{1}H NMR (400 MHz, Acetone) Spectrum

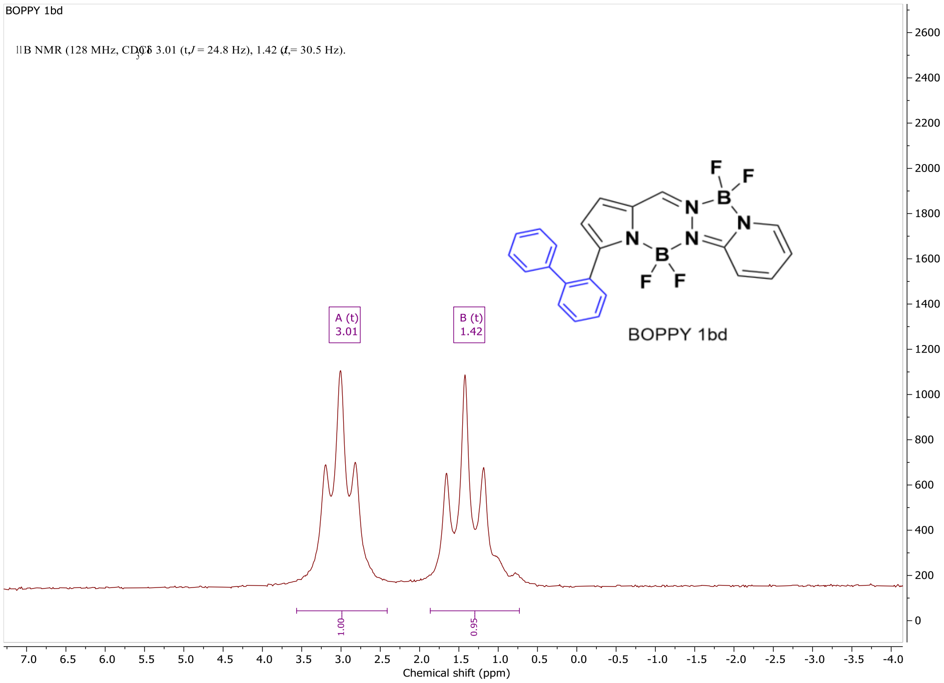

Figure S 34. ^{11}B NMR (128 MHz, $CDCl_3$) Spectrum

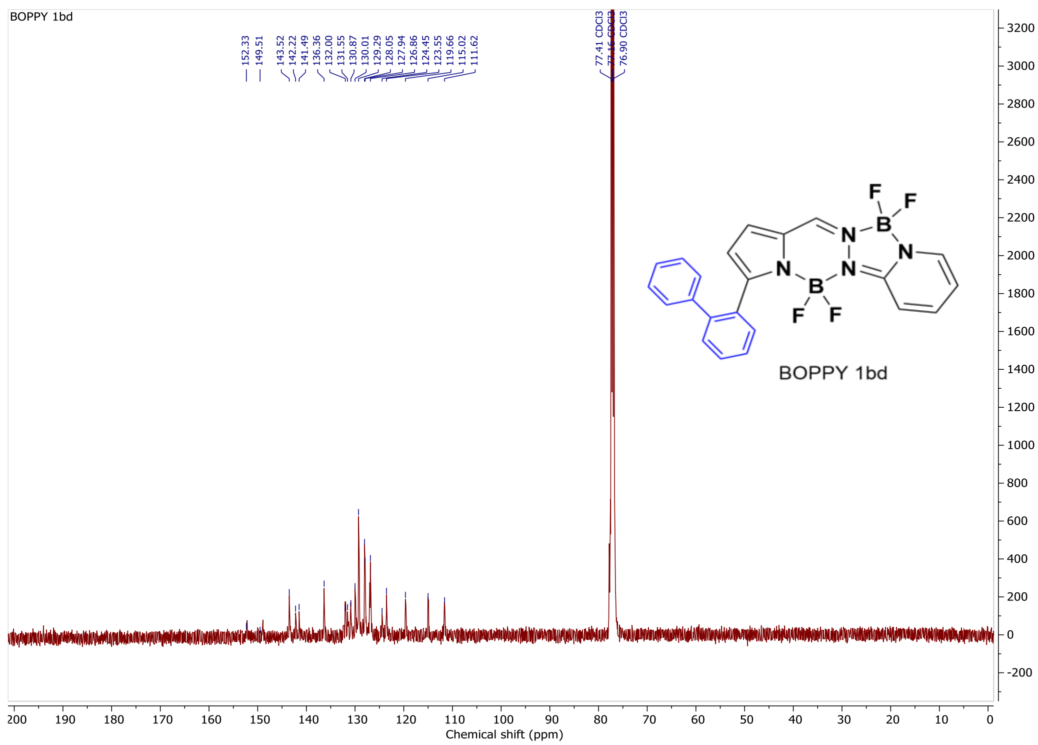

Figure S 35. ^{13}**C{1H} NMR (126 MHz,** $CDCl_3$**) Spectrum**

BOPPY 1be

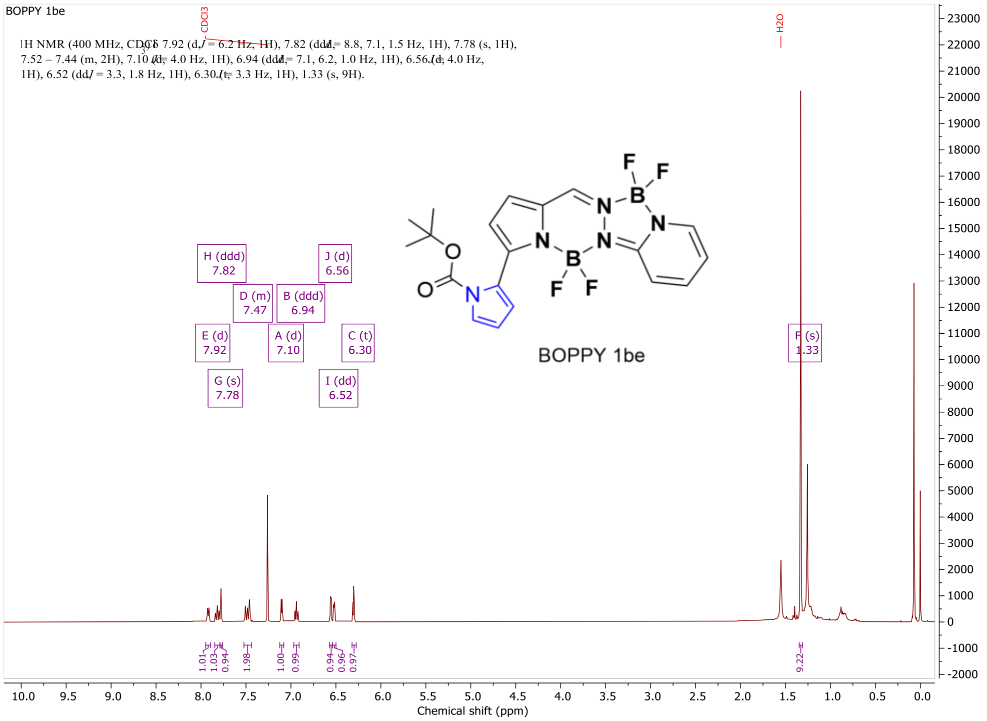

Figure S 36. ^{1}H NMR (400 MHz, CDCl$_3$) Spectrum

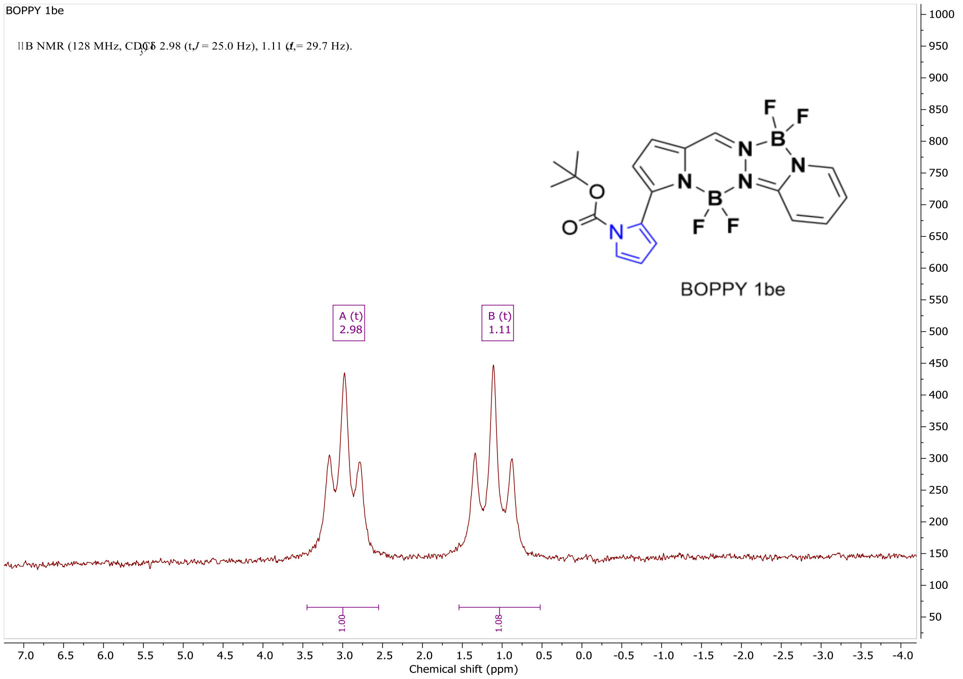

Figure S 37. ^{11}B NMR (128 MHz, CDCl$_3$) Spectrum

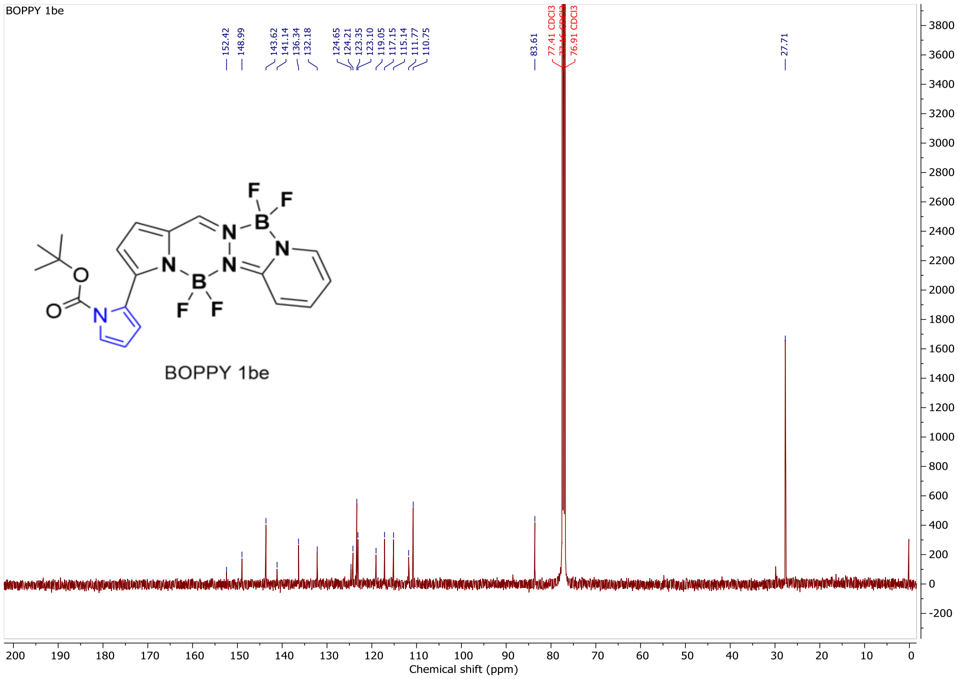

Figure S 38. $^{13}C\{1H\}$ **NMR (126 MHz,** $CDCl_3$**) Spectrum**

BOPPY 1be’

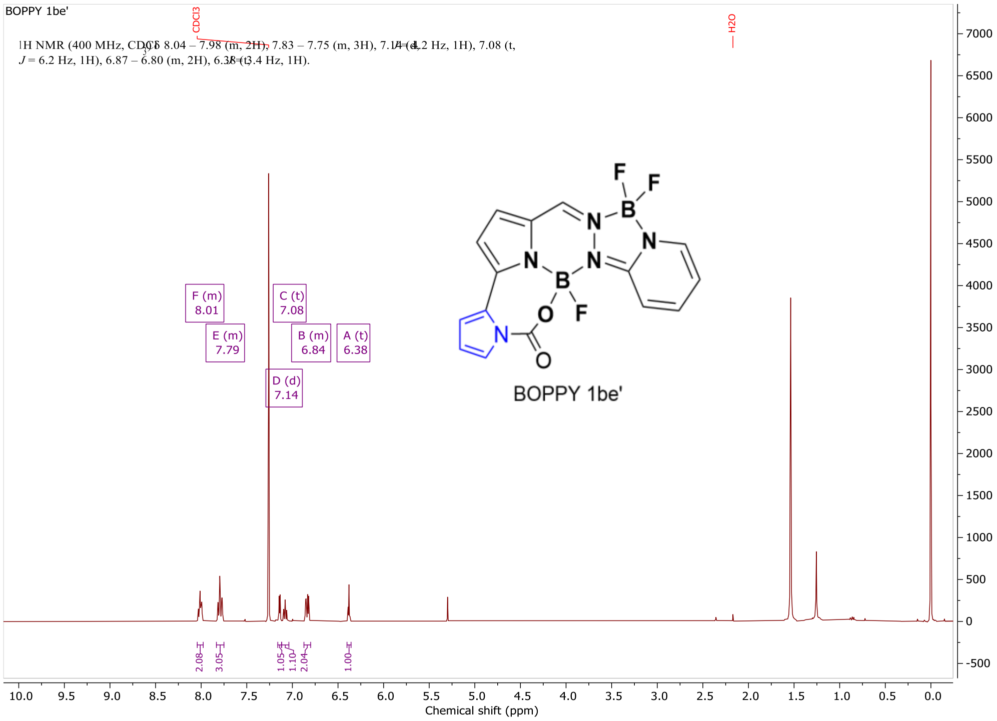

Figure S 39. ^{1}H NMR (400 MHz, $CDCl_3$) Spectrum

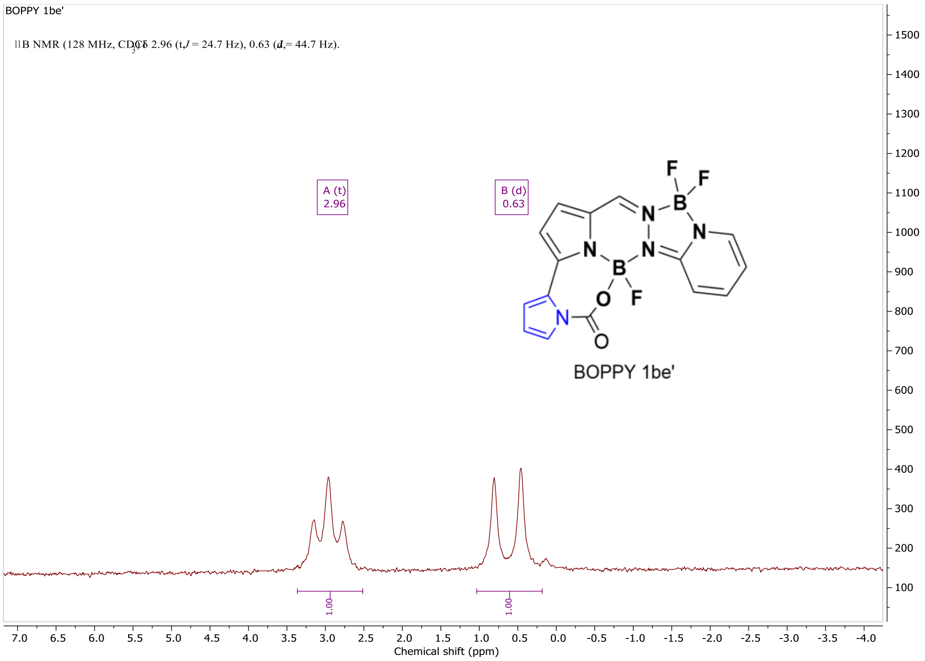

Figure S 40. ^{11}B NMR (128 MHz, $CDCl_3$) Spectrum

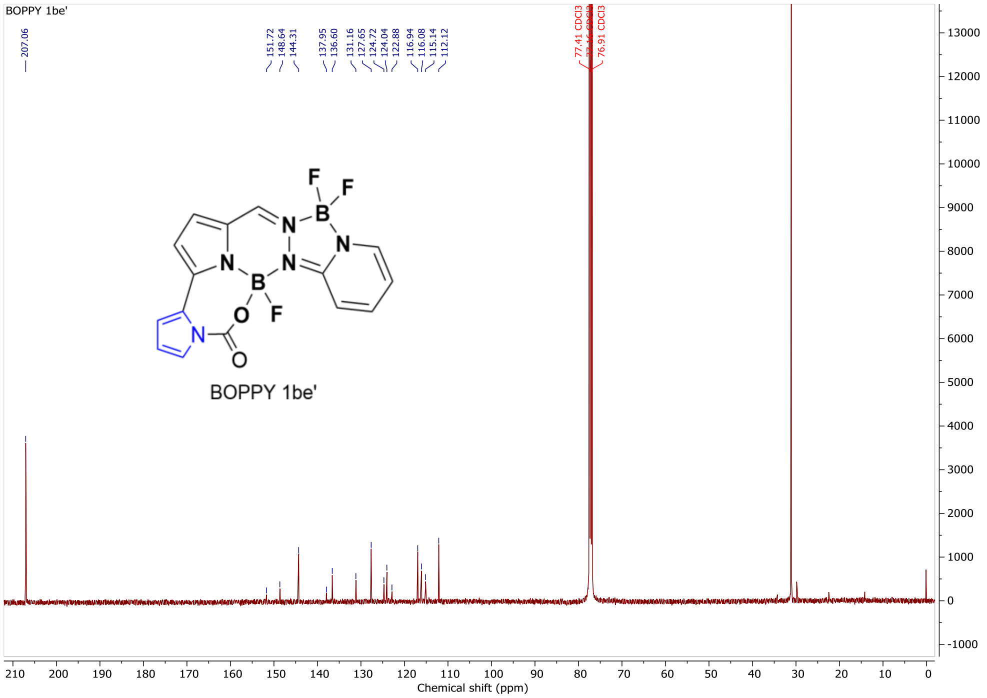

Figure S 41. $^{13}C\{1H\}$ NMR (126 MHz, $CDCl_3$) Spectrum

BOPPY 1bf

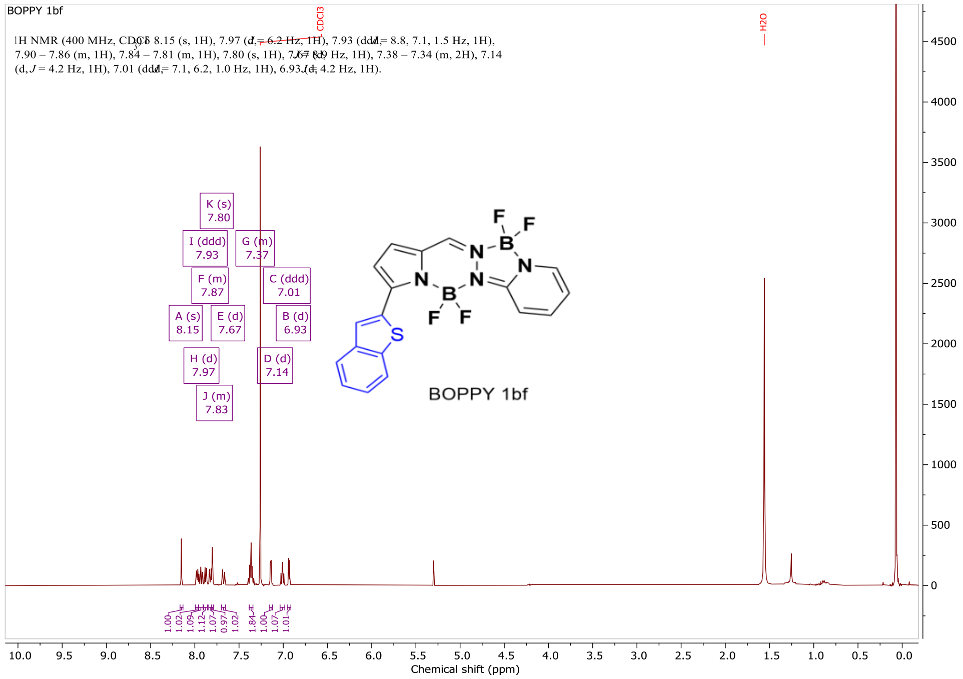

Figure S 42. ^{1}H NMR (400 MHz, $CDCl_3$) Spectrum

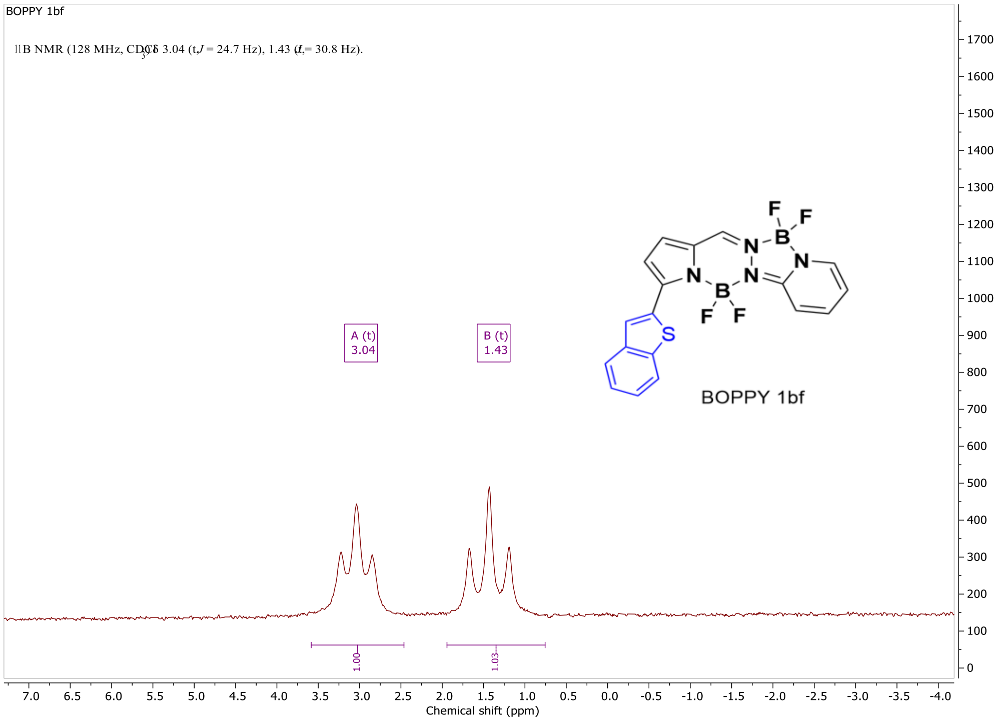

Figure S 43. ^{11}B NMR (128 MHz, $CDCl_3$) Spectrum

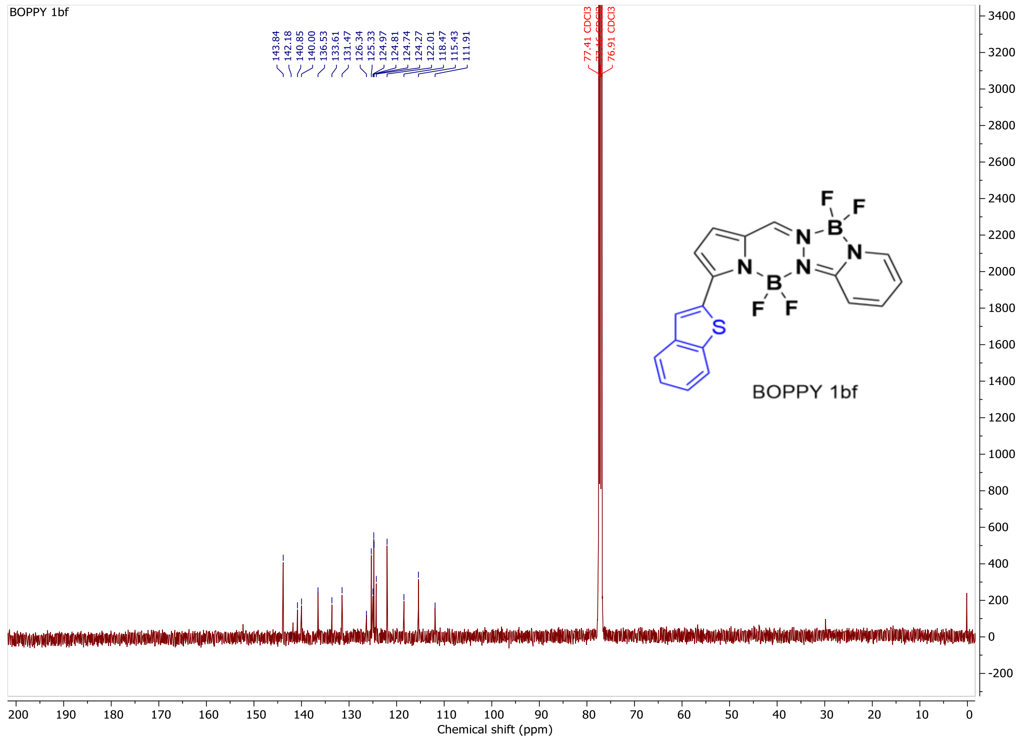

Figure S 44. $^{13}C\{1H\}$ NMR (126 MHz, $CDCl_3$) Spectrum

BOPPY 1bg

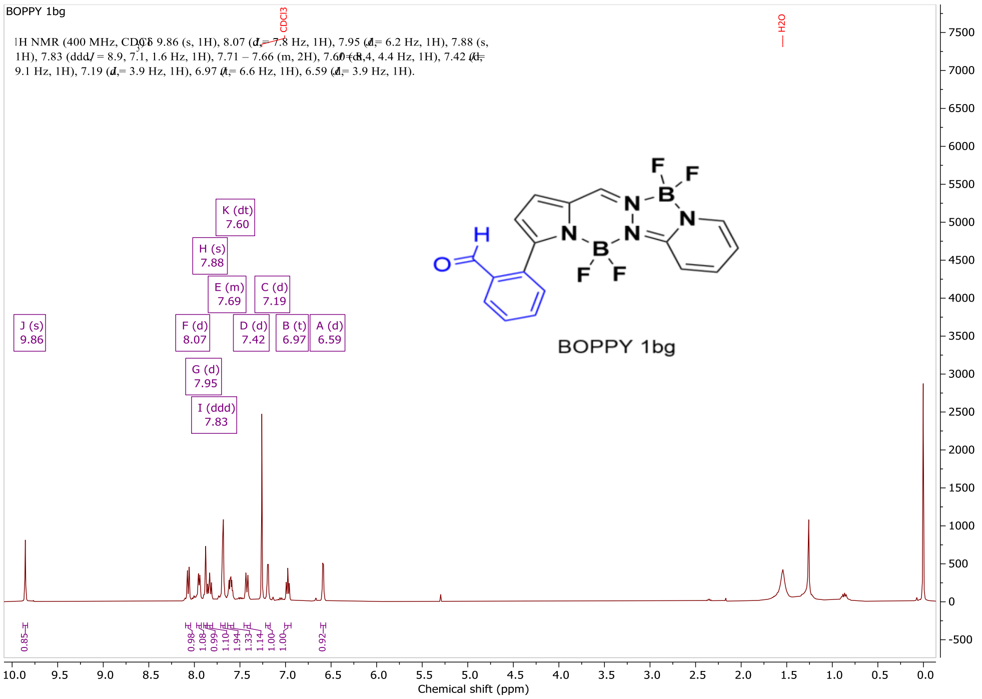

Figure S 45. ^{1}H NMR (400 MHz, CDCl$_3$) Spectrum

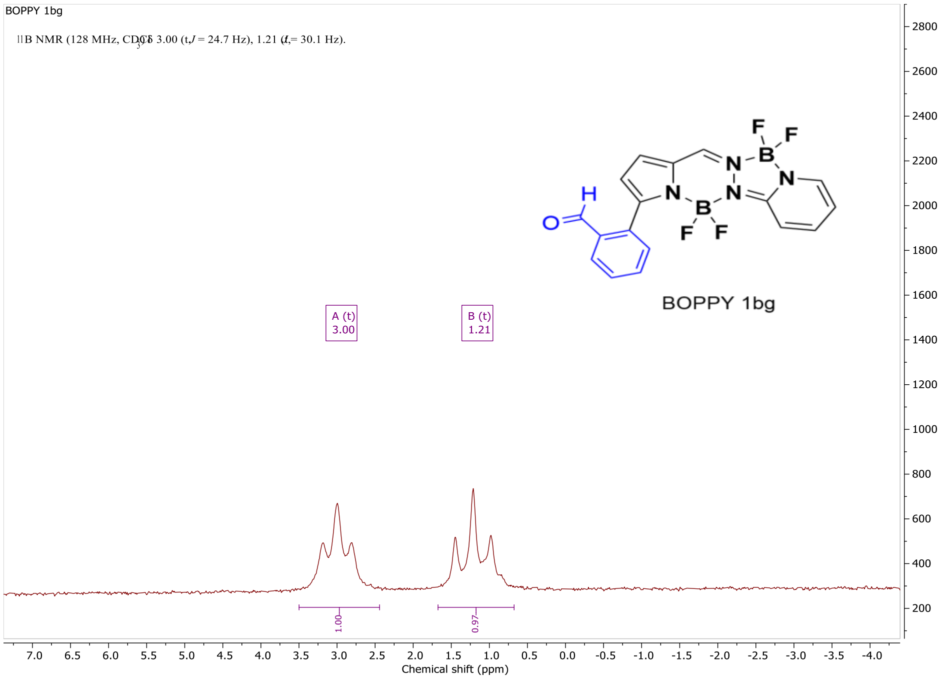

Figure S 46. ^{11}B NMR (128 MHz, CDCl$_3$) Spectrum

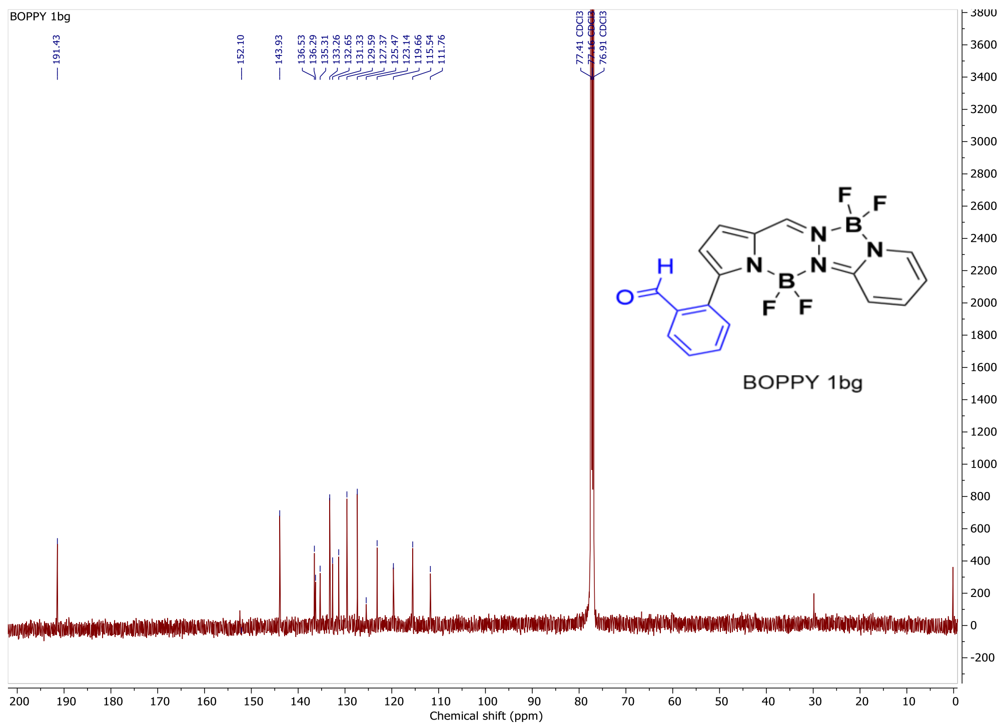

Figure S 47. ^{13}**C{1H} NMR (126 MHz, CDCl$_3$) Spectrum**

BOPPY 1bh

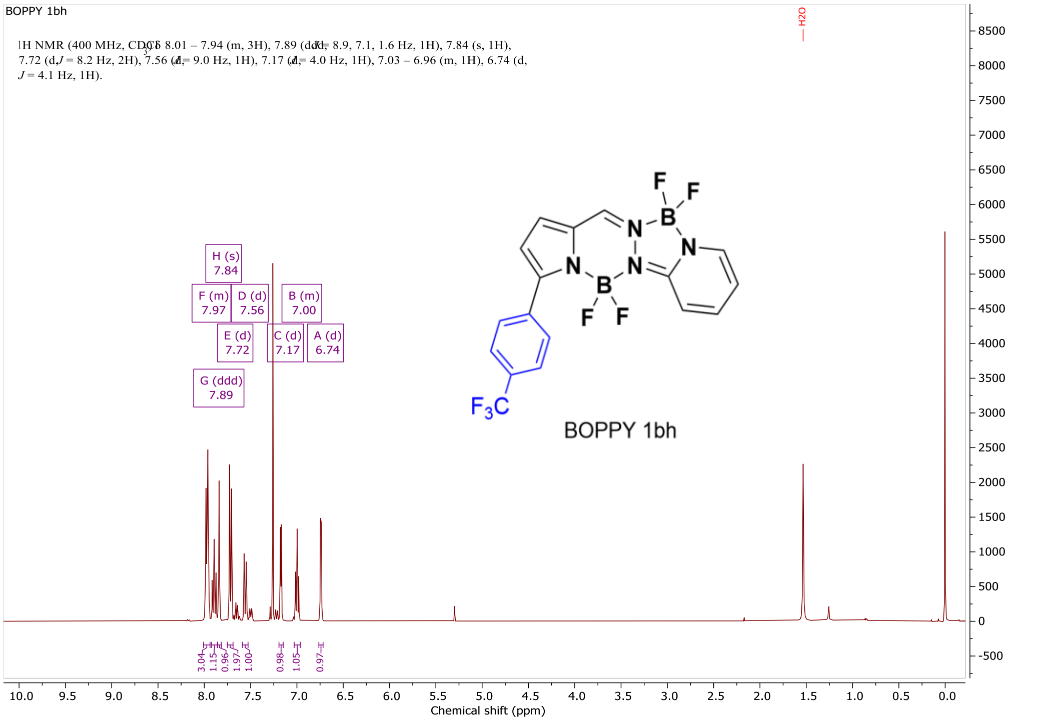

Figure S 48. ^{1}**H NMR (400 MHz, CDCl$_3$) Spectrum**

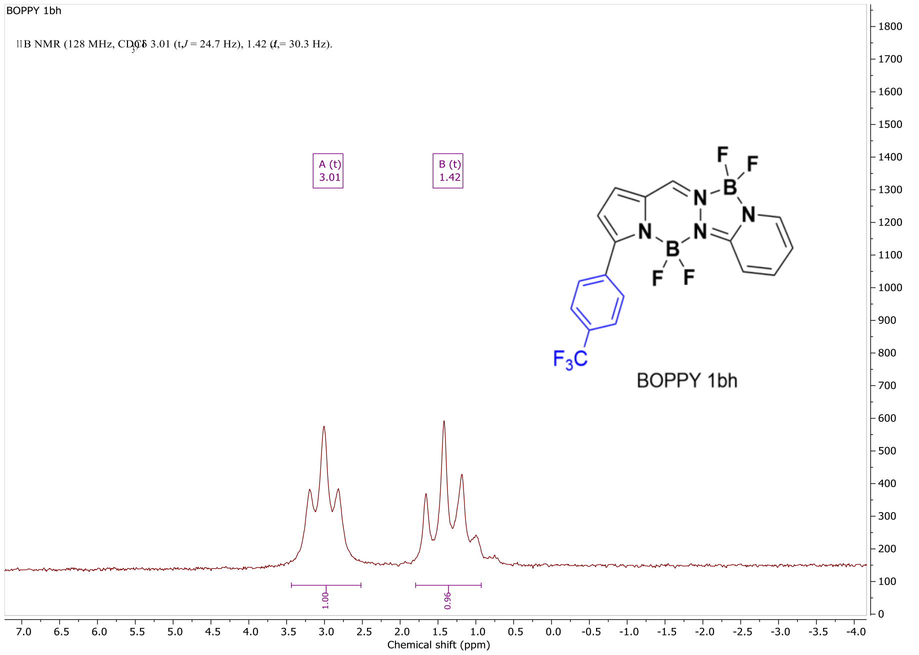

Figure S 49. ^{11}**B NMR (128 MHz, CDCl$_3$) Spectrum**

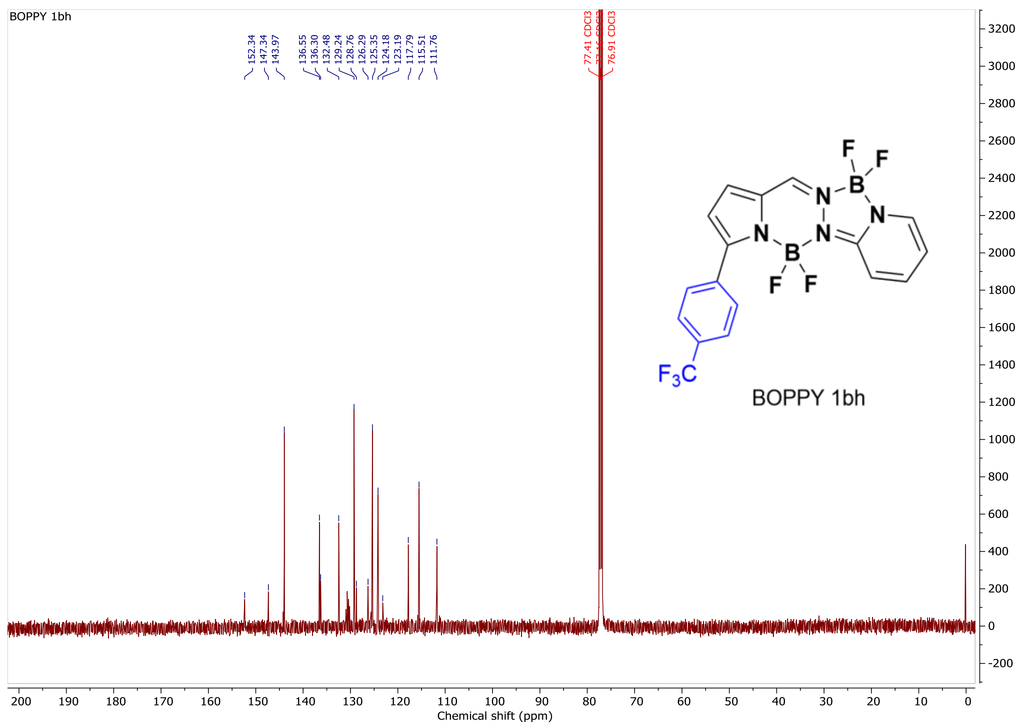

Figure S 50. ^{13}C{1H} NMR (126 MHz, CDCl$_3$) Spectrum

BOPPY 1bi

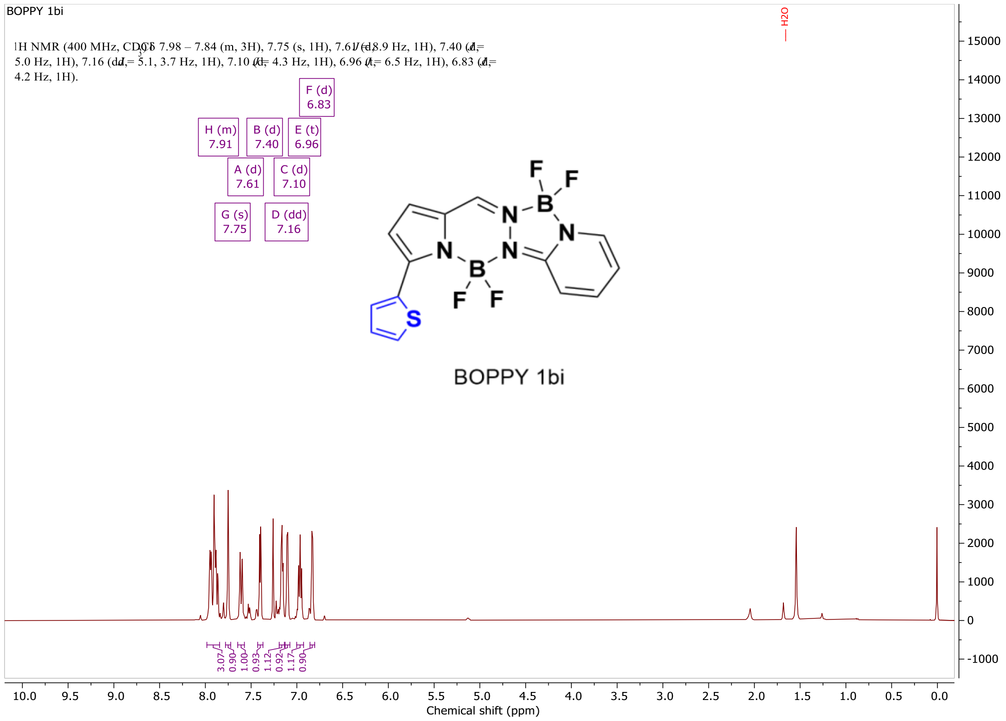

Figure S 51. ^{1}H NMR (400 MHz, $CDCl_3$) Spectrum

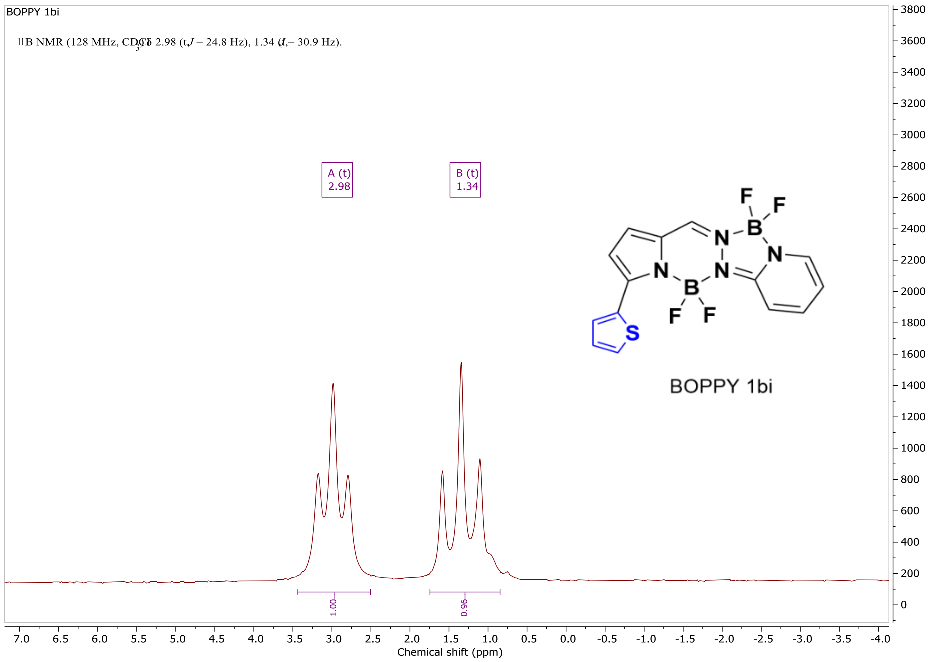

Figure S 52. ^{11}B NMR (128 MHz, $CDCl_3$) Spectrum

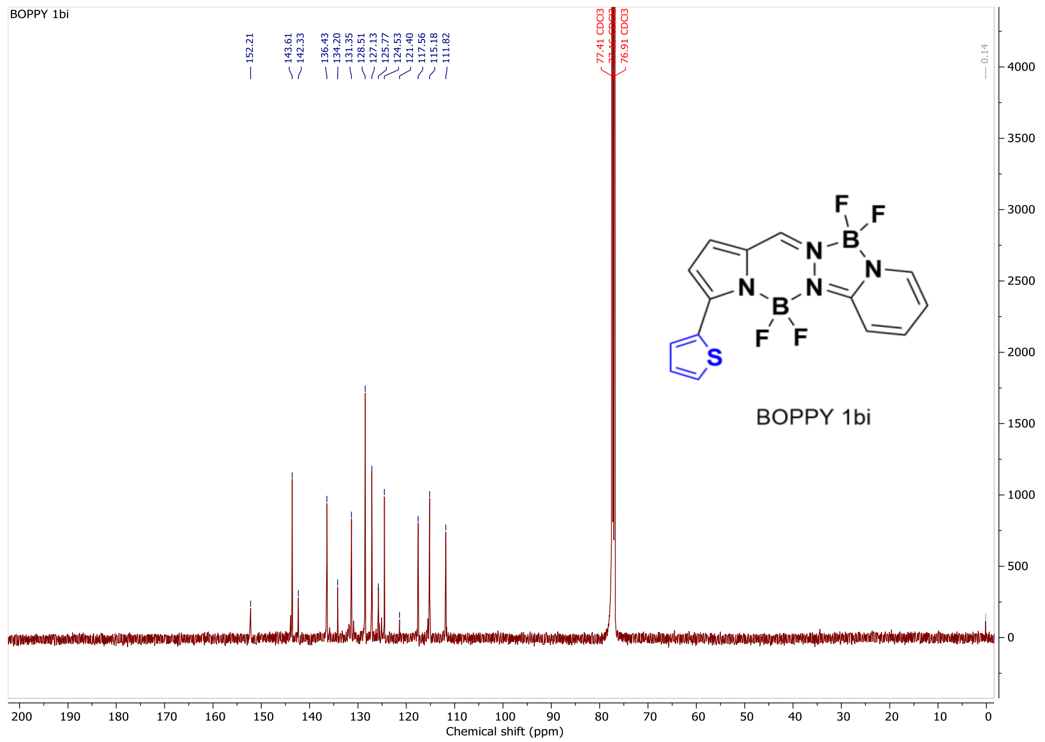

Figure S 53. $^{13}C\{1H\}$ **NMR (126 MHz,** $CDCl_3$**) Spectrum**

BOPPY 1bj

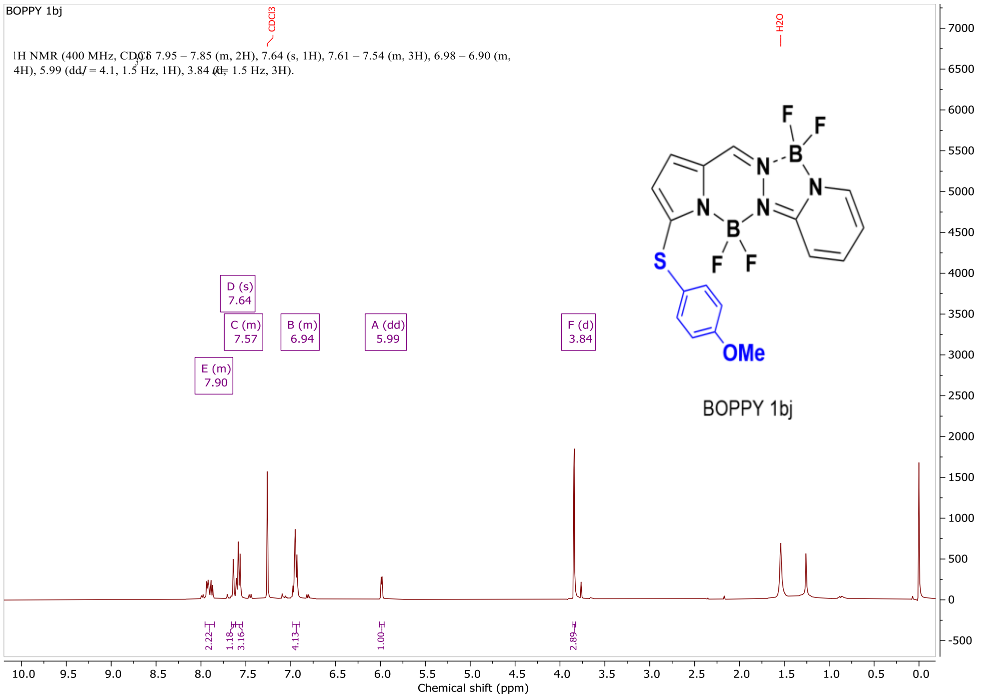

Figure S 54. ^{1}**H NMR (400 MHz, CDCl$_3$) Spectrum**

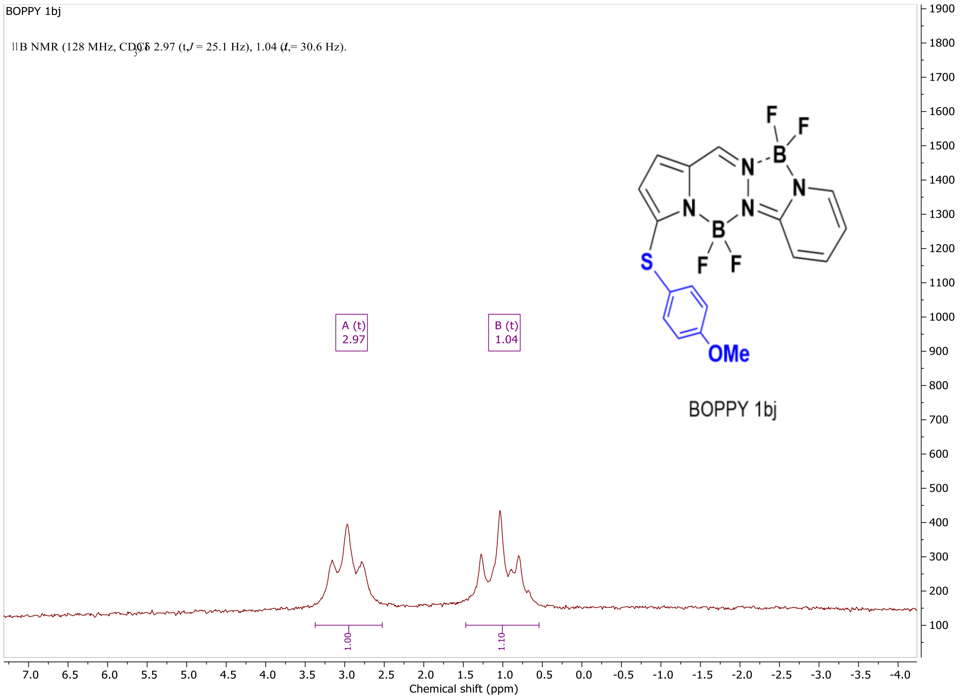

Figure S 55. ^{11}**B NMR (128 MHz, CDCl$_3$) Spectrum**

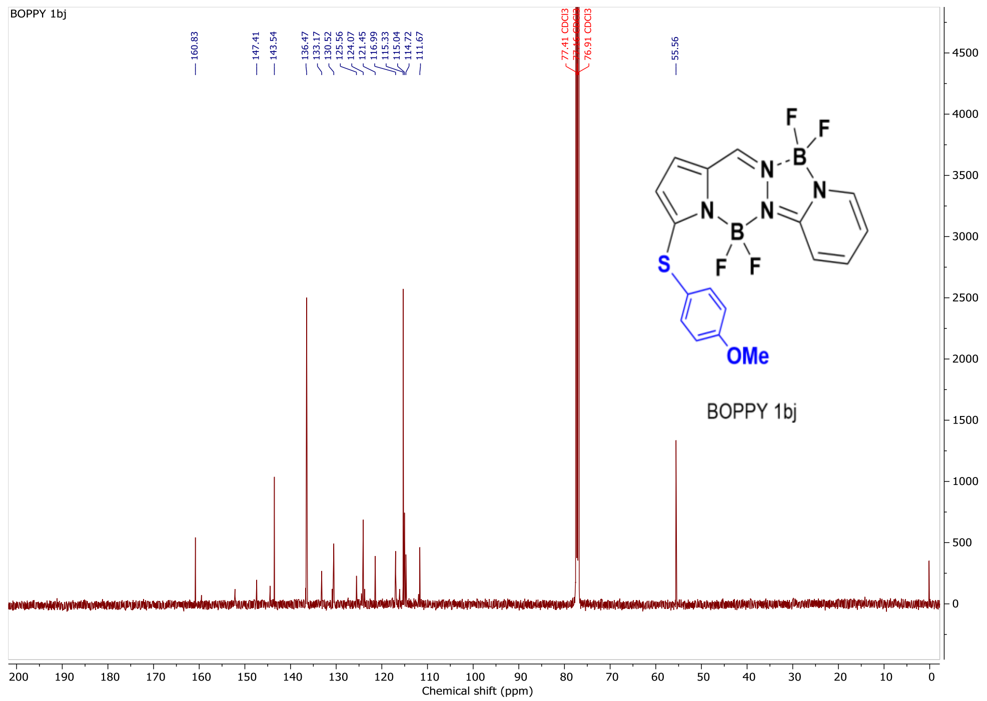

Figure S 56. $^{13}C\{1H\}$ **NMR (126 MHz,** $CDCl_3$**) Spectrum**

BOPPY 1ca

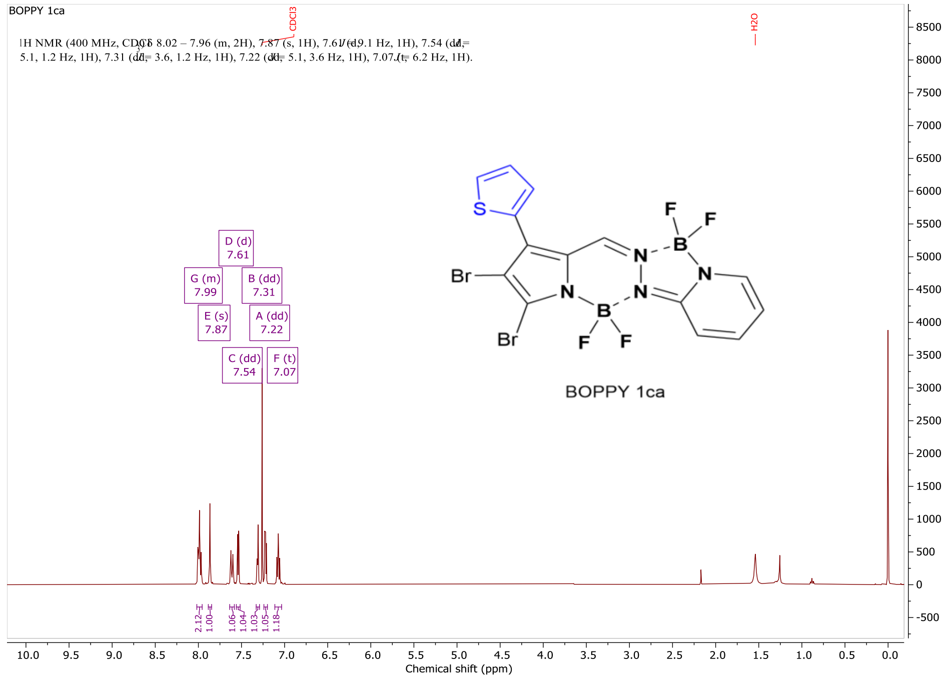

Figure S 57. ^{1}**H NMR (400 MHz, CDCl$_3$) Spectrum**

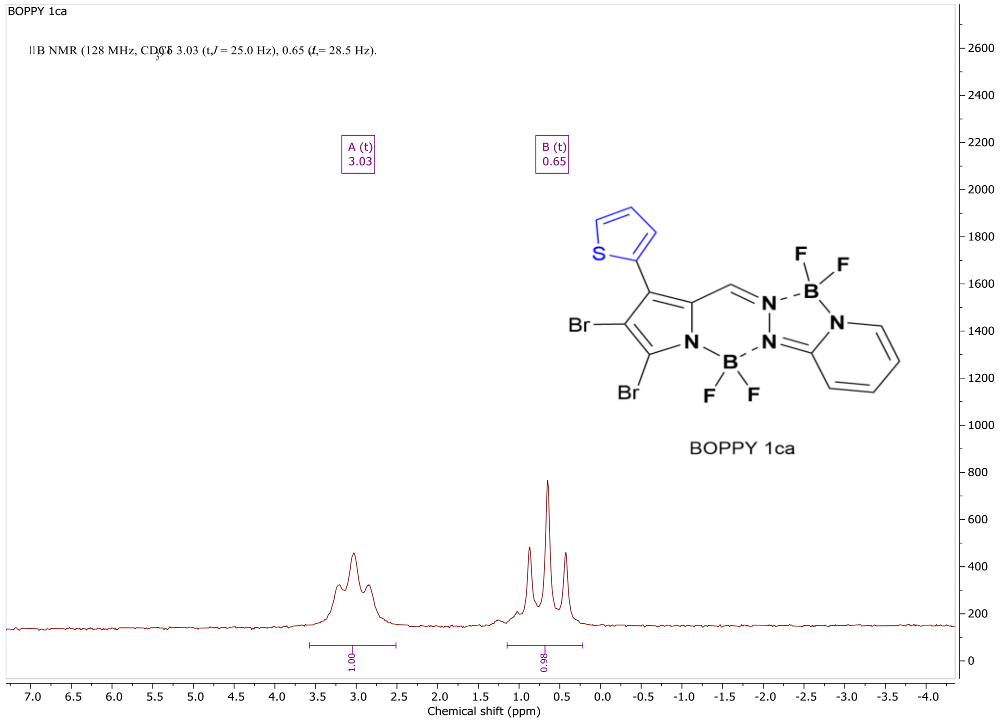

Figure S 58. ^{11}**B NMR (128 MHz, CDCl$_3$) Spectrum**

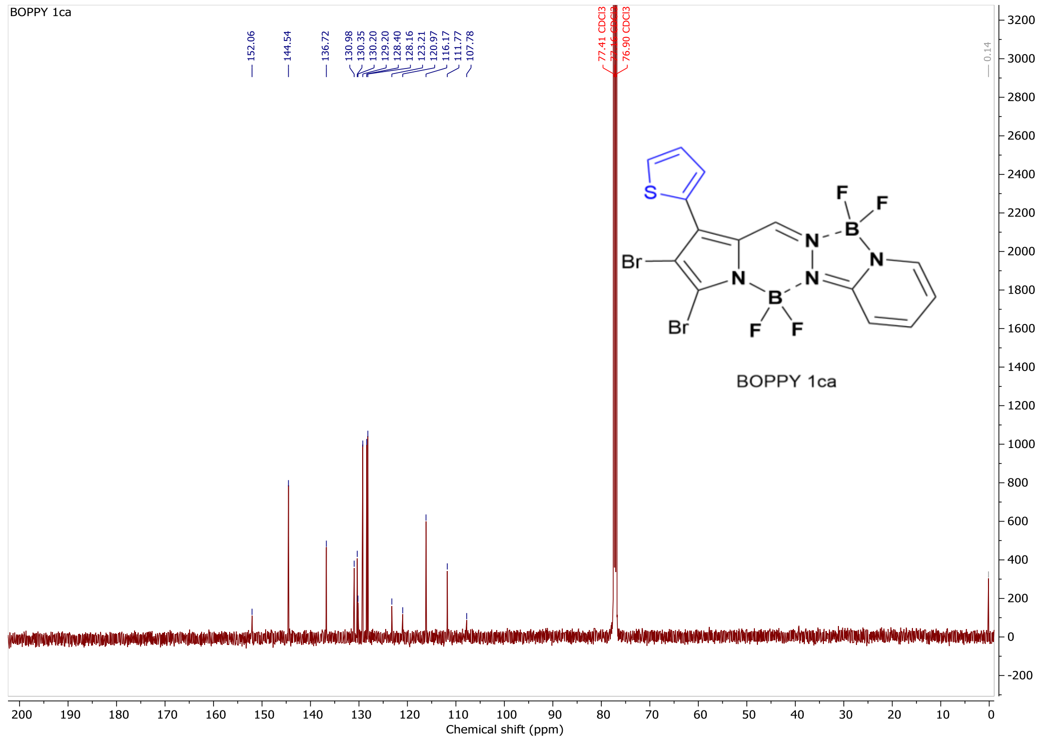

Figure S 59. $^{13}C\{^1H\}$ **NMR (126 MHz,** $CDCl_3$**) Spectrum**

BOPPY 1cb

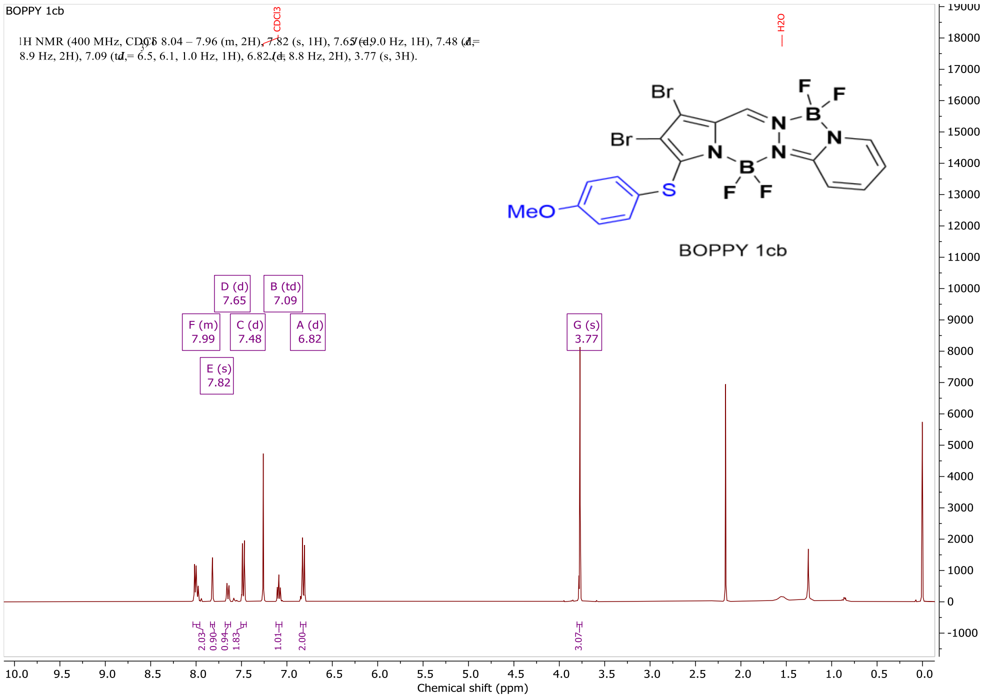

Figure S 60. ^{1}H NMR (400 MHz, $CDCl_3$) Spectrum

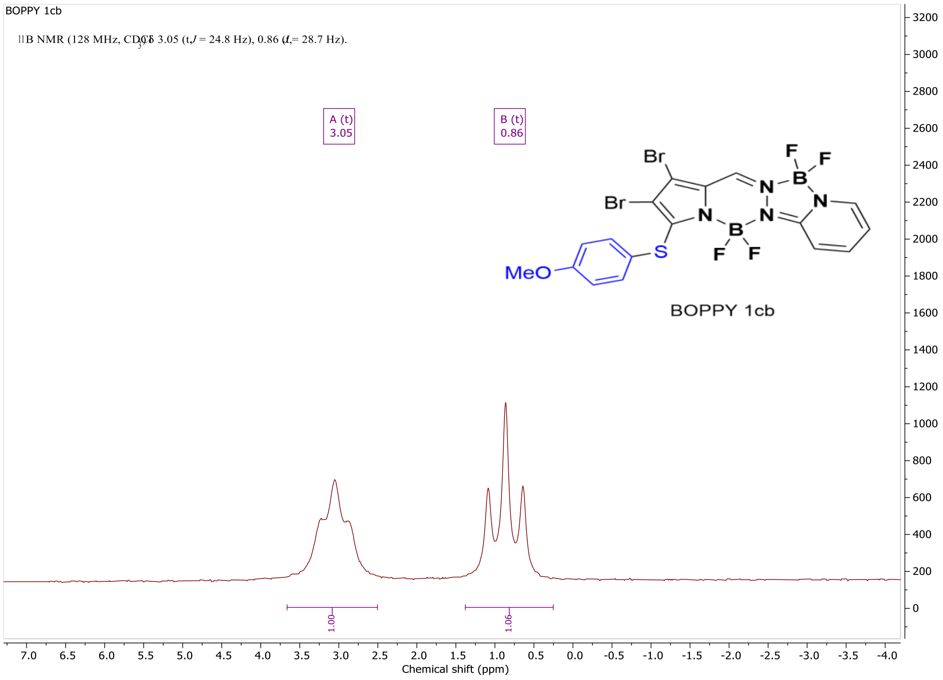

Figure S 61. ^{11}B NMR (128 MHz, $CDCl_3$) Spectrum

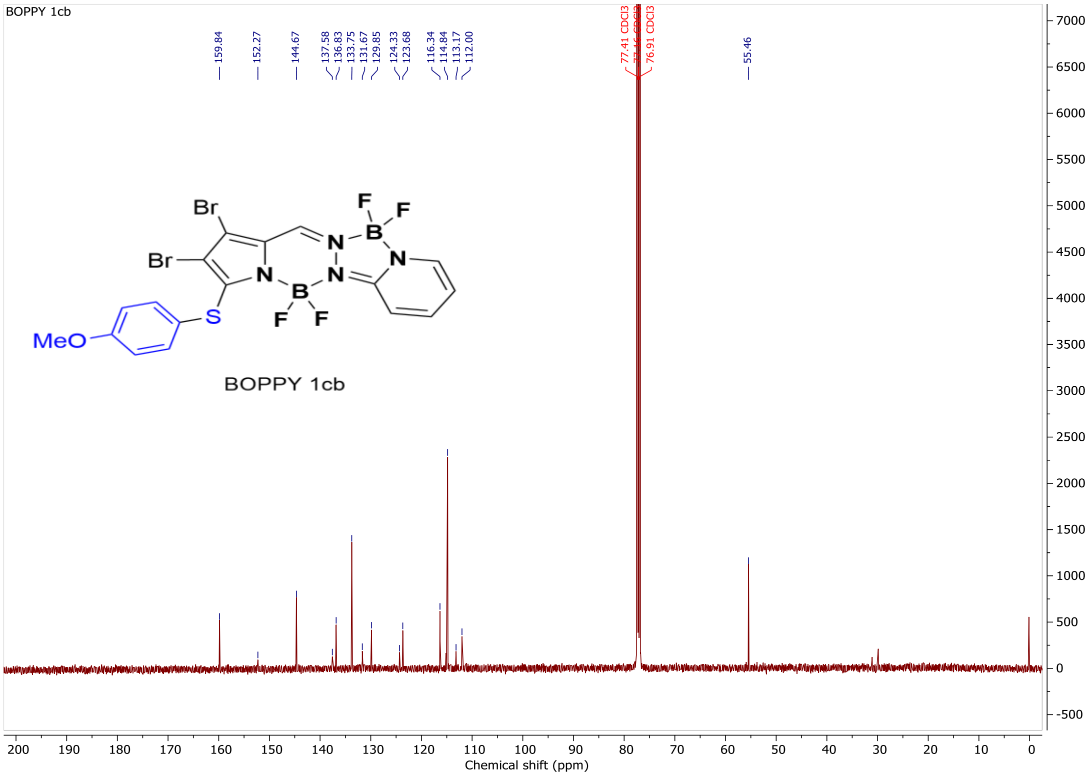

Figure S 62. ^{13}C{1H} NMR (126 MHz, CDCl$_3$) Spectrum

BOPPY 1cc

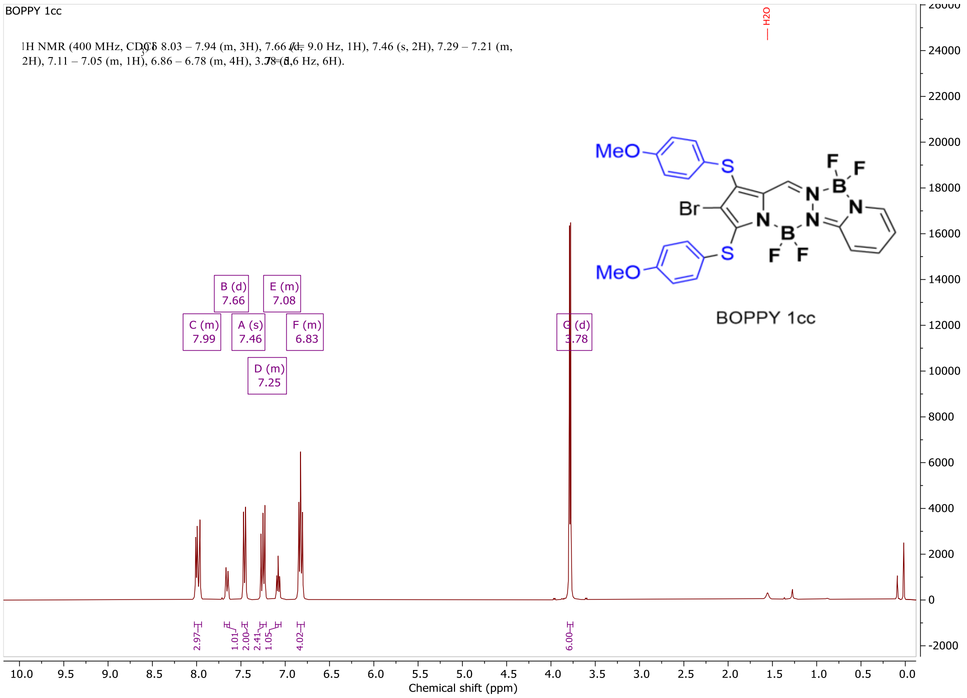

Figure S 63. ^{1}H **NMR (400 MHz,** $CDCl_3$**) Spectrum**

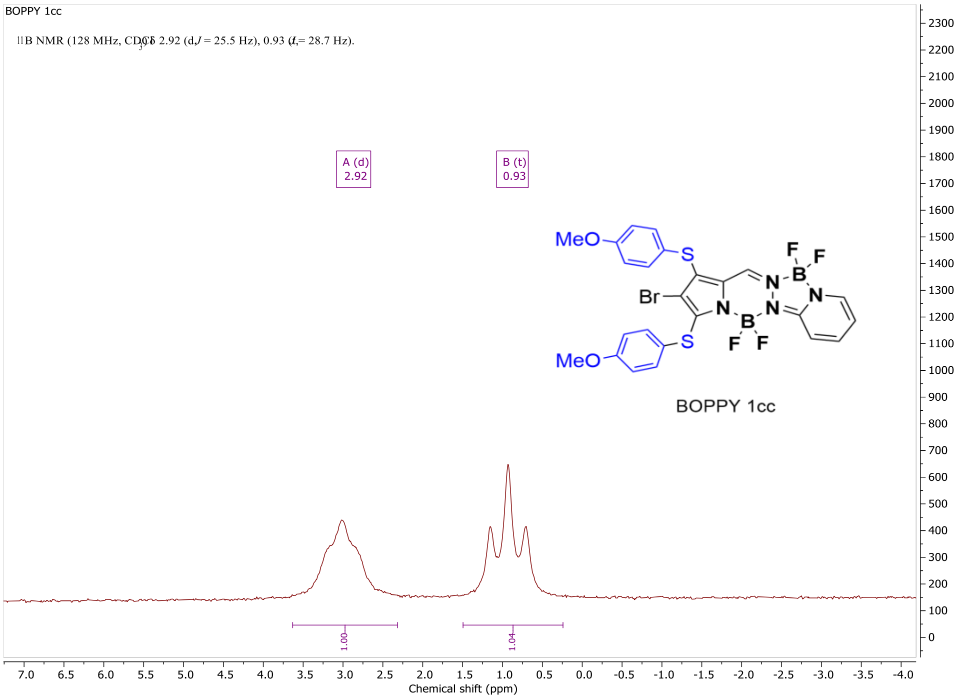

Figure S 64. ^{11}B **NMR (128 MHz,** $CDCl_3$**) Spectrum**

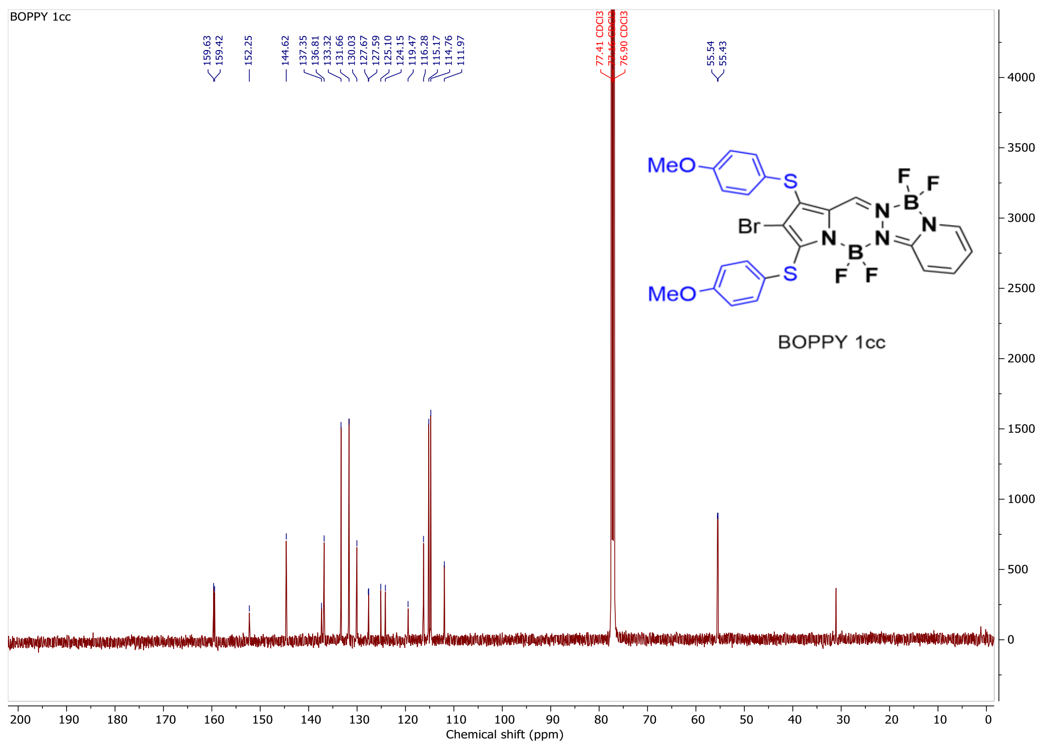

Figure S 65. $^{13}C\{^1H\}$ NMR (126 MHz, $CDCl_3$) Spectrum

4. Computational studies data

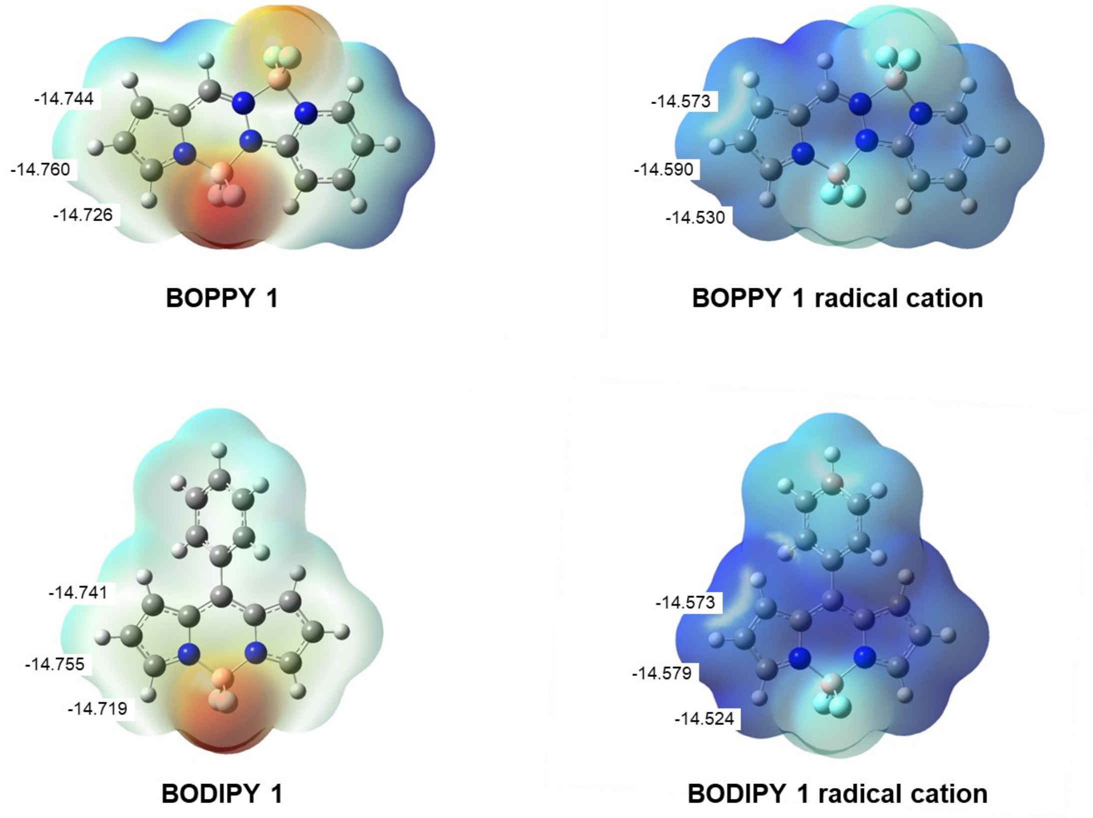

Figure S 66. Molecular Electrostatic Potentials (MESP, in a.u.) at the carbon nuclei for BOPPY 1 and BOPPY 1 radical cation, compared to BODIPY 1 and BODIPY 1 radical cation. Calculated at MN15/6-311++G(d,p) in acetonitrile. In BOPPY 1, the 2-position will be most reactive but in BOPPY 1 radical cation, the halide anion will be most attracted to the α-pyrrolic position.

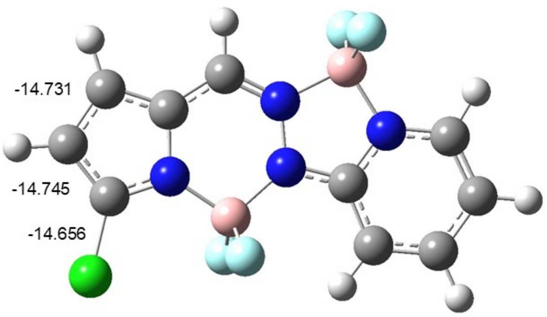

BOPPY 1a

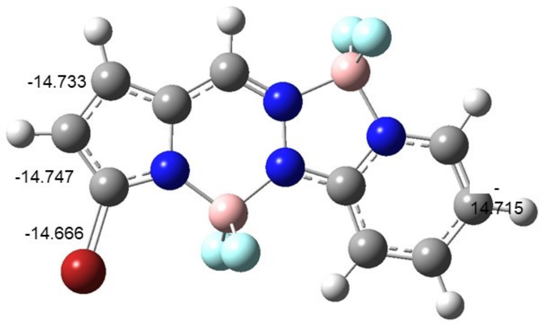

BOPPY 1b

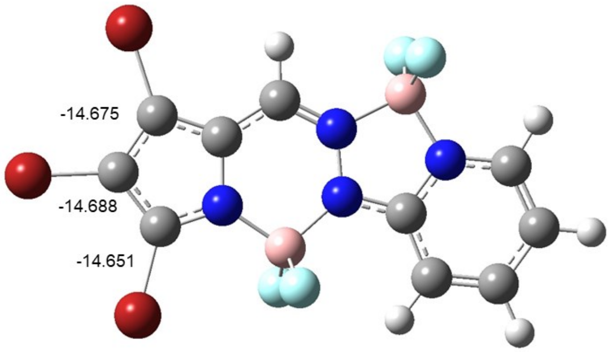

BOPPY 1c

Figure S 67. **Molecular Electrostatic Potentials (MESP, in a.u.) at the carbon nuclei for BOPPY 1a, 1b, and 1c. Calculated at MN15/6-311++G(d,p) in toluene (1a, 1b) and chloroform (1c). In all cases, the α-position will be most reactive toward nucleophilic substitution. In the case of 1c, the second most reactive position will be position 1.**

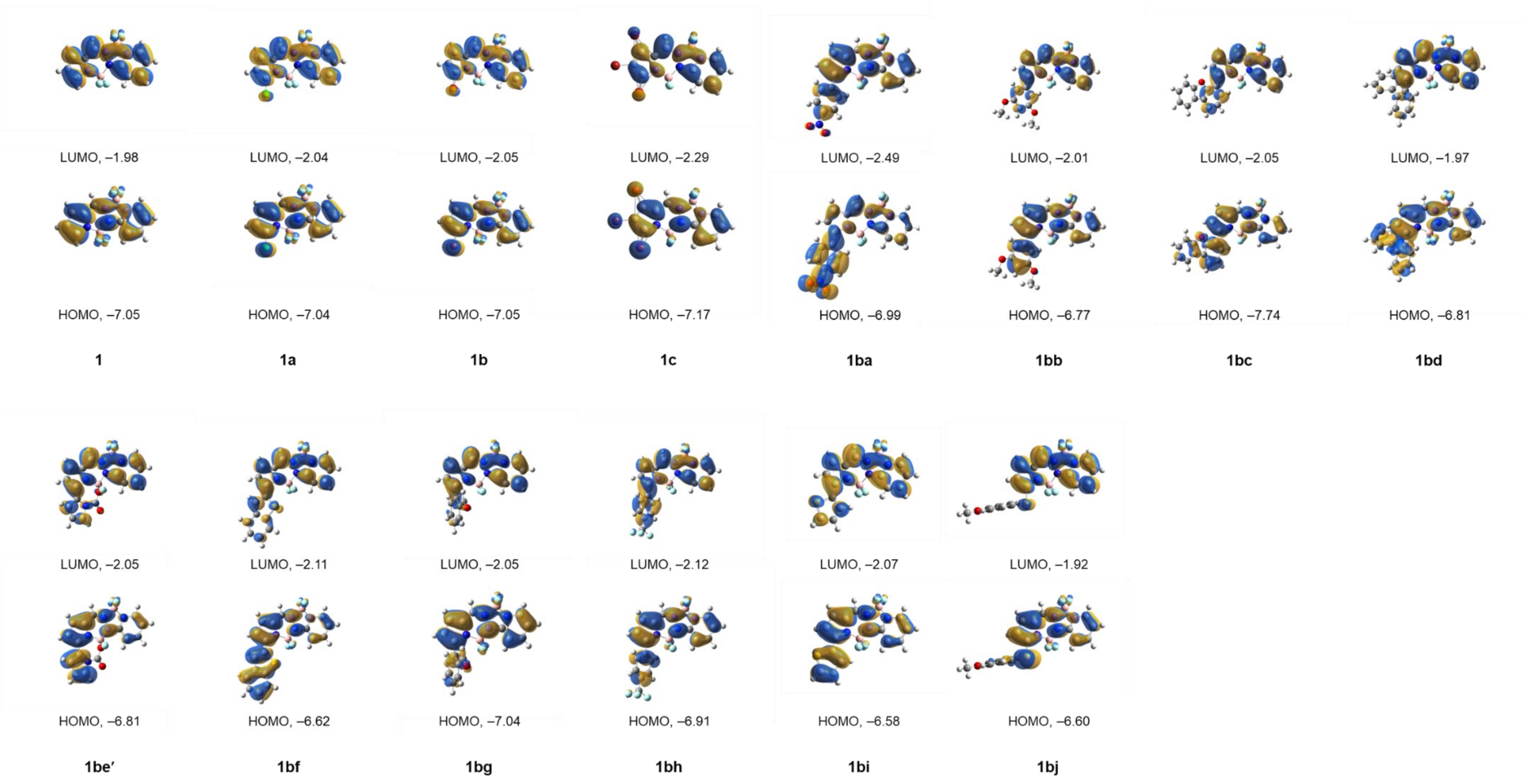

Figure S 68. MN15/6-311++G(d,p) Frontier Orbitals of the series of BOPPYs studied. Energies in eV

Cartesian Coordinates for BOPPY compounds

Ground States

1 -1053.541804 a.u.

B	1.12409	-1.31727	0.05413
B	-1.35305	1.76702	0.01788
C	3.67221	-0.90731	-0.05391
C	4.54099	0.18288	-0.07721
C	3.75374	1.33471	-0.05136
C	2.42366	0.90746	-0.01519
C	1.22774	1.63619	0.00606
H	1.23238	2.71897	0.02076
C	-1.37973	-0.63238	-0.03014
C	-1.92277	-1.92525	-0.05051
H	-1.26917	-2.78484	-0.06399
C	-3.29065	-2.04093	-0.05792
H	-3.73874	-3.02708	-0.07477
C	-4.11907	-0.90184	-0.04551
H	-5.19583	-0.99093	-0.05230
C	-3.53221	0.33066	-0.02393
H	-4.08919	1.25810	-0.01184
F	1.04381	-2.22134	-1.01587
F	1.05289	-2.04021	1.25705
F	-1.54762	2.54689	-1.10763
F	-1.55803	2.49241	1.17755
N	2.39914	-0.47459	-0.01775
N	-0.06564	-0.30896	-0.02320
N	0.05778	1.06911	0.00689
N	-2.18510	0.44933	-0.01554
H	5.61792	0.12853	-0.11099
H	3.89725	-1.96316	-0.05965
H	4.08404	2.36257	-0.06223

1a -1513.073502 a.u.

B	1.12388	-1.31869	0.02325
B	-1.35333	1.76789	0.00780
C	3.67311	-0.90633	-0.02360
C	4.54204	0.18399	-0.03265
C	3.75385	1.33563	-0.02224
C	2.42359	0.90806	-0.00714
C	1.22748	1.63710	0.00236
H	1.23254	2.71995	0.00909
C	-1.37976	-0.63160	-0.01360
C	-1.92191	-1.92496	-0.02161
H	-1.26761	-2.78426	-0.02696
C	-3.28973	-2.04145	-0.02467
H	-3.73745	-3.02789	-0.03136
C	-4.11872	-0.90254	-0.01977
H	-5.19545	-0.99223	-0.02263
C	-3.53254	0.33049	-0.01086
H	-4.09004	1.25769	-0.00584
F	1.04541	-2.17451	-1.08652
F	1.04939	-2.09431	1.19184
F	-1.55061	2.53345	-1.12707
F	-1.55538	2.50798	1.15878
N	2.39947	-0.47408	-0.00841
N	-0.06592	-0.30800	-0.01095
N	0.05771	1.07011	0.00268
N	-2.18544	0.44991	-0.00745
H	5.61938	0.12970	-0.04669
H	4.08335	2.36387	-0.02638
Cl	4.03980	-2.62770	-0.02775

1b -3627.484725 a.u.

B	1.12388	-1.31869	0.02325
B	-1.35333	1.76789	0.00780
C	3.67311	-0.90633	-0.02360
C	4.54204	0.18399	-0.03265
C	3.75385	1.33563	-0.02224
C	2.42359	0.90806	-0.00714
C	1.22748	1.63710	0.00236
H	1.23254	2.71995	0.00909
C	-1.37976	-0.63160	-0.01360
C	-1.92191	-1.92496	-0.02161
H	-1.26761	-2.78426	-0.02696
C	-3.28973	-2.04145	-0.02467
H	-3.73745	-3.02789	-0.03136
C	-4.11872	-0.90254	-0.01977
H	-5.19545	-0.99223	-0.02263
C	-3.53254	0.33049	-0.01086
H	-4.09004	1.25769	-0.00584
F	1.04541	-2.17451	-1.08652
F	1.04939	-2.09431	1.19184
F	-1.55061	2.53345	-1.12707
F	-1.55538	2.50798	1.15878
N	2.39947	-0.47408	-0.00841
N	-0.06592	-0.30800	-0.01095
N	0.05771	1.07011	0.00268
N	-2.18544	0.44991	-0.00745
H	5.61938	0.12970	-0.04669
H	4.08335	2.36387	-0.02638
Br	4.03980	-2.62770	-0.02775

1c -8775.369137 a.u.

B	0.55301	-0.66043	-0.00006
B	-2.78523	1.47590	0.00023
C	2.84734	0.58278	-0.00003
C	3.32153	1.89507	0.00009
C	2.19462	2.71715	-0.00030
C	1.07637	1.87697	-0.00074
C	-0.29139	2.18922	-0.00052
H	-0.62842	3.21864	-0.00039
C	-2.02699	-0.80470	-0.00046
C	-2.11350	-2.20551	-0.00060
H	-1.21091	-2.79987	-0.00100
C	-3.37204	-2.76254	-0.00025
H	-3.47332	-3.84092	-0.00035
C	-4.53046	-1.95633	0.00022
H	-5.51814	-2.39381	0.00048
C	-4.38012	-0.59607	0.00034
H	-5.21021	0.09819	0.00071
F	0.72797	-1.45063	-1.13897
F	0.72743	-1.44936	1.13978
F	-3.21614	2.12058	-1.14045
F	-3.21519	2.12004	1.14157
N	1.50120	0.55517	-0.00046
N	-0.88945	-0.06665	-0.00081
N	-1.22025	1.27339	-0.00044
N	-3.14266	-0.04387	0.00003
Br	2.16778	3.79612	-0.00039
Br	5.16051	2.41106	0.00058
Br	3.43413	-0.31197	0.00011

1ba -1488.729046 a.u.

B	-3.70896	0.81455	0.01299
B	-0.1106	-0.83414	0.07096
C	-5.79885	-0.69807	-0.13301
C	-6.08483	-2.06034	-0.25257
C	-4.86206	-2.73422	-0.28241
C	-3.86343	-1.75781	-0.17806
C	-2.46684	-1.87484	-0.14936
H	-1.98019	-2.83982	-0.22239
C	-1.17412	1.31974	0.16018
C	-1.31265	2.71651	0.20259
H	-2.30005	3.15485	0.22145
C	-0.16200	3.46618	0.18692
H	-0.2269	4.54708	0.20729
C	1.10339	2.84857	0.13550
H	2.01172	3.43523	0.12087
C	1.18828	1.47715	0.13268
F	-3.94085	1.62868	-1.10316
F	-4.06002	1.52747	1.16482
F	0.49222	-1.35519	-1.05273
F	0.28246	-1.49644	1.21900
N	-4.46473	-0.51328	-0.08744
N	-2.19400	0.43563	0.07788
N	-1.67918	-0.83978	-0.03164
N	0.04601	0.72925	0.16266
H	-7.07070	-2.49415	-0.30941
H	-4.69332	-3.79718	-0.36549
H	2.13539	0.98076	0.09425
C	-6.76340	0.50003	-0.05694
C	-7.42462	0.93718	-1.20506
C	-6.97609	1.14810	1.15974
C	-8.29779	2.02258	-1.13655
H	-7.25613	0.42652	-2.16427
C	-7.85025	2.23323	1.22864
H	-6.45517	0.80359	2.06477
C	-8.51098	2.67062	0.08074
H	-8.81852	2.36759	-2.04159
H	-8.01807	2.74375	2.18816
O	-10.0898	4.27519	-0.91665
O	-9.67269	4.47199	1.29220
N	-9.47524	3.86901	0.15606

1bb -1513.237620 a.u.

B	-3.70896	0.81455	0.01299
B	-0.11060	-0.83414	0.07096
C	-5.79885	-0.69807	-0.13301
C	-6.08483	-2.06034	-0.25257
C	-4.86206	-2.73422	-0.28241
C	-3.86343	-1.75781	-0.17806
C	-2.46684	-1.87484	-0.14936
H	-1.98019	-2.83982	-0.22239
C	-1.17412	1.31974	0.16018
C	-1.31265	2.71651	0.20259
H	-2.30005	3.15485	0.22145
C	-0.16200	3.46618	0.18692
H	-0.22690	4.54708	0.20729
C	1.10339	2.84857	0.13550
H	2.01172	3.43523	0.12087
C	1.18828	1.47715	0.13268
F	-3.94085	1.62868	-1.10316
F	-4.06002	1.52747	1.16482
F	0.49222	-1.35519	-1.05273
F	0.28246	-1.49644	1.21900
N	-4.46473	-0.51328	-0.08744
N	-2.19400	0.43563	0.07788
N	-1.67918	-0.83978	-0.03164
N	0.04601	0.72925	0.16266
H	-7.07070	-2.49415	-0.30941
H	-4.69332	-3.79718	-0.36549
H	2.13539	0.98076	0.09425
C	-6.76340	0.50003	-0.05694
C	-7.42462	0.93718	-1.20506
C	-6.97609	1.14810	1.15974
C	-8.29779	2.02258	-1.13655
H	-7.25613	0.42652	-2.16427
C	-7.85025	2.23323	1.22864
H	-6.45517	0.80359	2.06477
C	-8.51098	2.67062	0.08074
H	-9.18095	3.50327	0.13307
O	-8.06846	2.89706	2.47629
O	-8.97494	2.47122	-2.31345
C	-9.46117	3.18436	2.62696
H	-9.67080	3.40552	3.65265
H	-9.71912	4.02726	2.02042
H	-10.03569	2.33528	2.32054
C	-10.38215	2.52222	-2.06434
H	-10.61820	3.42133	-1.53445
H	-10.90987	2.50773	-2.99504
H	-10.67132	1.67578	-1.47714

1bc -1589.170586 a.u.

B	0.94124	-1.01494	0.02490
B	-1.53596	2.07164	0.00944
C	3.49047	-0.60258	-0.02196
C	4.35941	0.48774	-0.03101
C	3.57121	1.63938	-0.02060
C	2.24095	1.21181	-0.00549
C	1.04485	1.94085	0.00400
H	1.04990	3.02370	0.01073
C	-1.56239	-0.32785	-0.01196
C	-2.10455	-1.62121	-0.01997
H	-1.45025	-2.48050	-0.02531
C	-3.47236	-1.73770	-0.02303
H	-3.92009	-2.72414	-0.02971
C	-4.30136	-0.59879	-0.01812
H	-5.37809	-0.68848	-0.02099
C	-3.71518	0.63424	-0.00921
F	0.86277	-1.87076	-1.08487
F	0.86675	-1.79056	1.19348
F	-1.73324	2.83720	-1.12543
F	-1.73802	2.81173	1.16043
N	2.21683	-0.17033	-0.00677
N	-0.24856	-0.00425	-0.00930
N	-0.12493	1.37386	0.00432
N	-2.36807	0.75366	-0.00581
H	5.43675	0.43345	-0.04505
H	3.90072	2.66762	-0.02474
H	-4.26654	1.55123	-0.00425
C	3.81132	-2.10878	-0.02559
C	2.77358	-3.05286	-0.01473
C	5.11747	-2.55942	-0.03966
C	3.04860	-4.41711	-0.01798
H	1.72996	-2.70528	-0.00347
C	5.40074	-3.96006	-0.04295
C	4.36999	-4.88488	-0.03214
H	2.22101	-5.14176	-0.00941
C	6.85129	-4.13491	-0.05861
C	7.45924	-2.84169	-0.06492
H	4.57977	-5.96369	-0.03449
C	7.63276	-5.27821	-0.06733
C	8.83504	-2.71431	-0.07965
C	9.02745	-5.13778	-0.08238
H	7.17272	-6.27630	-0.06263
C	9.61873	-3.87796	-0.08817
H	9.31279	-1.72475	-0.08460
H	9.65915	-6.03834	-0.08953
H	10.71505	-3.78832	-0.09968
O	6.40003	-1.77395	-0.05332

1bd -1515.184726 a.u.

B	0.55301	-0.66043	-0.00006
B	-2.78523	1.47590	0.00023
C	2.84734	0.58278	-0.00003
C	3.32153	1.89507	0.00009
C	2.19462	2.71715	-0.00030
C	1.07637	1.87697	-0.00074
C	-0.29139	2.18922	-0.00052
H	-0.62842	3.21864	-0.00039
C	-2.02699	-0.80470	-0.00046
C	-2.11350	-2.20551	-0.00060
H	-1.21091	-2.79987	-0.00100
C	-3.37204	-2.76254	-0.00025
H	-3.47332	-3.84092	-0.00035
C	-4.53046	-1.95633	0.00022
H	-5.51814	-2.39381	0.00048
C	-4.38012	-0.59607	0.00034
H	-5.21021	0.09819	0.00071
F	0.72797	-1.45063	-1.13897
F	0.72743	-1.44936	1.13978
F	-3.21614	2.12058	-1.14045
F	-3.21519	2.12004	1.14157
N	1.50120	0.55517	-0.00046
N	-0.88945	-0.06665	-0.00081
N	-1.22025	1.27339	-0.00044
N	-3.14266	-0.04387	0.00003
H	4.35943	2.18629	0.00037
H	2.16778	3.79612	-0.00039
C	3.69188	-0.70499	0.00017
C	3.06402	-1.95089	0.00001
C	5.08446	-0.62584	0.00041
C	3.82865	-3.11732	0.00077
H	1.96612	-2.01295	0.00060
C	5.84947	-1.79253	0.00017
C	5.22183	-3.03817	0.00049
H	3.33383	-4.09939	0.00127
H	6.94744	-1.72982	-0.00006
H	5.82450	-3.95799	0.00102
C	5.77773	0.74929	0.00034
C	6.34482	1.24448	-1.17426
C	5.83863	1.49978	1.17447
C	6.97323	2.48960	-1.17446
H	6.29731	0.65222	-2.09958
C	6.46634	2.74573	1.17421
H	5.39157	1.10972	2.10028
C	7.03373	3.24066	0.00005
H	7.42079	2.87970	-2.10010
H	6.51385	3.33742	2.10001
H	7.52922	4.22239	-0.00037

1be’ -1607.861644 a.u.

B	0.55301	-0.66043	-0.00006
B	-2.78523	1.47590	0.00023
C	2.84734	0.58278	-0.00003
C	3.32153	1.89507	0.00009
C	2.19462	2.71715	-0.00030
C	1.07637	1.87697	-0.00074
C	-0.29139	2.18922	-0.00052
H	-0.62842	3.21864	-0.00039
C	-2.02699	-0.80470	-0.00046
C	-2.11350	-2.20551	-0.00060
H	-1.21091	-2.79987	-0.00100
C	-3.37204	-2.76254	-0.00025
H	-3.47332	-3.84092	-0.00035
C	-4.53046	-1.95633	0.00022
H	-5.51814	-2.39381	0.00048
C	-4.38012	-0.59607	0.00034
H	-5.21021	0.09819	0.00071
F	0.72797	-1.45063	-1.13897
F	0.72743	-1.44936	1.13978
F	-3.21614	2.12058	-1.14045
F	-3.21519	2.12004	1.14157
N	1.50120	0.55517	-0.00046
N	-0.88945	-0.06665	-0.00081
N	-1.22025	1.27339	-0.00044
N	-3.14266	-0.04387	0.00003
H	4.35943	2.18629	0.00037
H	2.16778	3.79612	-0.00039
C	3.69188	-0.70499	0.00017
C	3.29067	-2.04820	0.00007
N	5.08301	-0.66168	0.00054
C	4.48280	-2.84675	0.00055
H	2.27007	-2.41587	-0.00022
C	5.57222	-1.96467	0.00083
H	4.53117	-3.93049	0.00065
H	6.64181	-2.17075	0.00111
C	5.90129	0.55952	0.00102
O	6.26441	1.09716	-1.12701
O	6.26004	1.09921	1.12948
C	7.67697	1.29198	1.13727
C	8.02562	2.56909	0.35048
H	8.86173	3.05381	0.80967
H	8.27293	2.31042	-0.65790
H	7.18443	3.23037	0.35300
C	8.36349	0.08043	0.47967
H	9.42079	0.14391	0.63126
H	7.99353	-0.82172	0.92030
H	8.15314	0.07731	-0.56944
C	8.16772	1.43402	2.59006
H	9.22593	1.59233	2.59519
H	7.68018	2.26734	3.05135
H	7.93803	0.54110	3.13303

1bf -1758.617261 a.u.

B	0.21257	-0.50317	-0.14150
B	-3.30192	1.30421	0.03486
C	2.41673	0.92987	-0.08624
C	2.72131	2.30583	-0.07483
C	1.52368	3.00452	-0.09677
C	0.50196	2.04697	-0.11698
C	-0.88297	2.23833	-0.08659
H	-1.30598	3.23512	-0.09808
C	-2.33372	-0.89622	0.06305
C	-2.29291	-2.29991	0.08441
H	-1.34074	-2.80977	0.06051
C	-3.49385	-2.96903	0.13778
H	-3.49552	-4.05209	0.15512
C	-4.72145	-2.27244	0.16967
H	-5.66412	-2.79824	0.21259
C	-4.69639	-0.90475	0.14192
H	-5.58589	-0.28854	0.15902
F	0.34283	-1.19186	-1.35991
F	0.53510	-1.37032	0.90601
F	-3.84368	1.87319	-1.10093
F	-3.74954	1.93875	1.17714
N	1.06574	0.77690	-0.11209
N	-1.27307	-0.05510	0.01162
N	-1.73222	1.24654	-0.02760
N	-3.51579	-0.24154	0.08788
H	3.71824	2.72028	-0.06865
H	1.37887	4.07420	-0.09617
C	3.44951	-0.21229	-0.06518
C	4.84653	-0.04264	-0.03148
C	4.39284	-2.33242	-0.04919
C	5.46466	-1.36253	-0.02073
H	5.39271	0.89767	-0.01582
C	4.67344	-3.68387	-0.04670
C	6.78106	-1.77665	0.00951
C	6.01856	-4.08821	-0.01574
H	3.87009	-4.43335	-0.06830
C	7.04899	-3.15577	0.01173
H	7.60672	-1.05180	0.03151
H	6.25445	-5.16287	-0.01350
H	8.09442	-3.49792	0.03569
S	3.14151	-1.58529	-0.07666

1bg -1397.604386 a.u.

B	-3.70896	0.81455	0.01299
B	-0.11060	-0.83414	0.07096
C	-5.79885	-0.69807	-0.13301
C	-6.08483	-2.06034	-0.25257
C	-4.86206	-2.73422	-0.28241
C	-3.86343	-1.75781	-0.17806
C	-2.46684	-1.87484	-0.14936
H	-1.98019	-2.83982	-0.22239
C	-1.17412	1.31974	0.16018
C	-1.31265	2.71651	0.20259
H	-2.30005	3.15485	0.22145
C	-0.16200	3.46618	0.18692
H	-0.22690	4.54708	0.20729
C	1.10339	2.84857	0.13550
H	2.01172	3.43523	0.12087
C	1.18828	1.47715	0.13268
F	-3.94085	1.62868	-1.10316
F	-4.06002	1.52747	1.16482
F	0.49222	-1.35519	-1.05273
F	0.28246	-1.49644	1.21900
N	-4.46473	-0.51328	-0.08744
N	-2.19400	0.43563	0.07788
N	-1.67918	-0.83978	-0.03164
N	0.04601	0.72925	0.16266
H	-7.07070	-2.49415	-0.30941
H	-4.69332	-3.79718	-0.36549
H	2.13539	0.98076	0.09425
C	-6.76340	0.50003	-0.05694
C	-7.42462	0.93718	-1.20506
C	-6.97609	1.14810	1.15974
C	-8.29779	2.02258	-1.13655
C	-7.85025	2.23323	1.22864
H	-6.45517	0.80359	2.06477
C	-8.51098	2.67062	0.08074
H	-8.81852	2.36759	-2.04159
H	-8.01807	2.74375	2.18816
H	-9.18095	3.50327	0.13307
C	-7.18866	0.22203	-2.54837
O	-7.80636	0.63130	-3.61811
H	-6.57780	-0.53482	-2.59437

1bh -1621.246568 a.u.

B	-3.70896	0.81455	0.01299
B	-0.11060	-0.83414	0.07096
C	-5.79885	-0.69807	-0.13301
C	-6.08483	-2.06034	-0.25257
C	-4.86206	-2.73422	-0.28241
C	-3.86343	-1.75781	-0.17806
C	-2.46684	-1.87484	-0.14936
H	-1.98019	-2.83982	-0.22239
C	-1.17412	1.31974	0.16018
C	-1.31265	2.71651	0.20259
H	-2.30005	3.15485	0.22145
C	-0.16200	3.46618	0.18692
H	-0.22690	4.54708	0.20729
C	1.10339	2.84857	0.13550
H	2.01172	3.43523	0.12087
C	1.18828	1.47715	0.13268
F	-3.94085	1.62868	-1.10316
F	-4.06002	1.52747	1.16482
F	0.49222	-1.35519	-1.05273
F	0.28246	-1.49644	1.21900
N	-4.46473	-0.51328	-0.08744
N	-2.19400	0.43563	0.07788
N	-1.67918	-0.83978	-0.03164
N	0.04601	0.72925	0.16266
H	-7.07070	-2.49415	-0.30941
H	-4.69332	-3.79718	-0.36549
H	2.13539	0.98076	0.09425
C	-6.76340	0.50003	-0.05694
C	-7.42462	0.93718	-1.20506
C	-6.97609	1.14810	1.15974
C	-8.29779	2.02258	-1.13655
H	-7.25613	0.42652	-2.16427
C	-7.85025	2.23323	1.22864
H	-6.45517	0.80359	2.06477
C	-8.51098	2.67062	0.08074
H	-8.81852	2.36759	-2.04159
H	-8.01807	2.74375	2.18816
C	-9.47524	3.86901	0.15606
F	-10.74941	3.42381	0.12807
F	-9.26079	4.68603	-0.89703
F	-9.26081	4.54773	1.30317

1bi -1605.126137 a.u.

B	1.12409	-1.31727	0.05413
B	-1.35305	1.76702	0.01788
C	3.67221	-0.90731	-0.05391
C	4.54099	0.18288	-0.07721
C	3.75374	1.33471	-0.05136
C	2.42366	0.90746	-0.01519
C	1.22774	1.63619	0.00606
H	1.23238	2.71897	0.02076
C	-1.37973	-0.63238	-0.03014
C	-1.92277	-1.92525	-0.05051
H	-1.26917	-2.78484	-0.06399
C	-3.29065	-2.04093	-0.05792
H	-3.73874	-3.02708	-0.07477
C	-4.11907	-0.90184	-0.04551
H	-5.19583	-0.99093	-0.05230
C	-3.53221	0.33066	-0.02393
H	-4.08919	1.25810	-0.01184
F	1.04381	-2.22134	-1.01587
F	1.05289	-2.04021	1.25705
F	-1.54762	2.54689	-1.10763
F	-1.55803	2.49241	1.17755
N	2.39914	-0.47459	-0.01775
N	-0.06564	-0.30896	-0.02320
N	0.05778	1.06911	0.00689
N	-2.18510	0.44933	-0.01554
H	5.61792	0.12853	-0.11099
H	4.08404	2.36257	-0.06223
C	3.99322	-2.41346	-0.06210
C	3.06690	-3.43232	-0.03728
S	5.55662	-3.00524	-0.10358
C	3.69878	-4.71739	-0.05455
H	1.98378	-3.30069	-0.00829
C	5.07093	-4.60532	-0.09074
H	3.14189	-5.65601	-0.04026
H	5.77530	-5.43460	-0.10924

1bj -1796.904334 a.u.

B	1.12409	-1.31727	0.05413
B	-1.35305	1.76702	0.01788
C	3.67221	-0.90731	-0.05391
C	4.54099	0.18288	-0.07721
C	3.75374	1.33471	-0.05136
C	2.42366	0.90746	-0.01519
C	1.22774	1.63619	0.00606
H	1.23238	2.71897	0.02076
C	-1.37973	-0.63238	-0.03014
C	-1.92277	-1.92525	-0.05051
H	-1.26917	-2.78484	-0.06399
C	-3.29065	-2.04093	-0.05792
H	-3.73874	-3.02708	-0.07477
C	-4.11907	-0.90184	-0.04551
H	-5.19583	-0.99093	-0.05230
C	-3.53221	0.33066	-0.02393
H	-4.08919	1.25810	-0.01184
F	1.04381	-2.22134	-1.01587
F	1.05289	-2.04021	1.25705
F	-1.54762	2.54689	-1.10763
F	-1.55803	2.49241	1.17755
N	2.39914	-0.47459	-0.01775
N	-0.06564	-0.30896	-0.02320
N	0.05778	1.06911	0.00689
N	-2.18510	0.44933	-0.01554
H	5.61792	0.12853	-0.11099
H	4.08404	2.36257	-0.06223
S	4.04325	-2.64818	-0.06338
C	5.80760	-2.87943	-0.10790
C	6.46863	-2.96918	-1.33324
C	6.52934	-2.97081	1.08218
C	7.85100	-3.15093	-1.36840
H	5.89923	-2.89787	-2.27129
C	7.91227	-3.15163	1.04718
H	6.00854	-2.89991	2.04803
C	8.57315	-3.24184	-0.17783
H	8.37201	-3.22231	-2.33418
H	8.48113	-3.22323	1.98567
O	9.99049	-3.42812	-0.21420
C	10.28819	-4.82679	-0.21750
H	11.34872	-4.96565	-0.24751
H	9.89445	-5.27734	0.66955
H	9.84416	-5.28392	-1.07701

1ca -6753.010044 a.u.

B	-1.03047	-1.35593	-0.00017
B	-3.36181	1.85116	0.00007
C	1.55792	-1.04514	-0.00005
C	2.47651	0.00793	0.00001
C	1.72794	1.18711	0.00008
C	0.37980	0.81779	0.00008
C	-0.78101	1.60975	0.00009
H	-0.71980	2.69149	0.00003
C	-3.48568	-0.55002	0.00022
C	-4.07323	-1.82222	0.00025
H	-3.44900	-2.70438	0.00030
C	-5.44934	-1.88372	0.00022
H	-5.93566	-2.85149	0.00024
C	-6.23425	-0.71234	0.00017
H	-7.31340	-0.76028	0.00015
C	-5.59949	0.50088	0.00013
H	-6.11978	1.44967	0.00008
F	-1.14644	-2.15047	-1.13959
F	-1.14634	-2.15120	1.13874
F	-3.52167	2.60364	-1.14163
F	-3.52169	2.60381	1.14164
N	0.30120	-0.56483	0.00001
N	-2.15364	-0.27594	0.00026
N	-1.97328	1.08867	0.00013
N	-4.24636	0.56430	0.00015
Br	4.32707	-0.14625	-0.00001
Br	1.93963	-2.85645	-0.00029
C	2.27534	2.62654	0.00017
C	3.52447	3.20359	0.09982
C	3.32642	4.63622	0.02803
H	4.48115	2.70222	0.21091
C	1.96890	4.83971	-0.11088
H	4.10979	5.38645	0.07625
H	1.32257	5.70655	-0.20531
S	1.29957	3.61561	-0.13079

1cb -6944.787579 a.u.

B	-1.03047	-1.35593	-0.00017
B	-3.36181	1.85116	0.00007
C	1.55792	-1.04514	-0.00005
C	2.47651	0.00793	0.00001
C	1.72794	1.18711	0.00008
C	0.37980	0.81779	0.00008
C	-0.78101	1.60975	0.00009
H	-0.71980	2.69149	0.00003
C	-3.48568	-0.55002	0.00022
C	-4.07323	-1.82222	0.00025
H	-3.44900	-2.70438	0.00030
C	-5.44934	-1.88372	0.00022
H	-5.93566	-2.85149	0.00024
C	-6.23425	-0.71234	0.00017
H	-7.31340	-0.76028	0.00015
C	-5.59949	0.50088	0.00013
H	-6.11978	1.44967	0.00008
F	-1.14644	-2.15047	-1.13959
F	-1.14634	-2.15120	1.13874
F	-3.52167	2.60364	-1.14163
F	-3.52169	2.60381	1.14164
N	0.30120	-0.56483	0.00001
N	-2.15364	-0.27594	0.00026
N	-1.97328	1.08867	0.00013
N	-4.24636	0.56430	0.00015
Br	2.38798	2.92274	0.00019
Br	4.32707	-0.14625	-0.00001
S	1.92497	-2.78689	-0.00028
C	0.76323	-3.83759	-0.00032
C	0.23535	-4.29376	-1.20851
C	0.25655	-4.31726	1.20747
C	-0.79943	-5.22887	-1.20881
H	0.63451	-3.91482	-2.16052
C	-0.77788	-5.25340	1.20732
H	0.67261	-3.95797	2.15980
C	-1.30601	-5.70914	-0.00053
H	-1.21599	-5.58800	-2.16107
H	-1.17690	-5.63172	2.15976
O	-2.36693	-6.66797	-0.00117
C	-1.81724	-7.98810	-0.00422
H	-2.61141	-8.70511	-0.01310
H	-1.20873	-8.11906	-0.87455
H	-1.22027	-8.12790	0.87269

1cc -5114.208637 a.u.

B	-1.03047	-1.35593	-0.00017
B	-3.36181	1.85116	0.00007
C	1.55792	-1.04514	-0.00005
C	2.47651	0.00793	0.00001
C	1.72794	1.18711	0.00008
C	0.37980	0.81779	0.00008
C	-0.78101	1.60975	0.00009
H	-0.71980	2.69149	0.00003
C	-3.48568	-0.55002	0.00022
C	-4.07323	-1.82222	0.00025
H	-3.44900	-2.70438	0.00030
C	-5.44934	-1.88372	0.00022
H	-5.93566	-2.85149	0.00024
C	-6.23425	-0.71234	0.00017
H	-7.31340	-0.76028	0.00015
C	-5.59949	0.50088	0.00013
H	-6.11978	1.44967	0.00008
F	-1.14644	-2.15047	-1.13959
F	-1.14634	-2.15120	1.13874
F	-3.52167	2.60364	-1.14163
F	-3.52169	2.60381	1.14164
N	0.30120	-0.56483	0.00001
N	-2.15364	-0.27594	0.00026
N	-1.97328	1.08867	0.00013
N	-4.24636	0.56430	0.00015
Br	4.32707	-0.14625	-0.00001
S	1.92497	-2.78689	-0.00028
C	0.76323	-3.83759	-0.00032
C	0.23535	-4.29376	-1.20851
C	0.25655	-4.31726	1.20747
C	-0.79943	-5.22887	-1.20881
H	0.63451	-3.91482	-2.16052
C	-0.77788	-5.25340	1.20732
H	0.67261	-3.95797	2.15980
C	-1.30601	-5.70914	-0.00053
H	-1.21599	-5.58800	-2.16107
H	-1.17690	-5.63172	2.15976
O	-2.36693	-6.66797	-0.00117
C	-1.81724	-7.98810	-0.00422
H	-2.61141	-8.70511	-0.01310
H	-1.20873	-8.11906	-0.87455
H	-1.22027	-8.12790	0.87269
S	2.36065	2.85086	0.00019
C	2.53797	3.41902	1.67773
C	1.52455	4.17358	2.26942
C	3.68997	3.10980	2.40080
C	1.66348	4.61936	3.58365
H	0.61679	4.41784	1.69884
C	3.82867	3.55480	3.71577
H	4.48872	2.51495	1.93467
C	2.81574	4.30959	4.30722
H	0.86501	5.21465	4.04988
H	4.73691	3.31050	4.28577
O	2.95787	4.76672	5.65471
C	3.66575	6.00918	5.66512

H	3.69375	6.39537	6.66260
H	4.66471	5.85347	5.31477
H	3.16848	6.70836	5.02577

Excited States

1, S_1 -1053.534856 a.u.

B	1.11790900	-1.32106400	-0.00009600
B	-1.34790300	1.77403000	-0.00003400
C	3.66535200	-0.94992800	0.00008300
C	4.56272600	0.13079200	0.00014700
C	3.80992000	1.29668700	0.00009000
C	2.43980400	0.90257000	0.00000700
C	1.27027200	1.65000700	-0.00002000
H	1.26056200	2.72958800	-0.00003100
C	-1.39934900	-0.63938000	0.00008800
C	-1.92162300	-1.92534800	0.00006100
H	-1.25351700	-2.77582900	0.00004500
C	-3.30572600	-2.06091400	0.00007900
H	-3.75283400	-3.04583500	0.00007400
C	-4.12110800	-0.90821100	0.00010300
H	-5.19935800	-0.99018300	0.00011900
C	-3.54289400	0.34195600	0.00010100
H	-4.12011500	1.25766000	0.00011200
F	1.00236700	-2.11206700	-1.13982300
F	1.00241800	-2.11236800	1.13942400
F	-1.52137800	2.54760900	-1.13807500
F	-1.52121000	2.54780100	1.13789600
N	2.39120100	-0.48486400	0.00000000
N	-0.08060100	-0.28110600	0.00005200
N	0.04652600	1.04920400	-0.00005300
N	-2.19399400	0.47950800	0.00009600
H	5.63834200	0.04672000	0.00022000
H	3.86863100	-2.01098200	0.00009300
H	4.16232700	2.31668300	0.00011100

1a, S_1 -1513.066827 a.u.

B	0.90906600	-0.89326300	-0.00007900
B	-2.14862100	1.61939400	0.00003800
C	3.33312100	0.04178800	0.00003600
C	3.98162900	1.28508400	0.00003100
C	2.98733700	2.25036900	-0.00006300
C	1.73660700	1.56612800	-0.00007100
C	0.43584200	2.04953800	-0.00010000
H	0.20286300	3.10351800	-0.00014100
C	-1.68887600	-0.74998400	-0.00004000
C	-1.93050400	-2.11874000	-0.00009200
H	-1.09977800	-2.81112600	-0.00017300
C	-3.25386000	-2.54010200	-0.00002600
H	-3.48376100	-3.59718400	-0.00005900
C	-4.29486400	-1.58574100	0.00008200
H	-5.33137300	-1.89354600	0.00013600
C	-3.99143900	-0.24274700	0.00011300
H	-4.74720900	0.53224500	0.00018900
F	0.96082700	-1.68483000	-1.14155900
F	0.96078900	-1.68484000	1.14139500
F	-2.48328100	2.33783200	-1.13793600
F	-2.48308900	2.33779600	1.13808900
N	1.98437100	0.19801800	-0.00004800
N	-0.47625900	-0.12275000	-0.00009100
N	-0.63345300	1.20553700	-0.00008600
N	-2.70202700	0.17416300	0.00005000
H	5.05080700	1.42550300	0.00009600
H	3.10801900	3.32254500	-0.00008500
Cl	4.04721200	-1.49320900	0.00013800

1b, S_1 -3627.478107 a.u.

B	0.55895000	-0.65126900	0.00004300
B	-2.77787600	1.47677800	0.00005600
C	2.85975700	0.56437400	-0.00004600
C	3.34864300	1.88060400	-0.00006100
C	2.24536100	2.71748300	-0.00011700
C	1.08740200	1.88554700	-0.00010300
C	-0.26153500	2.21258900	-0.00008400
H	-0.61569500	3.23230200	-0.00009000
C	-2.03587800	-0.81962800	-0.00010600
C	-2.11125900	-2.20838900	-0.00013000
H	-1.20327000	-2.79584200	-0.00018100
C	-3.37369800	-2.78527000	-0.00008800
H	-3.47468600	-3.86238700	-0.00010500
C	-4.52262000	-1.96359400	-0.00002500
H	-5.51435400	-2.39426100	0.00000200
C	-4.38283800	-0.59421600	0.00000500
H	-5.22641300	0.08405300	0.00006000
F	0.70141100	-1.43254500	-1.14146400
F	0.70131100	-1.43215900	1.14182800
F	-3.19819400	2.14890800	-1.13794500
F	-3.19805900	2.14873100	1.13821500
N	1.49924500	0.55780900	-0.00011100
N	-0.90835600	-0.05153800	-0.00012600
N	-1.22414000	1.24871900	-0.00005000
N	-3.15316700	-0.02526800	-0.00002900
H	4.39229900	2.15251800	-0.00004200
H	2.23377200	3.79636800	-0.00014700
Br	3.85227700	-0.99090800	0.00003000

1c, S_1 -8775.362515 a.u.

B	-1.02346400	-1.34184700	-0.00005800
B	-3.36580700	1.85154400	0.00010800
C	1.56369200	-1.04855900	-0.00007500
C	2.49075100	0.00269800	-0.00004200
C	1.76110200	1.18591500	0.00002300
C	0.37915300	0.83212400	0.00004000
C	-0.75283500	1.63007300	0.00008700
H	-0.70599100	2.70885400	0.00011400
C	-3.49787300	-0.55904700	0.00011200
C	-4.06899600	-1.83196700	0.00010500
H	-3.43425300	-2.70737500	0.00008200
C	-5.45120200	-1.91291900	0.00012800
H	-5.93284700	-2.88189800	0.00012300
C	-6.23055100	-0.73599200	0.00015600
H	-7.31053600	-0.78285800	0.00017600
C	-5.60640200	0.49175900	0.00015700
H	-6.14836000	1.42882100	0.00017400
F	-1.16834000	-2.11856100	-1.14155900
F	-1.16828500	-2.11880700	1.14128100
F	-3.52420200	2.62472500	-1.13825300
F	-3.52415000	2.62474100	1.13846300
N	0.29399700	-0.55199900	-0.00000400
N	-2.17357900	-0.25042500	0.00008500
N	-2.00073200	1.08048800	0.00008300
N	-4.25763300	0.57838700	0.00013400
Br	2.40660800	2.91680400	0.00009200
Br	4.33577100	-0.17333300	-0.00011700
Br	1.92584200	-2.85040200	-0.00016200

1ba, S_1 -1488.719779 a.u.

B	-0.45419700	-0.19895700	0.18437900
B	-4.27424300	0.81648600	-0.03849200
C	1.42134400	1.67248500	0.05717200
C	1.39210400	3.11137600	-0.00757100
C	0.09679400	3.52285200	0.06518300
C	-0.70688600	2.34359400	0.13925800
C	-2.09081500	2.25579800	0.11015700
H	-2.71750400	3.13588600	0.10350700
C	-2.86435400	-1.13787700	-0.08109800
C	-2.51367800	-2.48910700	-0.11769900
H	-1.47372800	-2.78004800	-0.07754900
C	-3.54168900	-3.40867100	-0.20878700
H	-3.30928600	-4.46554500	-0.24013100
C	-4.88444500	-2.98594900	-0.26058300
H	-5.69312600	-3.69895900	-0.33297900
C	-5.16242400	-1.64019400	-0.21356600
F	0.04024700	-0.97627000	-0.85511200
F	-0.25216800	-0.83940800	1.40999000
F	-4.81605900	1.37722500	-1.17558600
F	-4.92665200	1.24210300	1.09895800
N	0.11443400	1.23017000	0.15716000
N	-2.01852500	-0.07173500	0.00646100
N	-2.71879000	1.07486100	0.04946500
N	-4.16121300	-0.73818500	-0.12315200
H	2.26326700	3.74393000	-0.06093000
H	-0.28671700	4.53109600	0.05975300
H	-6.16726900	-1.23933500	-0.24287600
C	2.60956000	0.88539000	0.03856200
C	3.81692500	1.49051400	-0.42619800
C	2.67763000	-0.46731800	0.47798900
C	4.99908200	0.79374500	-0.47107600
H	3.80318900	2.50959500	-0.78925400
C	3.86114700	-1.16574900	0.44508700
H	1.80290700	-0.95275500	0.88168400
C	5.02413500	-0.54165600	-0.03513700
H	5.90595500	1.25072900	-0.84208000
H	3.91429700	-2.18775100	0.79439500
O	7.26706600	-0.69614100	-0.48748100
O	6.24974600	-2.45084200	0.30450100
N	6.24849600	-1.27037600	-0.07472700

1bb, S_1 -1513.230369 a.u.

B	-0.62999500	-0.18596800	0.16461000
B	-4.39970800	0.97912200	0.00426500
C	1.31643200	1.60567400	0.22823900
C	1.35522900	3.02720200	0.30983100
C	0.07101000	3.49688000	0.41547600
C	-0.78708200	2.35806000	0.36586700
C	-2.17479400	2.32589000	0.32207300
H	-2.77524900	3.21976300	0.39775600
C	-3.06411700	-1.01330800	-0.23187100
C	-2.76744400	-2.36459100	-0.40044600
H	-1.73854800	-2.69532600	-0.37864800
C	-3.82605200	-3.23569800	-0.59493300
H	-3.63402200	-4.29204600	-0.73088500
C	-5.15326200	-2.74969200	-0.61648600
H	-5.98933500	-3.41767700	-0.76969100
C	-5.38299600	-1.40759900	-0.43584400
F	-0.14674000	-0.89231900	-0.93195300
F	-0.48386500	-0.94774900	1.33180200
F	-4.91717700	1.69086900	-1.06898700
F	-5.06442900	1.32312900	1.17252600
N	-0.01027300	1.21107300	0.27820400
N	-2.18811000	0.01055500	-0.02741700
N	-2.84897800	1.16572800	0.13318500
N	-4.35118100	-0.55025200	-0.24175300
H	2.25943500	3.61529600	0.32696300
H	-0.26248000	4.51904400	0.50550000
H	-6.37387900	-0.97174600	-0.43598300
C	2.47965800	0.75660100	0.13672400
C	3.67407800	1.32954400	-0.35634400
C	2.48741700	-0.59100200	0.53957200
C	4.82988600	0.56947300	-0.44928200
H	3.70639200	2.35182800	-0.70978700
C	3.65624800	-1.33611100	0.44686600
H	1.61413800	-1.06602600	0.95992100
C	4.84025800	-0.77392000	-0.04847000
H	5.74308500	-1.36306200	-0.11838500
O	3.57520400	-2.62142300	0.87163900
O	5.92228600	1.19874700	-0.95096300
C	4.73658800	-3.43061600	0.81629100
H	4.45043400	-4.40512900	1.20425900
H	5.09176300	-3.54210500	-0.21242300
H	5.53697000	-3.01611400	1.43647800
C	7.12606200	0.46389300	-1.08536800
H	6.99597900	-0.38681200	-1.76084600
H	7.85806600	1.14824600	-1.50717300
H	7.48282800	0.10833000	-0.11424500

1bc, S_1 -1589.163247 a.u.

B	1.32167700	0.55835400	0.35400200
B	4.70098200	-1.41301200	-0.14232300
C	-0.96802400	-0.75923100	0.60043500
C	-1.30859700	-2.14177200	0.69902100
C	-0.15270900	-2.87704100	0.71449200
C	0.92665900	-1.95321900	0.58271900
C	2.27879200	-2.22904400	0.42022100
H	2.67131800	-3.23359300	0.46753100
C	3.81657500	0.82030600	-0.33079700
C	3.81064500	2.20268500	-0.52106400
H	2.88860200	2.75622800	-0.41379000
C	5.00874200	2.81055700	-0.84987600
H	5.04094600	3.88101400	-1.00693600
C	6.18794600	2.04229400	-0.98198700
H	7.12970100	2.50533900	-1.24114300
C	6.13274800	0.68671900	-0.77225600
F	0.88837400	1.37844800	-0.68442100
F	1.47605300	1.31445800	1.52638000
F	4.94231100	-2.24100800	-1.22959000
F	5.39156400	-1.87009400	0.97100800
N	0.41789300	-0.66473300	0.54293200
N	2.76244700	0.02214000	-0.00571400
N	3.16915300	-1.25052200	0.13883400
N	4.96277900	0.08562200	-0.44587900
H	-2.31547000	-2.51513000	0.77996500
H	-0.04375400	-3.94801900	0.78717700
H	6.99707200	0.03968400	-0.85035200
C	-1.89335900	0.33358600	0.61830500
C	-1.56526400	1.65366200	1.02376300
C	-3.25064500	0.15940100	0.25337700
C	-2.50075300	2.67967600	1.05514100
H	-0.55996800	1.86075600	1.35795900
C	-4.19814600	1.18753400	0.27270200
C	-3.82973000	2.47131300	0.67901600
H	-2.18734800	3.66116800	1.39000600
C	-5.43090500	0.59007400	-0.20618900
C	-5.11329500	-0.74401800	-0.47844700
H	-4.55183300	3.27894700	0.70208500
C	-6.73397700	1.04121200	-0.42857800
C	-6.02617100	-1.66634600	-0.96259800
C	-7.66912000	0.13449700	-0.91423000
H	-7.00895700	2.07018900	-0.22762400
C	-7.31988600	-1.19920400	-1.17717000
H	-5.74273100	-2.69170600	-1.16232000
H	-8.68671000	0.45992600	-1.09436900
H	-8.07236800	-1.88055800	-1.55590100
O	-3.79394400	-1.00207300	-0.20353600

1bd, S_1 -1515.175811 a.u.

B	-0.85806000	0.86313900	0.32815300
B	-3.95503000	-1.57080900	0.33776600
C	1.54563600	-0.06282000	0.89651000
C	2.04724100	-1.29666100	1.40530500
C	0.98528900	-2.12835700	1.65990600
C	-0.19148800	-1.42404500	1.26724800
C	-1.49902900	-1.88709800	1.17571600
H	-1.78963600	-2.87140200	1.51090800
C	-3.31641800	0.59886100	-0.49125400
C	-3.45558200	1.85450900	-1.08158800
H	-2.60904400	2.52516600	-1.12405000
C	-4.69438300	2.18715500	-1.60138800
H	-4.83792100	3.15270900	-2.06887400
C	-5.76961000	1.27213700	-1.52515100
H	-6.74121600	1.51771700	-1.93052700
C	-5.57480500	0.05509400	-0.92124700
H	-6.35533000	-0.68838800	-0.82195400
F	-1.17258600	1.85432800	1.27043600
F	-0.45375200	1.45219500	-0.86528300
F	-4.65372600	-1.76594200	1.52117200
F	-4.03569900	-2.70649500	-0.45728900
N	0.16718900	-0.15159000	0.84354300
N	-2.19841600	0.06679900	0.07777700
N	-2.46992800	-1.14432400	0.59323900
N	-4.36472600	-0.27443900	-0.40498300
H	3.09240400	-1.49854000	1.58407400
H	1.00364300	-3.13129000	2.05729800
C	2.33031900	1.10808400	0.58835400
C	1.86793500	2.39929700	0.93145400
C	3.63523600	0.97732800	0.02902000
C	2.68218800	3.50790400	0.79536300
H	0.88282100	2.50818300	1.36368100
C	4.44415200	2.11062800	-0.07650000
C	3.98468800	3.36672200	0.30373400
H	2.31406700	4.48376200	1.08929700
H	5.43120600	2.00552700	-0.51434200
H	4.62639300	4.23344500	0.19776500
C	4.12855000	-0.30072000	-0.53184600
C	5.41682500	-0.75999600	-0.23162500
C	3.33103400	-1.04908100	-1.41195500
C	5.88775700	-1.95152200	-0.77629900
H	6.03813400	-0.19120200	0.45248300
C	3.80634700	-2.23280500	-1.96341200
H	2.34150700	-0.68554100	-1.67028900
C	5.08403300	-2.69218200	-1.64140400
H	6.88070800	-2.30408400	-0.52187700
H	3.18293500	-2.79567100	-2.64846800
H	5.45233800	-3.61851900	-2.06673000

1be′, S_1 -1607.852055 a.u.

B	-0.00294800	0.60138800	1.13712300
B	3.49294400	1.08200600	-0.56506300
C	-2.14124900	1.65054100	0.04874700
C	-2.34244300	2.87263600	-0.64627000
C	-1.11738600	3.32558000	-1.08519100
C	-0.14409900	2.39593900	-0.61266800
C	1.24226100	2.38212600	-0.76345000
H	1.77660900	3.13671100	-1.32102900
C	2.38763800	-0.49122900	0.88879600
C	2.24662300	-1.59712100	1.73158100
H	1.29308900	-1.80544100	2.19390800
C	3.35630200	-2.39546500	1.93912700
H	3.27809400	-3.25839500	2.58809600
C	4.58707100	-2.09430600	1.31200800
H	5.45965700	-2.71349800	1.46557900
C	4.66820400	-0.99206800	0.50087800
H	5.57676400	-0.69352800	-0.00645100
F	0.09801000	1.34164100	2.32075900
F	-0.53551700	-0.65444200	1.40964500
F	4.32528700	2.10088900	-0.12078100
F	3.71603100	0.84196200	-1.91661200
N	-0.78480500	1.38150800	0.07599500
N	1.44325800	0.42771300	0.53913400
N	1.98881500	1.36898500	-0.26395300
N	3.58465500	-0.20181800	0.29704400
H	-3.31228600	3.30141200	-0.85122000
H	-0.90794900	4.19516400	-1.68859000
C	-3.19260300	0.85487800	0.58279200
C	-4.34651100	1.34777500	1.20498400
N	-3.26569000	-0.54373700	0.65661600
C	-5.09279800	0.25632700	1.68191800
H	-4.55444000	2.39696400	1.35232100
C	-4.41584300	-0.89191600	1.32971100
H	-6.02026800	0.29288700	2.23115400
H	-4.66206700	-1.93319100	1.46052900
C	-2.56778600	-1.51894200	-0.10359000
O	-2.69106200	-2.69602100	0.13120500
O	-1.88474000	-0.94936000	-1.06724400
C	-1.05088900	-1.71985700	-2.00378700
C	-1.96701300	-2.51203000	-2.92262200
H	-1.36055000	-3.02926400	-3.66976200
H	-2.53421600	-3.25535000	-2.36060900
H	-2.65786400	-1.84249500	-3.44033700
C	-0.06845200	-2.60860400	-1.25593900
H	0.66395100	-2.98590800	-1.97386500
H	0.46010800	-2.02660300	-0.49798400
H	-0.56271300	-3.45342700	-0.77857400
C	-0.30893000	-0.62953500	-2.76055100
H	0.26752900	-1.07613600	-3.57327800
H	-1.01510400	0.09172900	-3.17974900
H	0.37758100	-0.10293300	-2.09192100

1bf, S$_1$ -1758.609207 a.u.

B	0.54044200	-0.20152500	0.00015700
B	4.32787200	0.90738500	-0.00009800
C	-1.38743600	1.60753000	0.00007300
C	-1.40807000	3.04103800	0.00003600
C	-0.12000000	3.49695400	0.00005000
C	0.72660700	2.34891300	0.00009400
C	2.11643100	2.30023000	0.00010900
H	2.72212500	3.19427100	0.00012100
C	2.97590600	-1.08352100	0.00004300
C	2.66964600	-2.44783100	0.00004400
H	1.63652500	-2.76607800	0.00011000
C	3.72327200	-3.33939500	-0.00004300
H	3.52300100	-4.40336400	-0.00004800
C	5.05978100	-2.87569100	-0.00012000
H	5.89079500	-3.56674500	-0.00018000
C	5.29645500	-1.52543700	-0.00011300
H	6.29024800	-1.09638300	-0.00016200
F	0.18420200	-0.92576200	1.14222300
F	0.18417200	-0.92581600	-1.14187300
F	4.92848600	1.42568900	1.13816700
F	4.92804500	1.42556700	-1.13867900
N	-0.05592500	1.20757300	0.00012200
N	2.10352500	-0.04235800	0.00012100
N	2.77743400	1.12830700	0.00014000
N	4.26611400	-0.64426100	-0.00003200
H	-2.30094500	3.64576200	0.00002500
H	0.22457200	4.51930600	0.00002300
C	-2.54924000	0.80465500	0.00004700
C	-3.85347400	1.33166400	-0.00008800
C	-4.32543700	-0.96235900	0.00002300
C	-4.86172500	0.35367400	-0.00010800
H	-4.05985300	2.39316800	-0.00014600
C	-5.14553300	-2.08552700	0.00004500
C	-6.26994400	0.51837700	-0.00023600
C	-6.52395900	-1.89407600	-0.00007500
H	-4.72415000	-3.08367600	0.00014400
C	-7.07929600	-0.59799200	-0.00021700
H	-6.69349300	1.51622600	-0.00035300
H	-7.18046300	-2.75614200	-0.00006300
H	-8.15672600	-0.48336500	-0.00031700
S	-2.58995500	-0.94571600	0.00013600

1bg, S_1 -1397.593965 a.u.

B	-0.13611800	-0.23431800	-0.30255000
B	3.53915500	1.11631100	0.23489800
C	-2.18182200	1.35597700	-0.06288600
C	-2.34497000	2.76692100	-0.08117200
C	-1.11213800	3.34523100	0.10537900
C	-0.16255500	2.27999600	0.17360500
C	1.22184800	2.33806600	0.22992000
H	1.76192900	3.26774600	0.32902200
C	2.37179800	-0.93529500	-0.25837100
C	2.18401800	-2.28518500	-0.55468400
H	1.18513700	-2.67103600	-0.70159600
C	3.31143200	-3.08275900	-0.64853300
H	3.20566000	-4.13485300	-0.87907600
C	4.59464100	-2.53056300	-0.44535500
H	5.48279000	-3.14276700	-0.51443400
C	4.71439000	-1.19103700	-0.15888000
F	-0.44718100	-1.31341400	0.52143400
F	-0.39198200	-0.56872300	-1.63006600
F	4.00556300	1.43865700	1.49901300
F	4.16319100	1.89565700	-0.72626200
N	-0.83985700	1.07047600	0.09505700
N	1.41500900	0.02708600	-0.13464200
N	1.97808800	1.21461800	0.13691500
N	3.61606600	-0.40600300	-0.06786900
H	-3.29926300	3.26621100	-0.15927300
H	-0.87211300	4.39445100	0.18265900
H	5.66709900	-0.70326100	0.00279600
C	-3.24690500	0.38751500	-0.18881500
C	-3.38363700	-0.77502300	0.61571000
C	-4.22510000	0.63237600	-1.17953900
C	-4.40145200	-1.69245200	0.34012400
C	-5.22951800	-0.28427500	-1.43085000
H	-4.14103100	1.52613900	-1.78750000
C	-5.30856300	-1.46890900	-0.68497600
H	-4.48614600	-2.56394500	0.97948300
H	-5.94918500	-0.08863300	-2.21689600
H	-6.09216600	-2.18890400	-0.88760200
C	-2.61906100	-0.93692300	1.87006700
O	-2.71811400	-1.90505700	2.60114500
H	-1.97889800	-0.08543900	2.15376700

1bh, S_1 -1621.238858 a.u.

B	-0.76621700	-0.13378900	0.19975500
B	-4.61933800	0.70198600	-0.03428600
C	1.02753200	1.80361200	0.03551300
C	0.95052900	3.22685600	-0.03904100
C	-0.36450200	3.59946400	0.03833000
C	-1.12861600	2.39570900	0.12697900
C	-2.50726000	2.24792600	0.10661700
H	-3.17753400	3.09411800	0.09329400
C	-3.12660100	-1.19106000	-0.07827000
C	-2.72006100	-2.52503800	-0.11010700
H	-1.66781700	-2.76857900	-0.06759000
C	-3.70505700	-3.49280800	-0.20111300
H	-3.42779800	-4.53861100	-0.23001300
C	-5.06667700	-3.12215100	-0.25635500
H	-5.84559300	-3.86805100	-0.32946300
C	-5.40449500	-1.79049500	-0.21214000
F	-0.22676100	-0.91328800	-0.81778700
F	-0.55273700	-0.74710200	1.44091400
F	-5.19574400	1.24937200	-1.16957500
F	-5.30278800	1.10773100	1.10065100
N	-0.26207000	1.31243800	0.15171300
N	-2.33572600	-0.08541000	0.00741400
N	-3.08711100	1.02446200	0.05438900
N	-4.44594900	-0.83925900	-0.12032600
H	1.80209000	3.88616100	-0.10166000
H	-0.77946400	4.59529600	0.02494000
H	-6.42757000	-1.43830500	-0.24433500
C	2.25028300	1.04594900	0.01627000
C	3.40842500	1.64852200	-0.54081700
C	2.37577400	-0.25997600	0.54420500
C	4.61568800	0.98059200	-0.57611800
H	3.34226200	2.63515400	-0.98136000
C	3.58946500	-0.92483400	0.51071600
H	1.53036900	-0.74175900	1.01152200
C	4.70946000	-0.31291800	-0.05253300
H	5.48379700	1.45074600	-1.02267600
H	3.66877800	-1.92169400	0.92690500
C	6.03481100	-1.00655700	-0.04833100
F	6.80805000	-0.61551400	0.98804800
F	6.74387300	-0.74718200	-1.16106800
F	5.91611500	-2.33933300	0.04747600

1bi, S_1 -1605.119875 a.u.

B	0.19928900	-0.51061400	0.00045400
B	-3.29708600	1.31955600	-0.00020600
C	2.43893400	0.88258500	0.00008100
C	2.74883200	2.27713900	0.00008300
C	1.57733800	2.98568800	0.00016200
C	0.51672300	2.02984300	0.00011100
C	-0.85640000	2.25370400	0.00016500
H	-1.27949200	3.24688800	0.00035800
C	-2.36474000	-0.89993600	0.00010900
C	-2.33226600	-2.29559700	0.00016900
H	-1.38170600	-2.81039300	0.00030900
C	-3.54113100	-2.96512800	0.00001000
H	-3.55409600	-4.04755400	0.00006200
C	-4.75925800	-2.24560600	-0.00019100
H	-5.71054900	-2.75882900	-0.00034200
C	-4.72688300	-0.87461900	-0.00020400
H	-5.61802300	-0.26011300	-0.00031600
F	0.40558500	-1.29592900	-1.13981700
F	0.40577800	-1.29516900	1.14120400
F	-3.77986100	1.94918900	-1.13889600
F	-3.78076600	1.94979700	1.13766700
N	1.05495400	0.75532500	0.00005200
N	-1.30397400	-0.04803400	0.00027500
N	-1.73272900	1.22883200	0.00034400
N	-3.54355400	-0.21147700	-0.00003700
H	3.74982600	2.68310400	-0.00015900
H	1.44544800	4.05645500	0.00019000
C	3.38980800	-0.16394300	-0.00000300
C	3.24946100	-1.55755200	-0.00010600
S	5.09176300	0.25765400	-0.00017000
C	4.48064900	-2.23258500	-0.00039000
H	2.29327000	-2.05581000	-0.00014000
C	5.56327600	-1.38018800	-0.00051100
H	4.57853700	-3.30954900	-0.00053200
H	6.61198000	-1.63773200	-0.00072800

1bj, S$_1$ -1796.898227 a.u.

B	-1.12321700	-0.71081900	0.04433900
B	-4.41162000	1.45796200	-0.06502700
C	1.17800200	0.42509700	0.45386600
C	1.68042000	1.74254100	0.54517400
C	0.60449000	2.60379100	0.42586600
C	-0.56171400	1.80071100	0.26576500
C	-1.90474100	2.15367700	0.13526500
H	-2.24352500	3.17845100	0.12934300
C	-3.71268400	-0.84582900	-0.09072900
C	-3.81758200	-2.23207900	-0.15825900
H	-2.92400100	-2.83996600	-0.11963800
C	-5.08585000	-2.77847100	-0.27241300
H	-5.20638800	-3.85245700	-0.32742200
C	-6.22056800	-1.93389800	-0.31652200
H	-7.21691000	-2.34455100	-0.40367900
C	-6.05393600	-0.57372400	-0.25052200
H	-6.87923100	0.12591200	-0.28332600
F	-0.88491800	-1.34909000	-1.17520000
F	-1.03679700	-1.64568300	1.07892400
F	-4.74618900	2.17853000	-1.20530500
F	-4.90111500	2.10485400	1.06343200
N	-0.18313500	0.46987900	0.27945700
N	-2.57261400	-0.10485000	0.01767800
N	-2.86730000	1.20679100	0.03231200
N	-4.81241400	-0.03240800	-0.14045400
H	2.71733200	1.99765000	0.70100000
H	0.61643000	3.68205600	0.46278500
S	1.99514200	-1.08065300	0.59895700
C	3.64804500	-0.62110700	0.18102600
C	3.93566700	0.01721900	-1.03486500
C	4.68228900	-0.96370800	1.04685900
C	5.24273200	0.31824600	-1.36107300
H	3.13062000	0.26923200	-1.71602300
C	6.00449900	-0.67159700	0.71713800
H	4.45927900	-1.45488600	1.98703500
C	6.28732700	-0.02357200	-0.48748200
H	5.48798300	0.80906300	-2.29503700
H	6.79319000	-0.94328300	1.40576000
O	7.53107100	0.30797400	-0.89316000
C	8.62507300	-0.02030800	-0.05119300
H	9.51975900	0.32782800	-0.56070700
H	8.53872100	0.48166500	0.91646600
H	8.69069800	-1.10110100	0.10181800

1ca, S$_1$ -6752.999738 a.u.

B	-1.27781900	-1.42045500	-0.01394600
B	-3.25415300	1.98442800	0.11017300
C	1.32363200	-1.41999000	0.02157000
C	2.35770100	-0.51653900	0.07752600
C	1.80048000	0.79451200	0.07111000
C	0.36316200	0.58648100	0.03814500
C	-0.69465700	1.49330600	0.10082900
H	-0.56308600	2.55787800	0.20897200
C	-3.64909000	-0.38777400	-0.00317900
C	-4.36367700	-1.58864100	-0.06379400
H	-3.83291000	-2.52977800	-0.10299500
C	-5.74161500	-1.50997800	-0.07095100
H	-6.32874400	-2.41838400	-0.11637800
C	-6.39118900	-0.25598800	-0.01971800
H	-7.46961800	-0.18539400	-0.02537300
C	-5.63356400	0.88722200	0.03903700
H	-6.06076800	1.88084800	0.08165300
F	-1.48973400	-2.16563500	-1.17231600
F	-1.48402600	-2.22110500	1.10786800
F	-3.32789900	2.82510000	-0.99022400
F	-3.34316100	2.71865300	1.28354600
N	0.11219500	-0.76341900	-0.00035700
N	-2.30063500	-0.23463900	0.01460300
N	-1.98148600	1.07710100	0.07801900
N	-4.28170800	0.81719700	0.04799700
Br	4.15427800	-1.01104000	0.02165400
Br	1.43781900	-3.26071100	-0.06145400
C	2.47305100	2.04381800	0.08094400
C	3.78942200	2.33122400	0.46463400
C	4.13782500	3.67734600	0.28582400
H	4.45178500	1.59079200	0.88622500
C	3.10474800	4.43296400	-0.22809000
H	5.10519100	4.09124200	0.53412800
H	3.10135300	5.48865700	-0.45462900
S	1.70254500	3.50756200	-0.49404600

5. Crystallographic Data and Structure Parameters

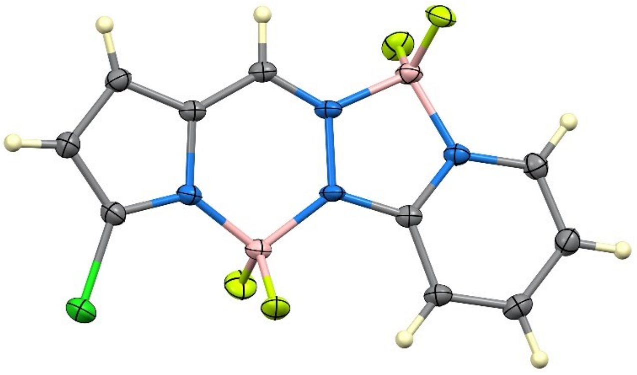

(1a)

Crystal data

$C_{10}H_7B_2ClF_4N_4$	$F(000) = 632$
$M_r = 316.27$	$D_x = 1.714$ Mg m^{-3}
Monoclinic, $P2_1/n$	Cu $K\alpha$ radiation, $\lambda = 1.54184$ Å
$a = 7.5933$ (2) Å	Cell parameters from 9869 reflections
$b = 16.9225$ (4) Å	$\theta = 5.3–78.8°$
$c = 9.6148$ (2) Å	$\mu = 3.24$ mm^{-1}
$\beta = 97.301$ (1)°	$T = 110$ K
$V = 1225.46$ (5) Å^3	Plate, blue
$Z = 4$	0.18 × 0.10 × 0.01 mm

Data collection

Bruker D8 Venture DUO with Photon III C14 diffractometer	2381 reflections with $I > 2\sigma(I)$
Radiation source: IμS 3.0 microfocus	$R_{int} = 0.055$
φ and ω scans	$\theta_{max} = 79.6°$, $\theta_{min} = 5.2°$
Absorption correction: multi-scan *SADABS* (Krause *et al.*, 2015)	$h = -9 – 9$
$T_{min} = 0.695$, $T_{max} = 0.968$	$k = -21 – 21$
25533 measured reflections	$l = -11 – 12$
2643 independent reflections	

Refinement

Refinement on F^2	0 restraints
Least-squares matrix: full	Hydrogen site location: inferred from neighbouring

	sites
$R[F^2 > 2\sigma(F^2)] = 0.034$	H-atom parameters constrained
$wR(F^2) = 0.091$	$w = 1/[\sigma^2(F_o^2) + (0.0458P)^2 + 0.6449P]$ where $P = (F_o^2 + 2F_c^2)/3$
$S = 1.08$	$(\Delta/\sigma)_{max} < 0.001$
2643 reflections	$\Delta\rho_{max} = 0.44$ e Å^{-3}
190 parameters	$\Delta\rho_{min} = -0.44$ e Å^{-3}

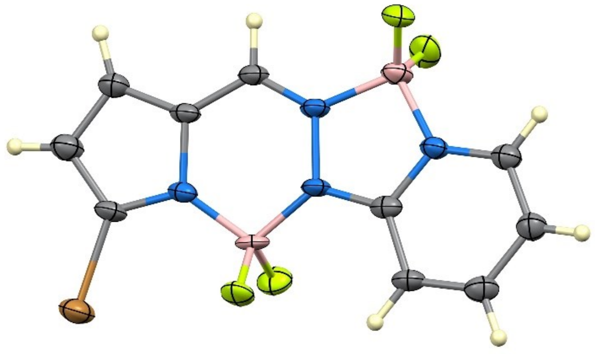

(1b)

Crystal data

$C_{10.38}H_{8.02}B_2BrF_4N_4$	$F(000) = 717$
$M_r = 366.35$	$D_x = 1.943$ Mg m^{-3}
Monoclinic, $P2_1/n$	Cu $K\alpha$ radiation, $\lambda = 1.54184$ Å
$a = 7.6739$ (8) Å	Cell parameters from 7169 reflections
$b = 17.451$ (2) Å	$\theta = 5.4–78.4°$
$c = 9.4347$ (12) Å	$\mu = 4.95$ mm^{-1}
$\beta = 97.592$ (7)°	$T = 110$ K
$V = 1252.4$ (3) Å^3	Plate, light blue
$Z = 4$	0.12 × 0.10 × 0.01 mm

Data collection

Bruker D8 Venture DUO with Photon III C14 diffractometer	2306 reflections with $I > 2\sigma(I)$
Radiation source: IμS 3.0 microfocus	$R_{int} = 0.072$
ϕ and ω scans	$\theta_{max} = 80.3°$, $\theta_{min} = 5.1°$
Absorption correction: multi-scan *SADABS* (Krause *et al.*, 2015)	$h = -9 – 9$
$T_{min} = 0.691$, $T_{max} = 0.952$	$k = -22 – 22$

19011 measured reflections	$l = -11 – 11$
2683 independent reflections	

Refinement

Refinement on F^2	Hydrogen site location: inferred from neighbouring sites
Least-squares matrix: full	H-atom parameters constrained
$R[F^2 > 2\sigma(F^2)] = 0.062$	$w = 1/[\sigma^2(F_o^2) + (0.0805P)^2 + 6.391P]$ where $P = (F_o^2 + 2F_c^2)/3$
$wR(F^2) = 0.177$	$(\Delta/\sigma)_{max} < 0.001$
$S = 1.07$	$\Delta\rho_{max} = 1.23$ e Å^{-3}
2683 reflections	$\Delta\rho_{min} = -1.30$ e Å^{-3}
201 parameters	Extinction correction: *SHELXL2019*/1 (Sheldrick 2019), $Fc^*=kFc[1+0.001xFc^2\lambda^3/\sin(2\theta)]^{-1/4}$
6 restraints	Extinction coefficient: 0.0045 (6)

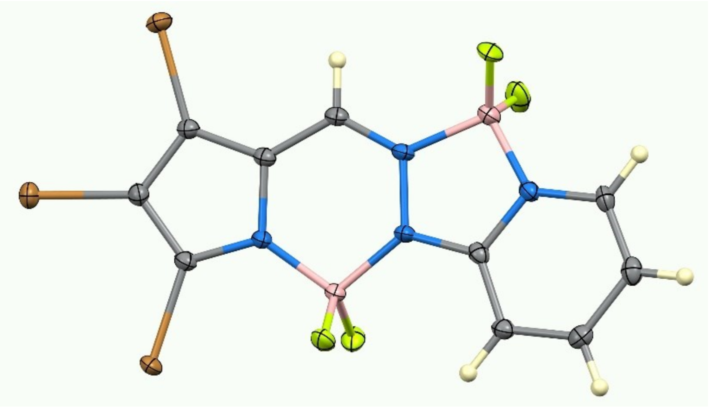

(1c)

Crystal data

$C_{10}H_5B_2Br_3F_4N_4$	$F(000) = 976$
$M_r = 518.53$	$D_x = 2.402$ Mg m^{-3}
Monoclinic, $P2_1/c$	Cu $K\alpha$ radiation, $\lambda = 1.54184$ Å
$a = 10.8096$ (2) Å	Cell parameters from 9636 reflections
$b = 14.8493$ (3) Å	$\theta = 5.1–79.8°$
$c = 8.9399$ (2) Å	$\mu = 10.90$ mm^{-1}

β = 92.471 (2)°	T = 100 K
V = 1433.65 (5) Å^3	Plate, orange
Z = 4	0.11 × 0.10 × 0.03 mm

Data collection

Bruker D8 Venture DUO with Photon III C14 diffractometer	2855 reflections with $I > 2\sigma(I)$
Radiation source: IμS 3.0 microfocus	R_{int} = 0.044
ϕ and ω scans	θ_{max} = 79.9°, θ_{min} = 4.1°
Absorption correction: multi-scan *SADABS* (Krause *et al.*, 2015)	h = -13 – 13
T_{min} = 0.506, T_{max} = 0.736	k = -16 – 18
37464 measured reflections	l = -11 – 11
3115 independent reflections	

Refinement

Refinement on F^2	0 restraints
Least-squares matrix: full	Hydrogen site location: inferred from neighbouring sites
$R[F^2 > 2\sigma(F^2)]$ = 0.021	H-atom parameters constrained
$wR(F^2)$ = 0.053	$w = 1/[\sigma^2(F_o^2) + (0.0249P)^2 + 2.2437P]$ where $P = (F_o^2 + 2F_c^2)/3$
S = 1.02	$(\Delta/\sigma)_{max}$ = 0.001
3115 reflections	$\Delta\rho_{max}$ = 0.60 e Å^{-3}
208 parameters	$\Delta\rho_{min}$ = -0.40 e Å^{-3}

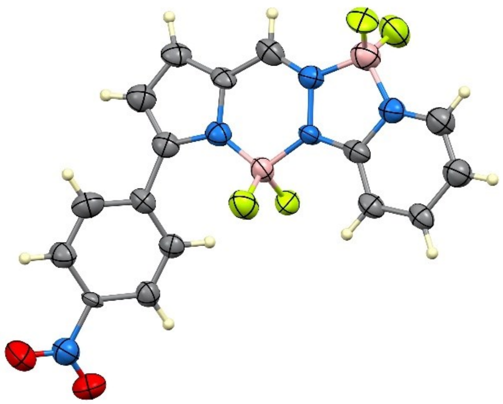

(1ba)

Crystal data

$C_{16}H_{11}B_2F_4N_5O_2$	$F(000) = 816$
$M_r = 402.92$	$D_x = 1.612$ Mg m^{-3}
Monoclinic, $P2_1/c$	Cu $K\alpha$ radiation, $\lambda = 1.54184$ Å
$a = 12.9351$ (13) Å	Cell parameters from 697 reflections
$b = 7.2631$ (10) Å	$\theta = 3.5$–$46.6°$
$c = 17.949$ (3) Å	$\mu = 1.20$ mm^{-1}
$\beta = 100.068$ (10)°	$T = 100$ K
$V = 1660.4$ (4) Å^3	Fragment, yellow
$Z = 4$	0.07 × 0.04 × 0.02 mm

Data collection

Bruker D8 Venture DUO with Photon III C14 diffractometer	1166 reflections with $I > 2\sigma(I)$
Radiation source: IμS 3.0 microfocus	$R_{int} = 0.193$
ϕ and ω scans	$\theta_{max} = 54.5°$, $\theta_{min} = 2.5°$
Absorption correction: multi-scan *SADABS* (Krause *et al.*, 2015)	$h = -13 – 13$
$T_{min} = 0.812$, $T_{max} = 0.976$	$k = -5 – 7$
11590 measured reflections	$l = -18 – 18$
2040 independent reflections	

Refinement

Refinement on F^2	138 restraints
Least-squares matrix: full	Hydrogen site location: inferred from neighbouring sites

$R[F^2 > 2\sigma(F^2)] = 0.165$	H-atom parameters constrained
$wR(F^2) = 0.471$	$w = 1/[\sigma^2(F_o^2) + (0.2P)^2]$ where $P = (F_o^2 + 2F_c^2)/3$
$S = 1.64$	$(\Delta/\sigma)_{max} < 0.001$
2040 reflections	$\Delta\rho_{max} = 1.06$ e Å^{-3}
263 parameters	$\Delta\rho_{min} = -0.61$ e Å^{-3}

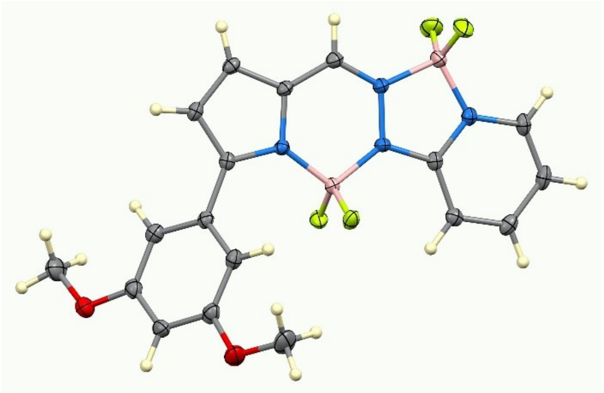

(1bb)

Crystal data

$C_{18}H_{16}B_2F_4N_4O_2$	$F(000) = 1712$
$M_r = 417.97$	$D_x = 1.532$ Mg m^{-3}
Monoclinic, *I*2/*a*	Cu *K*α radiation, λ = 1.54184 Å
a = 12.1978 (11) Å	Cell parameters from 9816 reflections
b = 6.6333 (6) Å	θ = 5.9–79.4°
c = 44.890 (4) Å	μ = 1.10 mm^{-1}
β = 93.782 (7)°	*T* = 100 K
V = 3624.2 (6) Å^3	Plate, yellow
Z = 8	0.23 × 0.14 × 0.03 mm

Data collection

Bruker D8 Venture DUO with Photon III C14 diffractometer	3739 reflections with $I > 2\sigma(I)$
Radiation source: IμS 3.0 microfocus	$R_{int} = 0.050$
ϕ and ω scans	$\theta_{max} = 79.7°$, $\theta_{min} = 4.0°$
Absorption correction: multi-scan *SADABS* (Krause *et al.*, 2015)	*h* = -15 – 15
$T_{min} = 0.790$, $T_{max} = 0.968$	*k* = -8 – 8
69829 measured reflections	*l* = -57 – 57
3933 independent reflections	

Refinement

Refinement on F^2	0 restraints
Least-squares matrix: full	Hydrogen site location: inferred from neighbouring sites
$R[F^2 > 2\sigma(F^2)] = 0.059$	H-atom parameters constrained

$wR(F^2) = 0.169$	$w = 1/[\sigma^2(F_o^2) + (0.0594P)^2 + 15.7316P]$ where $P = (F_o^2 + 2F_c^2)/3$
$S = 1.14$	$(\Delta/\sigma)_{max} < 0.001$
3933 reflections	$\Delta\rho_{max} = 0.65$ e Å^{-3}
273 parameters	$\Delta\rho_{min} = -0.29$ e Å^{-3}

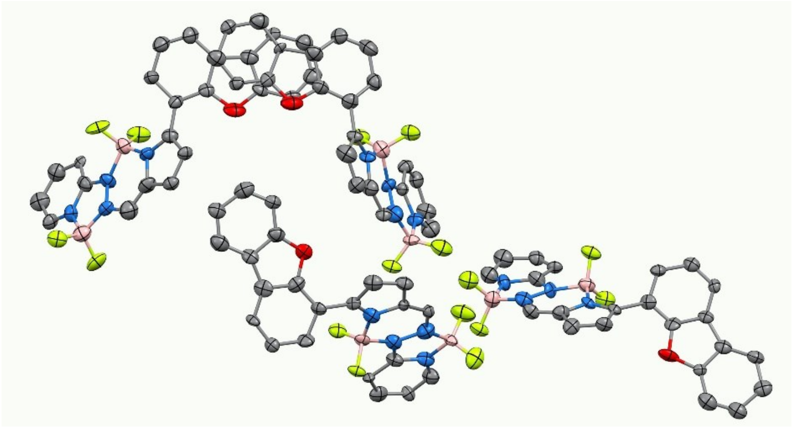

(1bc)

Crystal data

$C_{22}H_{14}B_2F_4N_4O$	$D_x = 1.326$ Mg m^{-3}
$M_r = 447.99$	Cu $K\alpha$ radiation, $\lambda = 1.54184$ Å
Orthorhombic, $Pca2_1$	Cell parameters from 2690 reflections
$a = 33.363$ (2) Å	$\theta = 3.5$–$46.3°$
$b = 7.0069$ (4) Å	$\mu = 0.90$ mm^{-1}
$c = 38.391$ (3) Å	$T = 100$ K
$V = 8974.7$ (10) Å^3	Needle, yellow
$Z = 16$	0.22 × 0.08 × 0.02 mm
$F(000) = 3648$	

Data collection

Bruker D8 Venture DUO with Photon III C14 diffractometer	9393 reflections with $I > 2\sigma(I)$
Radiation source: IµS 3.0 microfocus	$R_{int} = 0.235$
ϕ and ω scans	$\theta_{max} = 59.1°$, $\theta_{min} = 2.7°$
Absorption correction: multi-scan *SADABS* (Krause *et al.*, 2015)	$h = -37 - 37$

T_{min} = 0.848, T_{max} = 0.982	k = -7 – 7
105335 measured reflections	l = -42 – 42
12895 independent reflections	

Refinement

Refinement on F^2	H-atom parameters constrained
Least-squares matrix: full	$w = 1/[\sigma^2(F_o^2) + (0.2P)^2]$ where $P = (F_o^2 + 2F_c^2)/3$
$R[F^2 > 2\sigma(F^2)]$ = 0.177	$(\Delta/\sigma)_{max}$ = 0.001
$wR(F^2)$ = 0.441	$\Delta\rho_{max}$ = 1.55 e Å^{-3}
S = 1.68	$\Delta\rho_{min}$ = -1.21 e Å^{-3}
12895 reflections	Extinction correction: *SHELXL2019*/1 (Sheldrick 2019), $Fc^* = kFc[1+0.001xFc^2\lambda^3/\sin(2\theta)]^{-1/4}$
1191 parameters	Extinction coefficient: 0.0153 (17)
1285 restraints	Absolute structure: Refined as an inversion twin.
Hydrogen site location: inferred from neighbouring sites	Absolute structure parameter: 0.4 (6)

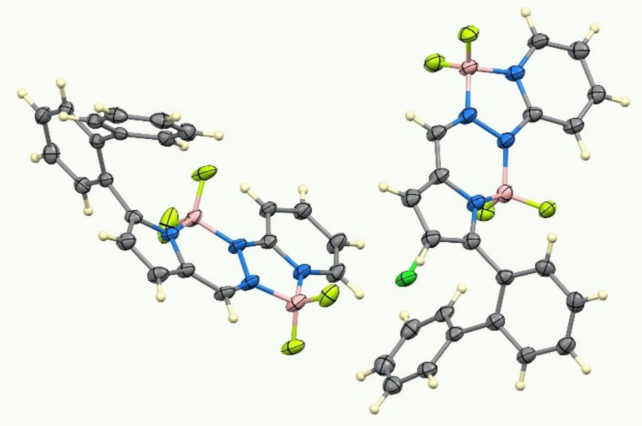

(1bd)

Crystal data

$C_{22}H_{16}B_2F_4N_4 \cdot C_{22}H_{15.86}B_2Cl_{0.14}F_4N_4$	D_x = 1.475 Mg m^{-3}
M_r = 872.82	Cu $K\alpha$ radiation, λ = 1.54184 Å
Orthorhombic, $Pna2_1$	Cell parameters from 6478 reflections
a = 29.9600 (9) Å	θ = 4.2–66.5°
b = 8.8220 (3) Å	μ = 1.06 mm^{-1}
c = 14.8732 (6) Å	T = 100 K

V = 3931.1 (2) Å^3	Needle, yellow
Z = 4	0.20 × 0.03 × 0.03 mm
F(000) = 1785	

Data collection

Bruker D8 Venture DUO with Photon III C14 diffractometer	6777 reflections with $I > 2\sigma(I)$
Radiation source: IμS 3.0 microfocus	R_{int} = 0.075
φ and ω scans	θ_{max} = 70.3°, θ_{min} = 4.2°
Absorption correction: multi-scan *SADABS* (Krause *et al.*, 2015)	h = -36 – 36
T_{min} = 0.814, T_{max} = 0.969	k = -10 – 10
59676 measured reflections	l = -18 – 18
7468 independent reflections	

Refinement

Refinement on F^2	H-atom parameters constrained
Least-squares matrix: full	$w = 1/[\sigma^2(F_o^2) + (0.0599P)^2 + 1.2074P]$ where $P = (F_o^2 + 2F_c^2)/3$
$R[F^2 > 2\sigma(F^2)]$ = 0.040	$(\Delta/\sigma)_{max} < 0.001$
$wR(F^2)$ = 0.108	$\Delta\rho_{max}$ = 0.41 e Å^{-3}
S = 1.03	$\Delta\rho_{min}$ = -0.29 e Å^{-3}
7468 reflections	Extinction correction: *SHELXL2019*/1 (Sheldrick 2019), $Fc^*=kFc[1+0.001xFc^2\lambda^3/\sin(2\theta)]^{-1/4}$
589 parameters	Extinction coefficient: 0.00027 (9)
1 restraint	Absolute structure: Refined as an inversion twin.
Hydrogen site location: inferred from neighbouring sites	Absolute structure parameter: 0.46 (10)

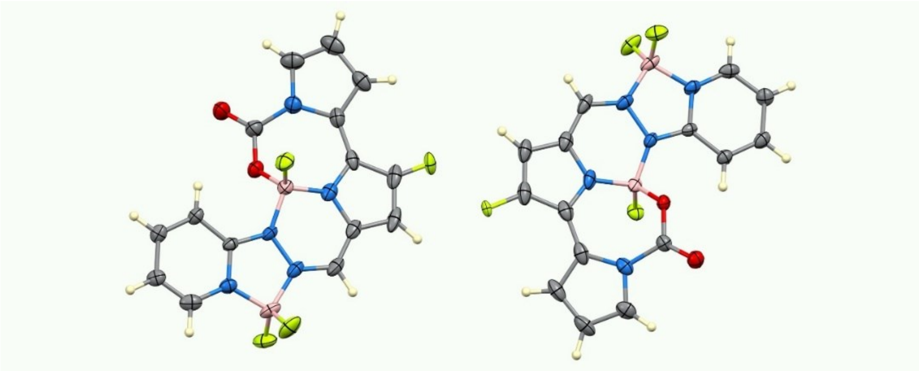

(1be')

Crystal data

0.5($C_{15}H_{9.67}B_2F_{3.33}N_5O_2$)·0.5($C_{15}H_{9.83}B_2F_{3.17}N_5O_2$)	$F(000) = 760$
$M_r = 375.41$	$D_x = 1.638$ Mg m^{-3}
Monoclinic, $P2_1$	Cu $K\alpha$ radiation, $\lambda = 1.54184$ Å
$a = 7.6446$ (3) Å	Cell parameters from 8614 reflections
$b = 22.0913$ (8) Å	$\theta = 4.0–69.0°$
$c = 9.1896$ (3) Å	$\mu = 1.19$ mm^{-1}
$\beta = 101.202$ (2)°	$T = 100$ K
$V = 1522.36$ (10) Å^3	Lath, orange
$Z = 4$	0.30 × 0.06 × 0.03 mm

Data collection

Bruker D8 Venture DUO with Photon III C14 diffractometer	4672 reflections with $I > 2\sigma(I)$
Radiation source: IμS 3.0 microfocus	$R_{int} = 0.059$
φ and ω scans	$\theta_{max} = 69.7°$, $\theta_{min} = 4.0°$
Absorption correction: multi-scan *SADABS* (Krause *et al.*, 2015)	$h = -9 – 9$
$T_{min} = 0.750$, $T_{max} = 0.965$	$k = -26 – 26$
21026 measured reflections	$l = -10 – 11$
5479 independent reflections	

Refinement

Refinement on F^2	Hydrogen site location: inferred from neighbouring sites
Least-squares matrix: full	H-atom parameters constrained
$R[F^2 > 2\sigma(F^2)] = 0.060$	$w = 1/[\sigma^2(F_o^2) + (0.0815P)^2 + 1.2661P]$

	where $P = (F_o^2 + 2F_c^2)/3$
$wR(F^2) = 0.156$	$(\Delta/\sigma)_{max} < 0.001$
$S = 1.07$	$\Delta\rho_{max} = 0.44$ e Å^{-3}
5479 reflections	$\Delta\rho_{min} = -0.27$ e Å^{-3}
508 parameters	Absolute structure: Refined as an inversion twin.
13 restraints	Absolute structure parameter: 0.5 (3)

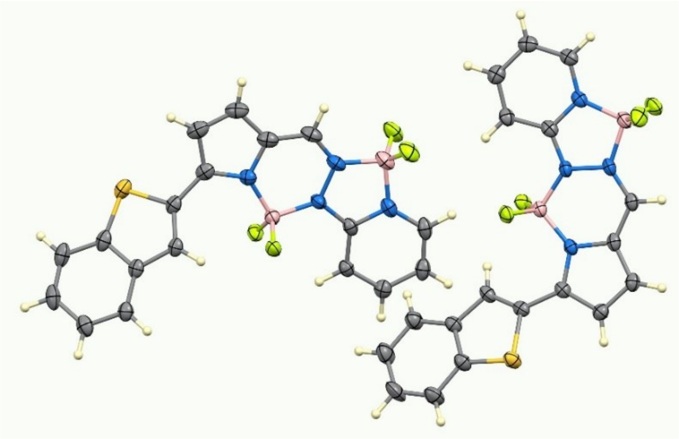

(1bf)

Crystal data

$C_{18}H_{12}B_2F_4N_4S$	$D_x = 1.569$ Mg m^{-3}
$M_r = 414.00$	Cu $K\alpha$ radiation, $\lambda = 1.54184$ Å
Orthorhombic, *Pbca*	Cell parameters from 9993 reflections
$a = 12.6008$ (4) Å	$\theta = 4.7$–$74.2°$
$b = 20.0837$ (7) Å	$\mu = 2.14$ mm^{-1}
$c = 27.7002$ (10) Å	$T = 100$ K
$V = 7010.1$ (4) Å^3	Needle, orange
$Z = 16$	0.21 × 0.05 × 0.02 mm
$F(000) = 3360$	

Data collection

Bruker D8 Venture DUO with Photon III C14 diffractometer	5734 reflections with $I > 2\sigma(I)$
Radiation source: IμS 3.0 microfocus	$R_{int} = 0.099$
ϕ and ω scans	$\theta_{max} = 74.6°$, $\theta_{min} = 3.2°$
Absorption correction: multi-scan	$h = -15 – 14$

SADABS (Krause *et al.*, 2015)	
T_{min} = 0.813, T_{max} = 0.959	k = -25 – 24
96802 measured reflections	l = -34 – 34
7173 independent reflections	

Refinement

Refinement on F^2	0 restraints
Least-squares matrix: full	Hydrogen site location: inferred from neighbouring sites
$R[F^2 > 2\sigma(F^2)]$ = 0.036	H-atom parameters constrained
$wR(F^2)$ = 0.096	$w = 1/[\sigma^2(F_o^2) + (0.0464P)^2 + 2.7556P]$ where $P = (F_o^2 + 2F_c^2)/3$
S = 1.03	$(\Delta/\sigma)_{max}$ = 0.001
7173 reflections	$\Delta\rho_{max}$ = 0.48 e Å^{-3}
523 parameters	$\Delta\rho_{min}$ = -0.38 e Å^{-3}

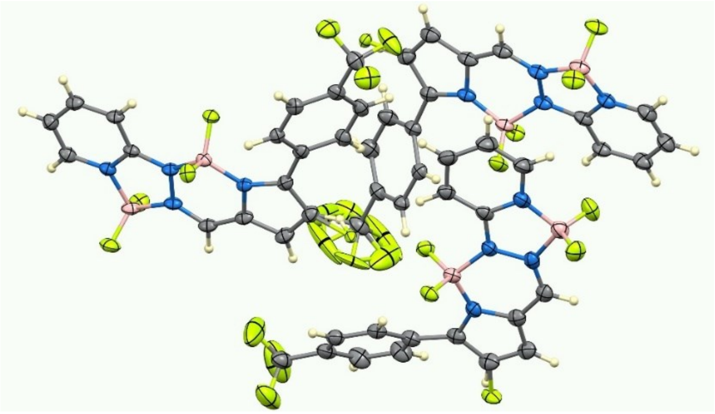

(1bh)

Crystal data

$C_{17}H_{10.80}B_2F_{7.20}N_4$	$F(000) = 5175$
$M_r = 429.59$	$D_x = 1.651$ Mg m^{-3}
Monoclinic, $C2/c$	Cu $K\alpha$ radiation, $\lambda = 1.54184$ Å
$a = 25.2362$ (5) Å	Cell parameters from 9962 reflections
$b = 15.1808$ (3) Å	$\theta = 3.4–79.7°$
$c = 28.0546$ (6) Å	$\mu = 1.37$ mm^{-1}
$\beta = 105.263$ (2)°	$T = 100$ K
$V = 10368.8$ (4) Å^3	Needle, yellow
$Z = 24$	0.15 × 0.09 × 0.05 mm

Data collection

Bruker D8 Venture DUO with Photon III C14 diffractometer	8988 reflections with $I > 2\sigma(I)$
Radiation source: IμS 3.0 microfocus	$R_{int} = 0.065$
ϕ and ω scans	$\theta_{max} = 80.3°$, $\theta_{min} = 3.3°$
Absorption correction: multi-scan *SADABS* (Krause *et al.*, 2015)	$h = -31 – 32$
$T_{min} = 0.823$, $T_{max} = 0.935$	$k = -18 – 18$
58707 measured reflections	$l = -35 – 30$
11072 independent reflections	

Refinement

Refinement on F^2	30 restraints
Least-squares matrix: full	Hydrogen site location: inferred from neighbouring sites

$R[F^2 > 2\sigma(F^2)] = 0.060$	H-atom parameters constrained
$wR(F^2) = 0.154$	$w = 1/[\sigma^2(F_o^2) + (0.0615P)^2 + 28.9193P]$ where $P = (F_o^2 + 2F_c^2)/3$
$S = 1.04$	$(\Delta/\sigma)_{max} < 0.001$
11072 reflections	$\Delta\rho_{max} = 1.03$ e Å^{-3}
869 parameters	$\Delta\rho_{min} = -0.46$ e Å^{-3}

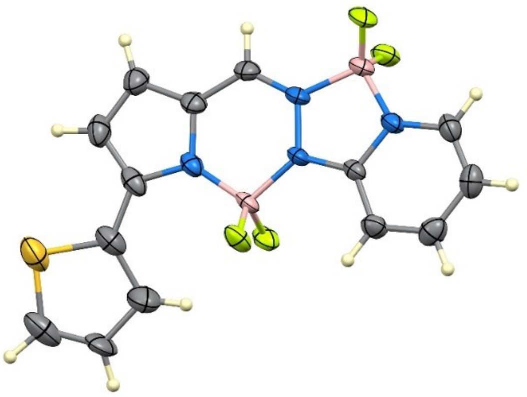

(1bi)

Crystal data

$C_{13.39}H_{9.54}B_2Br_{0.15}F_4N_4S_{0.85}$	$F(000) = 731$
$M_r = 363.45$	$D_x = 1.651$ Mg m^{-3}
Monoclinic, $P2_1/c$	Cu $K\alpha$ radiation, $\lambda = 1.54184$ Å
$a = 7.5097$ (2) Å	Cell parameters from 9892 reflections
$b = 10.2484$ (2) Å	$\theta = 4.3–78.6°$
$c = 18.9965$ (4) Å	$\mu = 2.73$ mm^{-1}
$\beta = 90.401$ (1)°	$T = 100$ K
$V = 1461.98$ (6) Å^3	Plate, yellow
$Z = 4$	0.24 × 0.11 × 0.03 mm

Data collection

Bruker D8 Venture DUO with Photon III C14 diffractometer	2933 reflections with $I > 2\sigma(I)$
Radiation source: IμS 3.0 microfocus	$R_{int} = 0.057$
ϕ and ω scans	$\theta_{max} = 79.4°$, $\theta_{min} = 4.7°$
Absorption correction: multi-scan *SADABS* (Krause *et al.*, 2015)	$h = -9 – 9$

T_{min} = 0.698, T_{max} = 0.923	k = -11 – 12
31788 measured reflections	l = -23 – 23
3149 independent reflections	

Refinement

Refinement on F^2	0 restraints
Least-squares matrix: full	Hydrogen site location: inferred from neighbouring sites
$R[F^2 > 2\sigma(F^2)]$ = 0.081	H-atom parameters constrained
$wR(F^2)$ = 0.197	$w = 1/[\sigma^2(F_o^2) + (0.0514P)^2 + 5.1397P]$ where $P = (F_o^2 + 2F_c^2)/3$
S = 1.14	$(\Delta/\sigma)_{max}$ < 0.001
3149 reflections	$\Delta\rho_{max}$ = 1.00 e Å^{-3}
236 parameters	$\Delta\rho_{min}$ = -0.62 e Å^{-3}

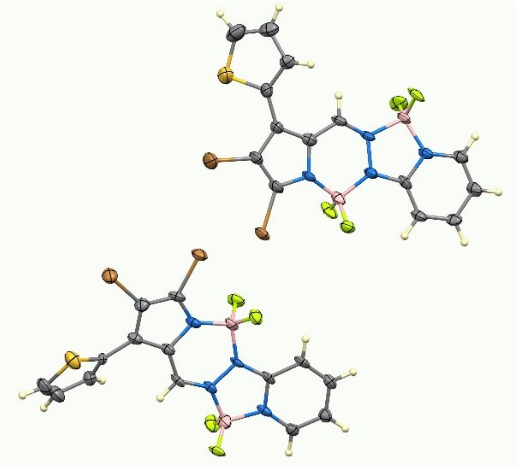

(1ca)

Crystal data

$C_{14}H_8B_2Br_2F_4N_4S \cdot 0.5(CH_2Cl_2)$	$Z = 4$
$M_r = 564.21$	$F(000) = 1092$
Triclinic, *P*-1	$D_x = 1.779$ Mg m^{-3}
$a = 9.117$ (7) Å	Ag $K\alpha$ radiation, $\lambda = 0.56086$ Å
$b = 14.673$ (14) Å	Cell parameters from 2938 reflections
$c = 16.599$ (16) Å	$\theta = 2.5$–16.6°
$\alpha = 108.321$ (14)°	$\mu = 2.20$ mm^{-1}
$\beta = 91.881$ (18)°	$T = 100$ K
$\gamma = 90.28$ (3)°	Plate, orange
$V = 2107$ (3) Å^3	0.22 × 0.20 × 0.02 mm

Data collection

Bruker D8 Venture DUO with Photon III C14 diffractometer	5207 reflections with $I > 2\sigma(I)$
Radiation source: IμS 3.0 microfocus	$R_{int} = 0.168$
ϕ and ω scans	$\theta_{max} = 19.5°$, $\theta_{min} = 2.1°$
Absorption correction: multi-scan *SADABS* (Krause *et al.*, 2015)	$h = -10 – 10$
$T_{min} = 0.822$, $T_{max} = 0.957$	$k = -17 – 17$
64251 measured reflections	$l = -19 – 19$
7426 independent reflections	

Refinement

Refinement on F^2	0 restraints
Least-squares matrix: full	Hydrogen site location: inferred from neighbouring sites
$R[F^2 > 2\sigma(F^2)] = 0.067$	H-atom parameters constrained
$wR(F^2) = 0.179$	$w = 1/[\sigma^2(F_o^2) + (0.0575P)^2 + 16.3018P]$ where $P = (F_o^2 + 2F_c^2)/3$
$S = 1.04$	$(\Delta/\sigma)_{max} < 0.001$
7426 reflections	$\Delta\rho_{max} = 1.37$ e Å^{-3}
523 parameters	$\Delta\rho_{min} = -1.28$ e Å^{-3}

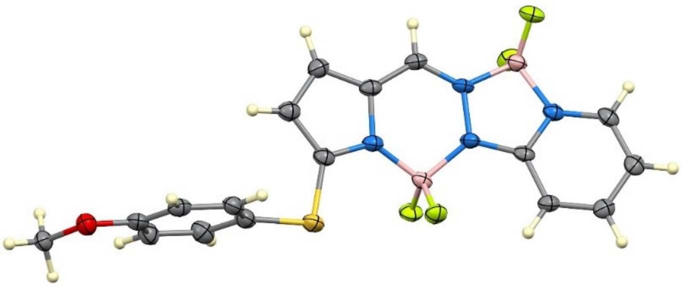

(1bj)

Crystal data

$C_{17}H_{13.91}B_2Cl_{0.09}F_4N_4OS$	$Z = 2$
$M_r = 423.21$	$F(000) = 431$
Triclinic, *P*-1	$D_x = 1.590$ Mg m^{-3}
$a = 7.1778$ (8) Å	Cu *K*α radiation, λ = 1.54184 Å
$b = 8.9232$ (18) Å	Cell parameters from 9873 reflections
$c = 14.9754$ (17) Å	θ = 5.3–74.3°
α = 101.046 (6)°	μ = 2.30 mm^{-1}
β = 100.789 (5)°	$T = 100$ K
γ = 104.359 (6)°	Lath fragment, yellow
$V = 883.8$ (2) Å^3	0.15 × 0.12 × 0.02 mm

Data collection

Bruker D8 Venture DUO with Photon III C14 diffractometer	3316 reflections with $I > 2\sigma(I)$
Radiation source: IμS 3.0 microfocus	$R_{int} = 0.047$
ϕ and ω scans	$\theta_{max} = 74.7°$, $\theta_{min} = 5.3°$
Absorption correction: multi-scan *SADABS* (Krause *et al.*, 2015)	$h = -8 - 8$
$T_{min} = 0.737$, $T_{max} = 0.955$	$k = -11 - 9$
29059 measured reflections	$l = -18 - 18$
3594 independent reflections	

Refinement

Refinement on F^2	0 restraints
Least-squares matrix: full	Hydrogen site location: inferred from neighbouring sites
$R[F^2 > 2\sigma(F^2)] = 0.045$	H-atom parameters constrained

$wR(F^2) = 0.115$	$w = 1/[\sigma^2(F_o^2) + (0.0472P)^2 + 1.1277P]$ where $P = (F_o^2 + 2F_c^2)/3$
$S = 1.06$	$(\Delta/\sigma)_{max} < 0.001$
3594 reflections	$\Delta\rho_{max} = 1.24$ e Å^{-3}
273 parameters	$\Delta\rho_{min} = -0.50$ e Å^{-3}

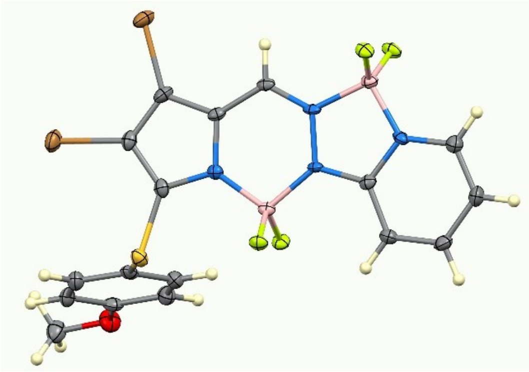

(1cb)

Crystal data

$C_{17}H_{12}B_2Br_2F_4N_4OS$	$F(000) = 564$
$M_r = 577.81$	$D_x = 1.920$ Mg m^{-3}
Monoclinic, $P2_1$	Cu $K\alpha$ radiation, $\lambda = 1.54184$ Å
$a = 12.6258$ (2) Å	Cell parameters from 9892 reflections
$b = 4.7201$ (1) Å	$\theta = 2.6$–$74.4°$
$c = 17.4560$ (3) Å	$\mu = 6.64$ mm^{-1}
$\beta = 106.159$ (2)°	$T = 100$ K
$V = 999.19$ (3) Å^3	Needle, colourless
$Z = 2$	0.28 × 0.04 × 0.01 mm

Data collection

Bruker D8 Venture DUO with Photon III C14 diffractometer	3776 reflections with $I > 2\sigma(I)$
Radiation source: IμS 3.0 microfocus	$R_{int} = 0.050$
ϕ and ω scans	$\theta_{max} = 74.6°$, $\theta_{min} = 2.6°$
Absorption correction: multi-scan *SADABS* (Krause *et al.*, 2015)	$h = -15 - 15$
$T_{min} = 0.776$, $T_{max} = 0.937$	$k = -5 - 5$
23766 measured reflections	$l = -21 - 21$

4023 independent reflections	

Refinement

Refinement on F^2	Hydrogen site location: inferred from neighbouring sites
Least-squares matrix: full	H-atom parameters constrained
$R[F^2 > 2\sigma(F^2)] = 0.024$	$w = 1/[\sigma^2(F_o^2) + (0.0179P)^2 + 0.7109P]$ where $P = (F_o^2 + 2F_c^2)/3$
$wR(F^2) = 0.054$	$(\Delta/\sigma)_{max} = 0.001$
$S = 1.06$	$\Delta\rho_{max} = 0.38$ e Å^{-3}
4023 reflections	$\Delta\rho_{min} = -0.36$ e Å^{-3}
281 parameters	Absolute structure: Flack x determined using 1555 quotients [(I+)-(I-)]/[(I+)+(I-)] (Parsons, Flack and Wagner, Acta Cryst. B69 (2013) 249-259).
1 restraint	Absolute structure parameter: 0.048 (10)

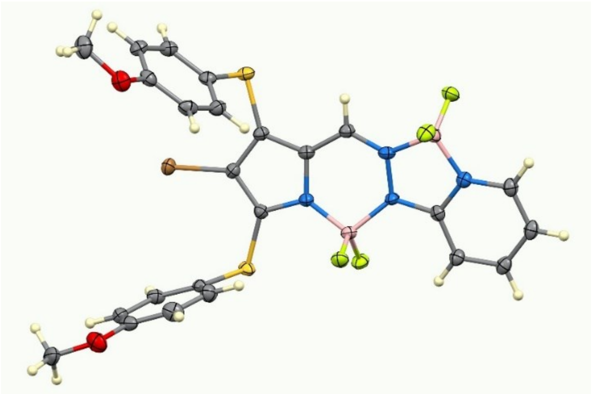

(1cc)

Crystal data

$C_{24}H_{19}B_2BrF_4N_4O_2S_2$	$Z = 2$
$M_r = 637.08$	$F(000) = 640$
Triclinic, P-1	$D_x = 1.660$ Mg m^{-3}
$a = 9.5212$ (11) Å	Cu $K\alpha$ radiation, $\lambda = 1.54184$ Å
$b = 10.3736$ (7) Å	Cell parameters from 9908 reflections
$c = 14.9510$ (11) Å	$\theta = 3.1$–$80.1°$
$\alpha = 88.637$ (3)°	$\mu = 4.28$ mm^{-1}
$\beta = 75.067$ (3)°	$T = 100$ K
$\gamma = 63.906$ (4)°	Plate, yellow

V = 1274.6 (2) Å^3	0.23 × 0.14 × 0.02 mm

Data collection

Bruker D8 Venture DUO with Photon III C14 diffractometer	4863 reflections with $I > 2\sigma(I)$
Radiation source: IμS 3.0 microfocus	R_{int} = 0.045
ϕ and ω scans	θ_{max} = 80.5°, θ_{min} = 3.1°
Absorption correction: multi-scan *SADABS* (Krause *et al.*, 2015)	h = -10 – 11
T_{min} = 0.691, T_{max} = 0.919	k = -13 – 13
24616 measured reflections	l = -18 – 19
5453 independent reflections	

Refinement

Refinement on F^2	0 restraints
Least-squares matrix: full	Hydrogen site location: inferred from neighbouring sites
$R[F^2 > 2\sigma(F^2)]$ = 0.034	H-atom parameters constrained
$wR(F^2)$ = 0.087	$w = 1/[\sigma^2(F_o^2) + (0.039P)^2 + 1.4141P]$ where $P = (F_o^2 + 2F_c^2)/3$
S = 1.04	$(\Delta/\sigma)_{max}$ = 0.001
5453 reflections	$\Delta\rho_{max}$ = 0.79 e Å^{-3}
354 parameters	$\Delta\rho_{min}$ = -0.56 e Å^{-3}
